# Photo‐Induced Halogen‐Atom Transfer: Generation of Halide Radicals for Selective Hydrohalogenation Reactions

**DOI:** 10.1002/chem.202201495

**Published:** 2022-06-13

**Authors:** Lilian Geniller, Marc Taillefer, Florian Jaroschik, Alexis Prieto

**Affiliations:** ^1^ ICGM Univ Montpellier, CNRS, ENSCM 34000 Montpellier France

**Keywords:** halogen atom transfer (XAT), hydrohalogenation, photochemistry, radical, silanes

## Abstract

The first photo‐mediated process enabling the generation of halide radicals by Halogen‐Atom Transfer (XAT) is described. Contrary to radical transformations involving XAT reactivity, which exploit stable carbon radicals, this unique approach uses 1,2‐dihaloethanes for the generation of unstable carbon radicals by XAT. These transient radicals then undergo β‐scission with release of ethylene and formation of more stable halide radicals which have been used in selective hydrohalogenations of a large number of unsaturated hydrocarbons, including Michael acceptors, unactivated alkenes and alkynes. This hydrohalogenation is tolerant of a broad range of functionalities and is believed to proceed through a radical‐chain manifold that propagates by the use of silane derivatives.

## Graphical abstract

**The first photo‐mediated process** enabling the generation of halide radicals by halogen‐atom transfer (XAT) is described. This novel reactivity pattern relies on the use of 1,2‐dihaloethanes for the generation of unstable carbon radicals by XAT, which after β‐scission, release halide radicals that have been used in selective hydrohalogenations of unsaturated hydrocarbons.

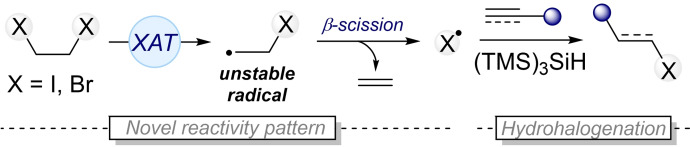

Halogenated organic compounds have an important place in organic chemistry, associated with their wide applicability as synthetic precursors.^[1]^ Among them, alkyl and alkenyl halides take part in a large number of organic transformations allowing the introduction of various functional groups. Alkyl halides readily undergo substitution processes with a large panel of nucleophiles, whereas alkenyl halides often serve as coupling partners in metal‐catalyzed cross‐coupling reactions.[^1^, ^2^] Both compound classes are also widely employed as precursors for organometallic reagents.^[3]^ Consequently, extensive efforts have been made for developing efficient protocols for their synthesis.^[4]^ The hydrohalogenation of olefins and alkynes is the most effective way to prepare such halides. Importantly, the regioselectivity of this reaction can be modulated: Markovnikov addition is observed under ionic conditions, whereas the radical hydrohalogenation promotes the formation of anti‐Markovnikov products (Figure 1A). However, this reaction is mainly restricted to the use of strongly corrosive and acidic hydrogen halides as halide sources, which drastically reduces the functional group tolerance.^[5]^ The discovery of novel mild halide sources compatible with a functional group tolerant hydrohalogenation still remains a challenge in organic synthesis, as shown by some few recent examples in the literature.^[6]^

**Figure 1:**
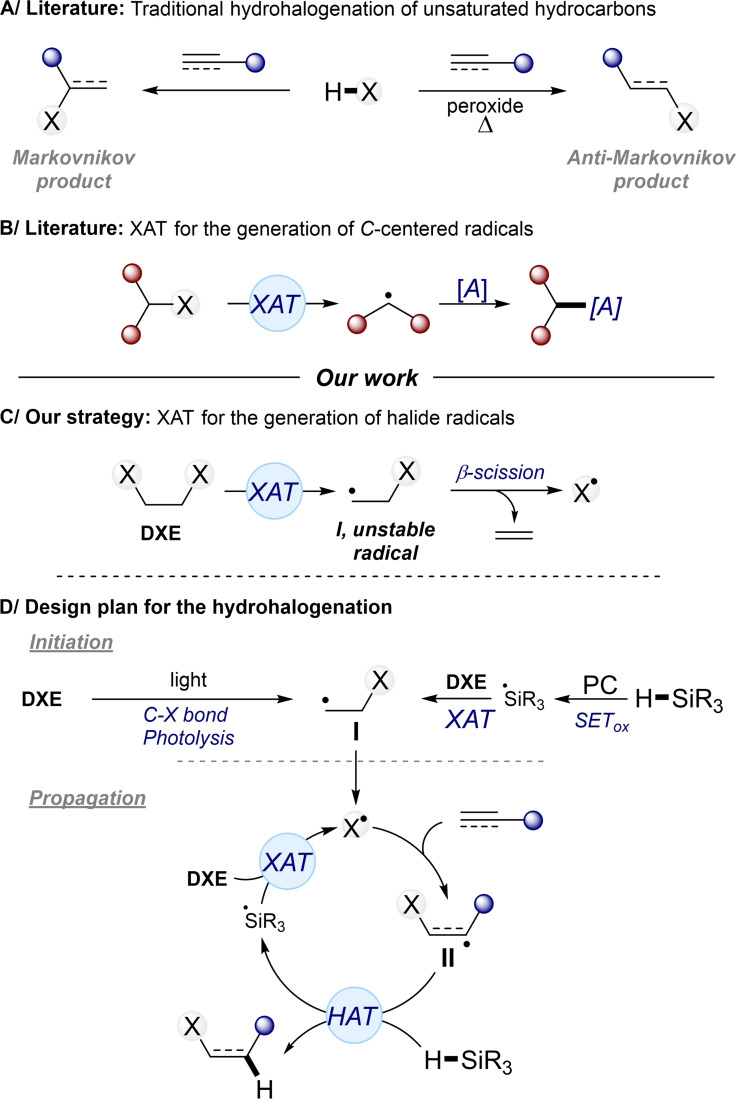
Design plan for hydrohalogenation via XAT strategy.

In radical chemistry, the halogen‐atom transfer (XAT) process is an important approach for the generation of *C*‐centered radicals (Figure 1B). XATs have been restricted for a long time to the ‘traditional’ radical chemistry, in which peroxide and tin derivatives were required for efficient reactions.^[7]^ More recently, XATs have found many applications in photo‐mediated reactions, opening new opportunities for constructing C−C bond products under mild and less toxic reaction conditions.^[8]^ However, the reported photo‐mediated XATs in the literature are exclusively narrowed to the formation and the exploitation of carbon radicals.^[8a]^

In this context, we wondered if it would be possible to develop an XAT strategy for the generation of radical halides which could be applied to the hydrohalogenation of unsaturated hydrocarbons. For reaching this goal, we considered the use of abundant and inexpensive 1,2‐dihaloethanes (DXE). Indeed, those compounds are well known as halide sources,^[9]^ notably for the synthesis of metal halides, such as divalent and trivalent lanthanide halides, in which β‐halide elimination is likely involved.[^10^, ^11^] Consequently, by extension, we envisaged that XAT on this type of compounds would lead to the formation of unstable *C*‐centered radical (**I**). A subsequent β‐scission step would deliver the expected radical halides with release of ethylene (Figure 1C). This process would be the key step in our designed hydrohalogenation which would follow a radical chain‐manifold (Figure 1D). The initiation could occur in the presence or in absence of a photocatalyst (PC). Without photocatalyst, the reaction would start by the C−X bond photolysis of DXE under light irradiation (Figure 1D, left part).^[12]^ In the presence of a PC, the single electron oxidation of the silane species leading to the silicon‐centered radical would initially occur. This radical would then react as a potent halide abstractor with DXE (Figure 1D, right part).[^12^, ^13^] Those two initiation pathways would lead to the formation of the radical species **I** that would deliver the radical halide by β‐scission. The addition of the radical halide onto unsaturated compounds would form the radical intermediate **II**. The latter would be involved in hydrogen atom transfer (HAT) with silane derivatives, yielding the final product along with a silicon radical species. Subsequent XAT between this silicon radical and DXE would sustain the radical chain process. Herein, we describe preliminary results demonstrating the feasibility of our design concept through the hydrohalogenation of a broad number of unsaturated hydrocarbons.

Preliminary studies have focused on the hydrobromination of ethyl acrylate (**1 a**) using DBE. After optimization, the best reaction conditions were obtained with DBE (5 equiv.), supersilane (TMS)_3_SiH (TTMSS, 1.2 equiv.) in ethyl acetateunder blue light irradiation at room temperature (Table 1, entry 1). Those conditions allowed the formation of **2 a** in a quantitative NMR yield, and the product was isolated in moderate yield due to its high volatility. Other sources of silanes resulted in decrease of yields (Table 1, entries 3–4). Variation of solvent did not significantly affect the reaction outcome, affording **2 a** in good to excellent yields (Table 1, entries 5–7). Decreasing the amount of DBE from 5 to 2 equivalents resulted in a drop in yield of **2 a**, however the use of DBE as solvent led to quantitative yield (Table 1, entries 8–9). The use of 4‐CzIPN as PC was not essential in this reaction (Table 1, entry 10). However, for some examples, the reaction was performed with the PC, leading to higher yields (Scheme 1 and 2). Finally, control experiments showed that both the light and the silane were essential to the reaction (Table 1, entries 11–12), supporting the proposed mechanism (Figure 1).

**Table 1:** Optimization of the hydrohalogenation reaction^[a]^

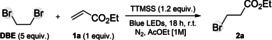		
Entry	Deviations from standard conditions	Yield **2 a** [%]^[b]^
1	none	quant. (55)^[c]^
2	TTMSS (1.5 equiv.)	quant.
3	Et_3_SiH (1.5 equiv.)	trace
4	Ph_3_SiH (1.5 equiv.)	43
5	MeCN	75
6	acetone	80
7	DCM	85
8	DBE as solvent	quant.
9	DBE (2 equiv.)	67
10	4‐CzIPN (5 mol%)	quant.
11	No TTMSS	N.R.
12	dark	N.R.

[a] Reaction conditions: **1 a** (0.3 mmol), DBE (1.5 mmol) in AcOEt (0.3 mL). [b] Yields were determined by ^1^H NMR using trichloroethylene as an internal standard. [c] Isolated yield. N.R.=no reaction. TTMSS=(TMS)_3_SiH.

**Scheme 1:**
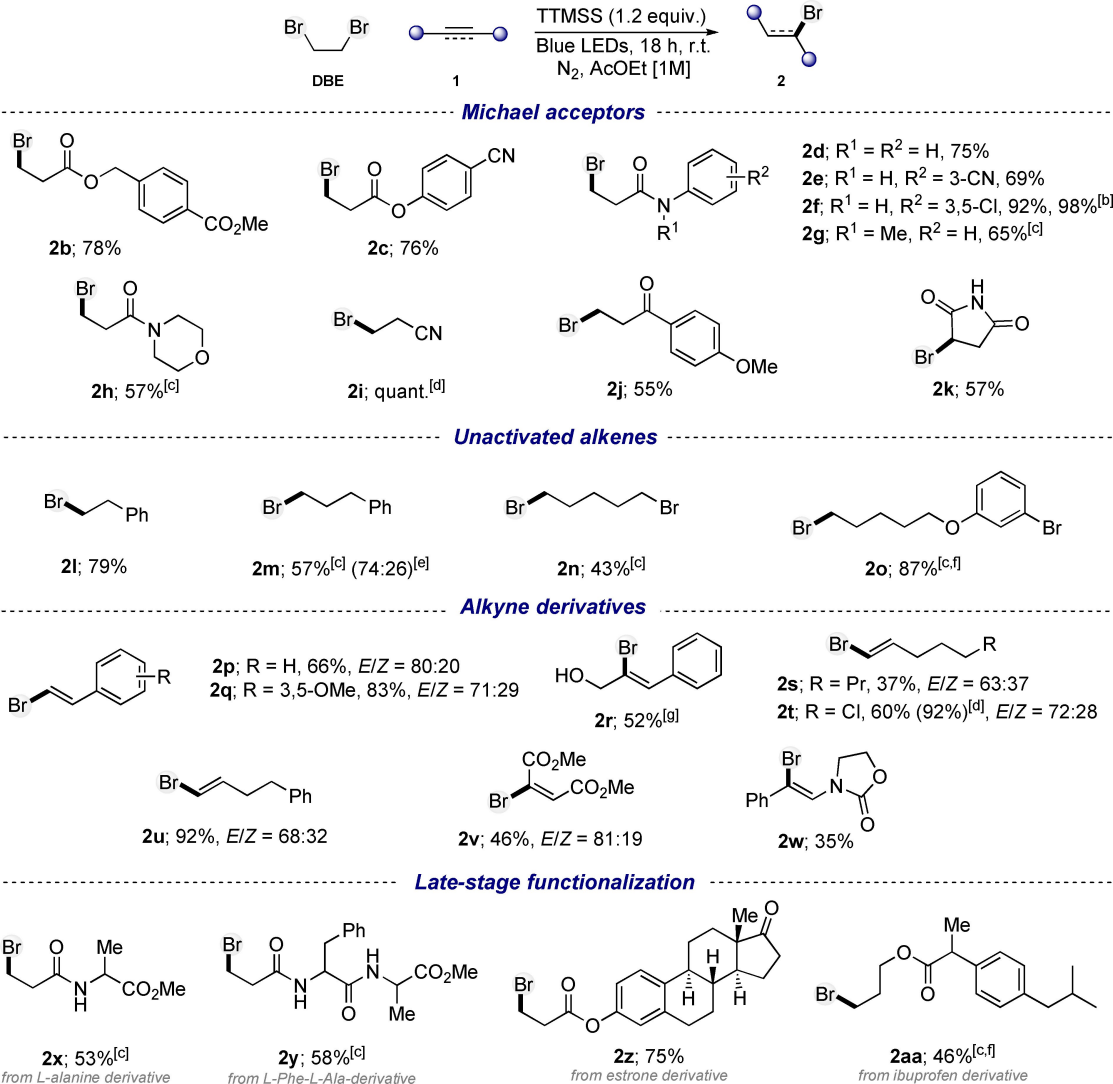
Scope of the hydrobromination of unsaturated compounds.^[a]^ [a] Reaction conditions: **1** (0.5 mmol), DBE (2.5 mmol), TTMSS (0.6 mmol) in AcOEt (0.5 mL). Yields refer to isolated products. [b] Reaction performed on 5 mmol scale. [c] Reaction performed with 4‐CzIPN (5 mol%). [d] NMR yield is given. [e] Anti‐Markovnikov/Markovnikov ratio. [f] Reaction performed in DBE. [g] Reaction performed in DCM.

**Scheme 2:**
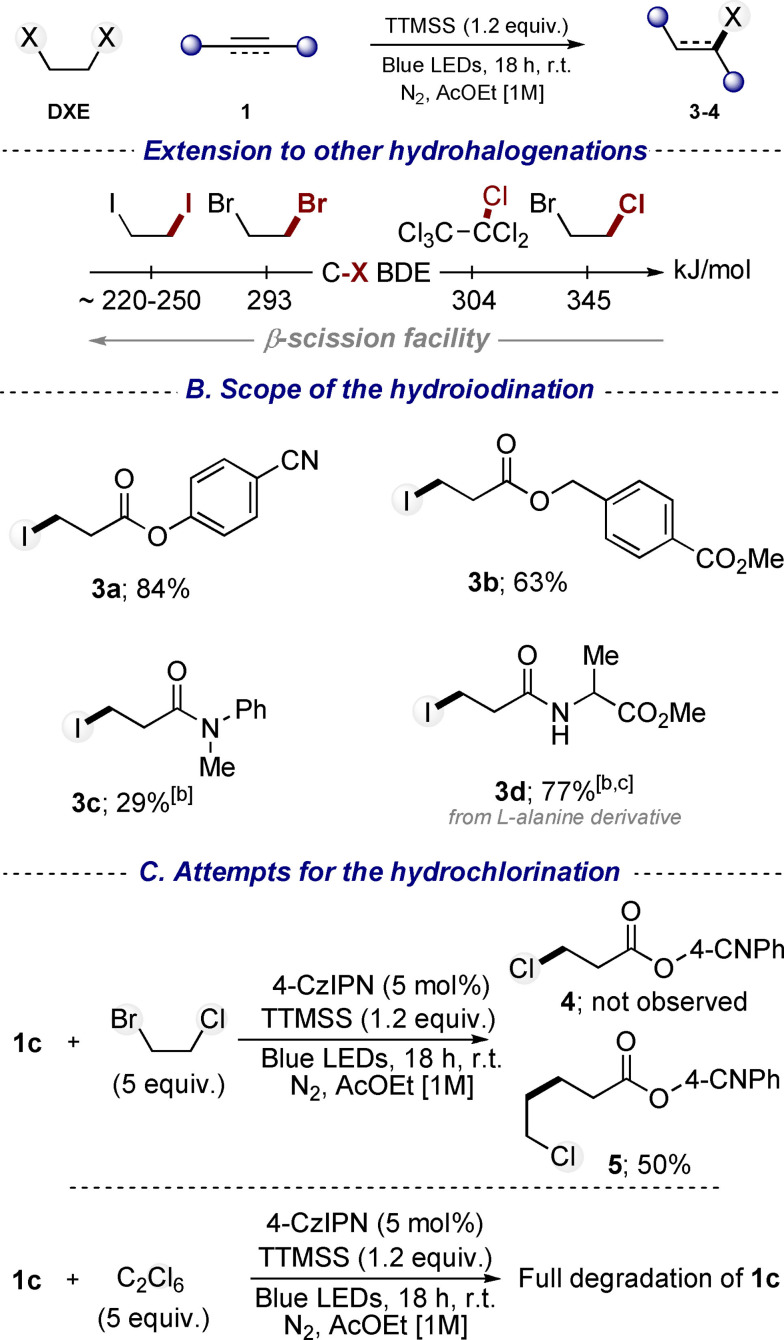
Scope of the other hydrohalogenation of unsaturated compounds.^[a]^ [a] Reaction conditions: **1** (0.5 mmol), DXE (2.5 mmol), TTMSS (0.6 mmol) in AcOEt (0.5 mL). Yields refer to isolated products. [b] Reaction performed with 4‐CzIPN (5 mol%). [c] Reaction performed in DCM.

With the optimized conditions in hands, we started to explore the scope of the hydrobromination on various unsaturated compounds (Scheme 1). First experiments focused on the hydrobromination of Michael acceptors. For instance, alkyl, benzyl, and aryl acrylates appeared to be suitable for the reaction yielding compounds **2 a**, **2 b** and **2 c** in good yields. The reaction was also highly effective on a large variety of acrylamides bearing diverse functional groups with diverse electronic natures (**2 d**–**2 h**), including *N*‐aryl, *N*,*N*‐methyl‐aryl and morpholinyl acrylamides. Additionally, acrylonitrile (**1 i**) participated efficiently in the reaction, affording **2 i** in excellent yield. Finally, acrylophenone derivative **1 j** and maleimide were found to be good candidates in this transformation, leading to products **2 j** and **2 k** in moderate yields (Scheme 1). We then investigated the reaction feasibility on unactivated alkenes including styrene and aliphatic alkenes. While the hydrobromination of styrene led to the exclusive formation of the anti‐Markovnikov product **2 l** in good yield, the reaction afforded a mixture of both regioisomers **2 m** with the allylbenzene **1 m**.^[14]^ Interestingly, alkene substrates bearing alkyl and aryl bromides (**1 n**–**1 o**) were well tolerated in the reaction forming respectively **2 n** and **2 o** in moderate to good yields. These examples demonstrate the selectivity of the XAT step in the process, likely due to the large concentration difference between the starting material and the DBE. Subsequently, the reaction was extended to alkynes (Scheme 1). First, the optimized conditions were applied to aryl alkynes, such as phenylacetylene derivatives **1 p**–**1 r**. The corresponding alkenyl bromides (**2 p**–**2 r**) were obtained in moderate to good yields as an *E*/*Z* mixture of stereoisomers and only compound **2 r**, bearing a native alcohol, was obtained as the single Z‐isomer. The hydrobromination reaction of other type of alkynes, such as acetylenedicarboxylate and alkyl alkynes also worked nicely, giving the corresponding products (**2 s**–**2 v**) in moderate to good yields. A scarce example of a photochemical transformation of ynamides was achieved with substrate **1 w**,^[15]^ providing product **2 w** in modest yield. Finally, the late‐stage functionalization of complex natural and bioactive molecules was explored. Pleasingly, the hydrobromination was found to be effective on Michael acceptor derivatives bearing amino acid, dipeptide or estrone moieties, affording hydrobrominated counterparts (**2 x**‐**2 z**) in moderate to good yields. Additionally, the late‐stage functionalization of ibuprofen derivative **1 aa**, bearing an unactivated alkene functionality, provided the brominated compound **2 aa** in moderate yield (Scheme 1). Those examples are particularly interesting as they might be engaged in further post‐functionalization reactions. Overall, this new hydrobromination reaction, that can be applied to alkenes (activated and unactivated) and alkynes, exhibits a good and broad functional group tolerance that includes esters, amides, nitriles, ketones, native alcohols, halides, ethers, oxazolidinones, amino acids. Moreover, in view of the practicality and mildness of the present method, a 5 mmol semi‐preparative gram scale synthesis of **2 f** could be achieved while maintaining a high isolated yield of 98 % (1.45 g) (Scheme 1).

We then evaluated the possibility of using other DXE substrates for achieving the hydroiodination and the very challenging hydrochlorination of unsaturated compounds (Scheme 2). While bond dissociation energies (BDE) of C−I bonds are lower than those for C−Br bonds (250 vs. 290 kJ/mol), BDE values of C−Cl bonds are much higher (around 345 kJ/mol) (Scheme 2A).^[16]^ This trend might affect the β‐scission feasibility, and therefore strongly influence the reaction outcome (Scheme 2A). To achieve our aims we decided to use DIE and 1,2‐bromochloroethane,^[17]^ as iodide and chloride sources, respectively. Application of the above optimized reaction conditions to the reaction of substrate **1 c** with DIE afforded product **3 a** in very high yield. This successful transfer of our strategy to hydroiodination was then extended to a range of unsaturated starting compounds. Products **3 b‐c** were obtained with slightly lower yields compared to the corresponding hydrobromination reactions. However, the hydroiodination of alanine derivative (**3 d**) resulted in better yield comparatively to the hydrobromination. Unfortunately, attempts for the hydroiodination of alkenes and unactivated alkynes led to inseparable mixtures of regioisomers and by‐products. The formation of side compounds might be linked to the lower stability of the C−I bond. Indeed, reduced products, likely resulting from XAT on final products, have been observed in several cases.

Initial attempts for the hydrochlorination have been conducted using 1,2‐bromochloroethane and substrate **1 c** (Scheme 2C). Unfortunately, the reaction did not afford compound **4** but compound **5**, resulting from an addition of a 2‐chloroethyl group onto the Michael acceptor, was isolated as the sole product. This result suggests that, in this particular case, the transient carbon radical obtained after XAT is stable enough not to undergo β‐scission. Nevertheless, this synthetically disappointing result, corroborates the proposed mechanism in which 2‐haloethyl radicals are formed. In view of this outcome, we envisaged using other chloride sources that would be likely more prone to β‐scission. Consequently, the reaction was explored using hexachloroethane, as the BDE of C−Cl bonds in this substrate is lower compared to 1,2‐bromochloroethane (304 vs. 345 kJ/mol). Unfortunately, the use of this reagent led exclusively to the degradation of the starting material **1 c** and did not furnish product **4** (Scheme 2C).

Finally, to gain further insights into the operating mechanism, deuterium labeling and radical trapping experiments were conducted (Scheme 3). First, the hydrobromination of **1 a** was repeated in DCM‐*d_2_*. In this case, the product **2 a** was obtained in 83 % yield without any deuterium incorporation. A second experiment was performed with the deuterated supersilane. The latter led to the quantitative formation of **2 a** with 84 % of d‐incorporation (Scheme 3), indicating that only the silane acts as hydrogen source in the reaction. This corroborates the mechanistic proposal in which the silane operates as hydrogen donation agent during the propagation process (Figure 1). Furthermore, the hydrobromination of **1 a** was also conducted in the presence of the radical scavenger 2,2,6,6‐tetramethyl‐1‐piperidinyloxy (TEMPO; 1 or 5 equiv.). Those conditions completely inhibited the formation of compound **2 a**, and two TEMPO adducts could be detected by MS analysis in both reactions (Scheme 3). Indeed, while TEMPO‐Br was not detected,^[18]^ TEMPO‐CH_2_CH_2_Br and TEMPO‐Si(TMS)_3_ were observed from the crude reaction mixtures. This result comforts the proposition that ⋅CH_2_CH_2_Br and ⋅Si(TMS)_3_ radicals are generated in the medium during the reaction, which is consistent with the mechanistic proposal. Finally, we explored the feasibility to perform the reaction by replacing the photoinitiation by classical radical initiation using common initiators such as AIBN or benzoyl peroxide ((BzO)_2_). Interestingly, while the reaction was not efficient with benzoyl peroxide at 50°C, providing the compound **2 a** in low yield, the reaction furnished **2 a** in quantitative yield at the same temperature with AIBN. Both initiators gave **2 a** in quantitative yield at 80 °C. Even though harder reaction conditions are required with radical initiators, compared to the photo initiation mode, for reaching the same result, those results might be helpful for researchers without photochemical equipment (see Supporting Information for more details).

**Scheme 3:**
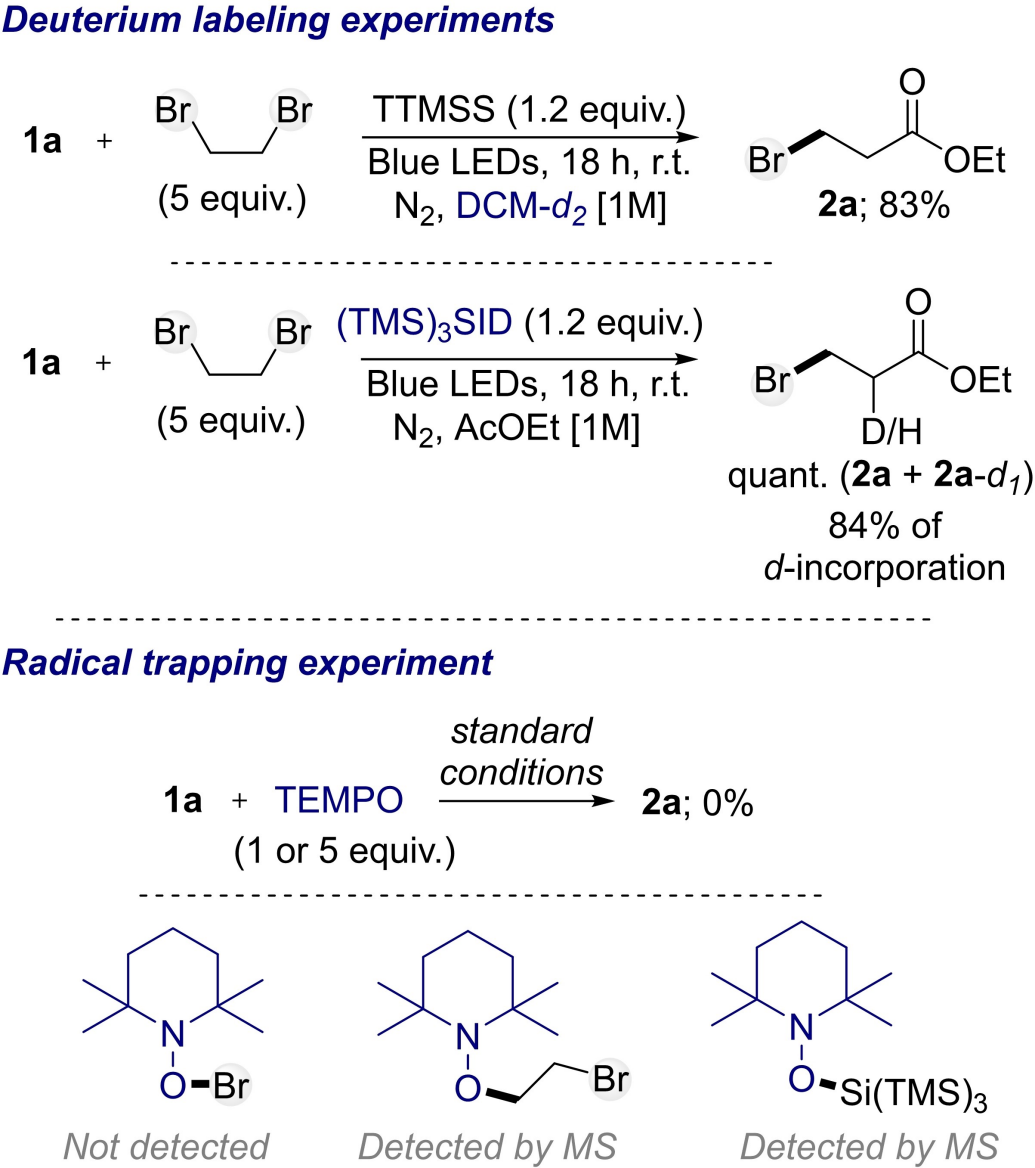
Mechanistic investigations.^[a]^ [a] Yields were determined by ^1^H NMR using trichloroethylene as an internal standard.

In summary, we have demonstrated that the XAT strategy can be applied to the generation of halide radicals. This hitherto unknown reactivity relies on the use of 1,2‐dihaloethanes, which after XAT form unstable radicals that undergo β‐scission with release of ethylene and formation of more stable halide radicals. This new reactivity pattern has been exploited for the regioselective hydroiodination and hydrobromination of various unsaturated derivatives, displaying high functional group tolerance due to the absence of corrosive acids. Unfortunately, this strategy has so far not been suitable for achieving the challenging hydrochlorination reaction. Ongoing studies focus on the exploitation of this novel reactivity pattern in other radical transformations involving XAT steps.

## Conflict of interest

The authors declare no conflict of interest.

## Supporting information

As a service to our authors and readers, this journal provides supporting information supplied by the authors. Such materials are peer reviewed and may be re‐organized for online delivery, but are not copy‐edited or typeset. Technical support issues arising from supporting information (other than missing files) should be addressed to the authors.

Supporting Information

## Data Availability

The data that support the findings of this study are available in the supplementary material of this article.

## References

[1] R. C. Larock, Comprehensive Organic Transformations: A Guide to Functional Group Preparations, Wiley-VCH, **2018**, 3rd ed.

[2a] *Metal-catalyzed cross-coupling reactions*; A. De Meijer, F. Diederich, Eds. Wiley-VCH Verlag GmbH & Co. KGaA: **2004**, Vols. 1 & 2;

[2b] K. C. Nicolaou , P. G. Bulger , D. Sarlah , Angew. Chem. Int. Ed. 2005, 44, 4442–4489; 10.1002/anie.200500368 15991198

[3] Organometallics in Synthesis, Third Manual; Schlosser, M., Ed.; John Wiley & Sons, Inc.: **2013**.

[4a] A. Varenikov , E. Shapiro , M. Gandelman , Chem. Rev. 2021, 121, 412–484; 33200917 10.1021/acs.chemrev.0c00813 PMC7884003

[4b] D. A. Petrone , J. T. Ye , M. Lautens , Chem. Rev. 2016, 116, 8003–8104; 27341176 10.1021/acs.chemrev.6b00089

[4c] T.-Y. Luh , M.-K. Leung , K.-T. Wong , Chem. Rev. 2000, 100, 3187–3204. 11749317 10.1021/cr990272o

[5a] W. I. Markownikoff , Justus Liebigs Ann. Chem. 1870, 153, 228–259;

[5b] M. S. Kharasch , F. R. Mayo , J. Am. Chem. Soc. 1933, 55, 2468–2496;

[5c] M. Galli , C. J. Fletcher , M. del Pozo , S. M. Goldup , Org. Biomol. Chem. 2016, 14, 5622–5626. 10.1039/c6ob00692b 27185636

[6a] X. Li , J. Jin , P. Chen , G. Liu , Nat. Chem. 2022, 14, 425–432; 35102326 10.1038/s41557-021-00869-x

[6b] D. A. Cruz , V. Sinka , P. de Armas , H. S. Steingruber , I. Fernández , V. S. Martín , P. O. Miranda , J. I. Padrón , Org. Lett. 2021, 23, 6105–6109; 34318671 10.1021/acs.orglett.1c02186 PMC8397429

[6c] P. Yu , A. Bismuto , B. Morandi , Angew. Chem. Int. Ed. 2020, 59, 2904–2910; 10.1002/anie.201912803 PMC7028031 31769578

[6d] W. Chen , M. Oestreich , Org. Lett. 2019, 21, 4531–4534; 31150253 10.1021/acs.orglett.9b01431

[6e] M. R. Uehling , R. P. Rucker , G. Lalic , J. Am. Chem. 2014, 136, 8799–8803; 10.1021/ja503944n 24896663

[6f] D. J. Wilger , J. M. Grandjean , T. R. Lammert , D. A. Nicewicz , Nat. Chem. 2014, 6, 720–726. 25054943 10.1038/nchem.2000

[7a] H. G. Kuivila , L. W. Menapace , J. Org. Chem. 1963, 28, 2165–2167;

[7b] B. Giese , Angew. Chem. Int. Ed. 1983, 22, 753–764;

[8] For a review, see:

[8a] F. Juliá , T. Constantin , D. Leonori , Chem. Rev. 2022, 122, 2292–2352; 34882396 10.1021/acs.chemrev.1c00558

[8b] For recent selected examples: S. M. Hell , C. F. Meyer , S. Ortalli , J. B. I. Sap , X. Chen , V. Gouverneur , Chem. Sci. 2021, 12, 12149–12155; 34667580 10.1039/d1sc03421a PMC8457377

[8c] P. J. Deneny , R. Kumar , M. J. Gaunt , Chem. Sci. 2021, 12, 12812–12818; 34703568 10.1039/d1sc04554g PMC8494037

[8d] Y. Wang , L.-F. Deng , X. Zhang , Z.-D. Mou , D. Niu , Angew. Chem. Int. Ed. 2021, 60, 2155–2159; 10.1002/anie.202012503 33022829

[8e] W. Liu , M. N. Lavagnino , C. A. Gould , J. Alcázar , D. W. C. MacMillan , Science 2021, 374, 1258–1263; 34762491 10.1126/science.abl4322 PMC8926084

[8f] N. W. Dow , A. Cabré , D. W. C. MacMillan , Chem 2021, 7, 1827–1842; 34423174 10.1016/j.chempr.2021.05.005 PMC8372964

[8g] T. Constantin , M. Zanini , A. Regni , N. S. Sheikh , F. Juliá , D. Leonori , Science 2020, 367, 1021–1026; 32108109 10.1126/science.aba2419

[8h] T. Kerackian , A. Reina , D. Bouyssi , N. Monteiro , A. Amgoune , Org. Lett. 2020, 22, 2240–2245; 32148046 10.1021/acs.orglett.0c00442

[8i] C. Le , T. Q. Chen , T. Liang , P. Zhang , D. W. C. MacMillan , Science 2018, 360, 1010–1014; 29853683 10.1126/science.aat4133 PMC6607890

[8j] S. M. Hell , C. F. Meyer , A. Misale , J. B. I. Sap , K. E. Christensen , M. C. Willis , A. A. Trabanco , V. Gouverneur , Angew. Chem. Int. Ed. 2020, 59, 11620–11626; 10.1002/anie.202004070 PMC7384135 32286720

[9a] X. Dong , J. L. Roeckl , S. R. Waldvogel , B. Morandi , Science 2021, 371, 507–514; 33510026 10.1126/science.abf2974

[9b] Y. Liang , F. Lin , Y. Adeli , R. Jin , N. Jiao , Angew. Chem. Int. Ed. Engl. 2019, 58, 4566–4570; 30664331 10.1002/anie.201814570

[10a] P. Girard , J. L. Namy , H. B. Kagan , J. Am. Chem. Soc. 1980, 102, 2693–2698;

[10b] M.-I. Lannou , F. Hélion , J.-L. Namy , Tetrahedron 2003, 59, 10551–10565.

[11] M. Lal , J. Mönig , K. D. Asmus , Free Radic. Res. Commun. 1986, 1, 235–241. 3333033 10.3109/10715768609051633

[12] For recent examples reporting C−X bonds photolysis under light irradiation, see:

[12a] Y. Cheng , C. Muck-Lichtenfeld , A. Studer , Angew. Chem. Int. Ed. 2018, 57, 16832–16836; 10.1002/anie.201810782 PMC6470957 30332527

[13a] C. Chatgilialoglu , C. Ferreri , Y. Landais , V. I. Timokhin , Chem. Rev. 2018, 118, 6516–6572; 29938502 10.1021/acs.chemrev.8b00109

[13b] C. Chatgilialoglu , V. I. Timokhin , Adv. Organomet. Chem. 2008, 57, 117–181;

[13c] C. Chatgilialoglu , Chem. Rev. 1995, 95, 1229–1251.

[14] In the case of **1 m**, a mixture of regioisomers was obtained, which might result from the in situ formation of HBr. Indeed, bromine radical might be involved in a HAT with benzylic-allylic position of **1 m**, leading finally to the formation of the Markovnikov product.

[15a] C. Mahe , K. Cariou , Adv. Synth. Catal. 2020, 362, 4820–4832;

[15b] T.-D. Tan , Z.-S. Wang , P.-C. Qian , L.-W. Ye , Small Methods 2021, 5, 2000673. 10.1002/smtd.202000673 34927818

[16a] Y.-R. Luo, Handbook of Bond Dissociation Energies in Organic Compounds, 1st Edition.; CRC Press: Boca Raton, FL, 2002;

[16b] X.-S. Xue , P. Ji , B. Zhou , J.-P. Cheng , Chem. Rev. 2017, 117, 8622–8648; 28281752 10.1021/acs.chemrev.6b00664

[16c] S. J. Blanksby , G. B. Ellison , Acc. Chem. Res. 2003, 36, 255–263. 12693923 10.1021/ar020230d

[17] 1,2-Bromochloroethane was chosen as it has been described that TTMSS can hardly activate unactivated chlorinated aliphatic hydrocarbons by XAT, see: H. A. Sakai , W. Liu , C. C. Le , D. W. C. MacMillan , J. Am. Chem. Soc. 2020, 142, 11691–11697. 32564602 10.1021/jacs.0c04812 PMC7750884

[18] I. I. Roslan , H. Zhang , K.-H. Ng , S. Jaenicke , G.-K. Chuah , Adv. Synth. Catal. 2021, 363, 1007–1013.

