# Supplementary material for: Photo‐Induced Halogen‐Atom Transfer: Generation of Halide Radicals for Selective Hydrohalogenation Reactions
Source: Chemistry. 2022 Jun 13;28(43):e202201495. doi: 10.1002/chem.202201495 (PMC9401045; doi:10.1002/chem.202201495)
Supplement: Supplementary file 1 — Supporting Information [file CHEM-28-0-s001.pdf]

Chemistry–A European Journal

Supporting Information

Photo-Induced Halogen-Atom Transfer: Generation of Halide Radicals for Selective Hydrohalogenation Reactions

Lilian Geniller, Marc Taillefer, Florian Jaroschik, and Alexis Prieto*

Table of content

A. General information S2

B. Synthesis of compounds **1** S3

C. Characterization of compounds **2** S4

D. Mechanistic investigations S10

E. References S13

F. NMR Spectra of compounds **1**, **2**, **3**, **4**, **5** S14

A. General information

^{1}H, ^{13}C and ^{19}F spectra were recorded on a Bruker®AC-400 MHz spectrometer (400 MHz, 101 MHz, and 376 MHz respectively) at ambient temperature. The peaks were internally referenced to residual undeuterated solvent signal ($CDCl_3$: 7.26 ppm (^{1}H NMR), 77.16 ppm (^{13}C NMR)). The following abbreviations were used to explain multiplicities: s = singlet, d = doublet, t = triplet, q = quartet, m = multiplet, br = broad). All ^{19}F NMR spectra are reported in ppm relative to $CFCl_3$. NMR yields were determined by using respectively trifluorotoluene or trifluoroanisole for ^{19}F NMR yields and trichloroethylene for ^{1}H NMR yields as internal standards. HRMS (Q-TOF) were performed on a JEOL JMS-DX300 spectrometer (3 keV, xenon) in a m-nitrobenzylalcohol matrix. All reactions were performed under an argon atmosphere in a sealed reaction vial. Reactions were monitored by analytical thin layer chromatography (TLC) using commercial sheets precoated (0.2 mm layer thickness) with silica gel 60F254 (Macherey-Nagel). Product purification by flash column chromatography was performed using Macherey-Nagel Silica Gel (40-63 µm). Solvents and reagents were obtained from commercial sources and used as received.

Materials. Commercial grade reagents and solvents were purchased from Sigma-Aldrich, Fluka, Alfa Aesar, Fluorochem, SynQuest at the highest commercial quality and used without further purification, unless otherwise stated. Starting materials TTMSS, DBE, DIE, 1,2-brchloroethane, hexachloroethane, **1a**, **1i**, **1k**, **1l**, **1m**, **1n**, **1p**, **1q**, **1r**, **1s**, **1t**, **1u**, **1v**, **1w** are commercially available. They were purchased from Sigma-Aldrich, Alfa Aesar, Fluorochem, TCI Europe, and used without further purification.

Photochemical equipment. Reactions were performed in the EvoluChemTM PhotoRedOx reactor fitted with the selected EvoluChem lights (365 nm - 18W, 405 nm - 18W). The equipment was purchased from Interchim. For detailed specification on the reactor and the light used see: http://www.shigematsu-bio.com/wordpress/wp-content/uploads/2018/10/EvoluChem-Photoredox-Brochure-content-Ver-1.0_201810.pdf.

B. Synthesis of compounds 1

Starting materials **1d**,[1] **1e**,[2] **1f**,[3] **1g**,[4] **1h**,[5] **1j**,[6] **1o**,[7] **1w**,[8] **1x**,[9] **1z**,[10] **1aa**,[11] were synthesized according described procedures.

Methyl 4-((acryloyloxy)methyl)benzoate (1b): To a stirred solution of methyl 4-(hydroxymethyl)benzoate (1.6 g, 10 mmol, 1 equiv.) and triethylamine (2 mL, 1.5 equiv.) in dry dichloromethane (1 M) was added dropwise acryloyl chloride (850 µL, 1.05 equiv.) at 0 °C under atmosphere of nitrogen. The mixture was kept below 0 °C for 30 min and was then stirred at room temperature overnight. The reaction mixture was quenched with water, and extracted with water and aq. NH_4Cl The organic layer was dried over $MgSO_4$, and concentrated under reduced pressure. The residue was purified by silica gel column chromatography to give the pure compound as a yellow oil (1.6 g, 74%).**^{1}H NMR** (400 MHz, Chloroform-d) δ 8.05 – 8.01 (m, 2H), 7.45 – 7.41 (m, 2H), 6.46 (dd, J = 17.3, 1.4 Hz, 1H), 6.18 (dd, J = 17.3, 10.4 Hz, 1H), 5.87 (dd, J = 10.4, 1.4 Hz, 1H), 5.24 (s, 2H), 3.91 (s, 3H). **^{13}C NMR** (101 MHz, Chloroform-d) δ 166.8, 165.9, 141.0, 131.6, 130.0, 130.0, 128.1, 127.8, 65.6, 52.3. **HRMS (ASAP):** Calcd for $C_{12}H_{12}O_4$ [M+H, 100 %]: 221.0808, found 221.0810.

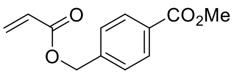

General procedure for the synthesis of acrylamides and aryl acrylates.

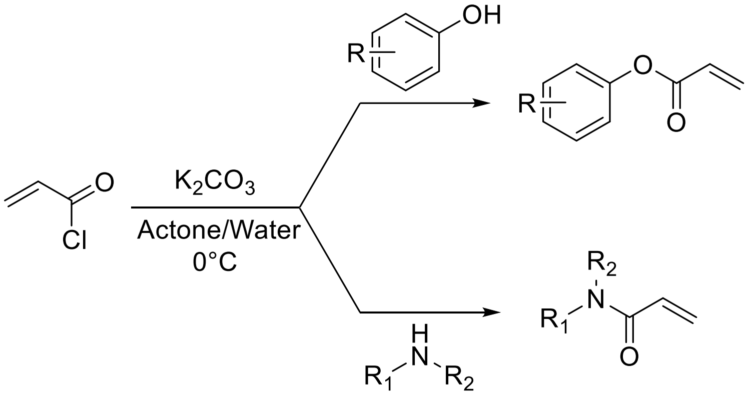

To a stirred suspension of potassium carbonate (2 equiv.) in water/acetone (1/4, 0.4 M) was added acryloyl chloride (1,5 equiv.) at 0 °C under atmosphere of nitrogen. The corresponding phenol or amine (1 equiv.) was then added dropwise to the mixture and stirred during 3 h at 0 °C. After filtration, the mixture was concentrated under reduced pressure and extracted three times with dichloromethane. The organic layer was dried over $MgSO_4$, filtration, and evaporation of the solvent. The residue was purified by silica gel column chromatography (eluent pentane/AcOEt).

4-cyanophenyl acrylate (1c): white solid (2,93 g, 74%). **^{1}H NMR** (400 MHz, Chloroform-d) δ 7.73 – 7.69 (m, 2H), 7.31 – 7.27 (m, 2H), 6.65 (dd, J = 17.3, 1.1 Hz, 1H), 6.32 (dd, J = 17.3, 10.5 Hz, 1H), 6.09 (dd, J = 10.5, 1.1 Hz, 1H). **^{13}C NMR** (101 MHz, Chloroform-d) δ 163.6, 153.9, 133.9, 133.7, 127.3, 122.8, 118.3, 109.8. **HRMS (ASAP):** Calcd. for $C_{10}H_7NO_2$ [M+H, 100 %]: 174.0550, found 174.0546.

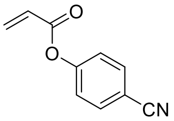

methyl acryloylphenylalanylalaninate (1y): white solid (180 mg, 45%). **^{1}H NMR** (400 MHz, Chloroform-d) δ 7.23 (dt, J = 13.6, 5.2 Hz, 5H), 7.04 (d, J = 7.3 Hz, 2H), 6.25 (dd, J = 17.0, 1.4 Hz, 1H), 6.12 (dd, J = 17.0, 10.1 Hz, 1H), 5.61 (dd, J = 10.1, 1.4 Hz, 1H), 4.88 (q, J = 7.1 Hz, 1H), 4.46 (p, J = 7.2 Hz, 1H), 3.70 (s, 3H), 3.09 (qd, J = 13.8, 7.0 Hz, 1H), 1.33 (d, J = 7.2 Hz, 3H). **^{13}C NMR** (101 MHz, Chloroform-d) δ 172.9, 171.2, 165.5, 136.6, 130.5, 129.5, 128.61, 127.2, 127.0, 54.6, 52.5, 48.3, 38.6, 18.0. **HRMS (ASAP):** Calcd. for $C_{16}H_{20}N_2O_4$ [M+H, 100 %]: 253.9807, found 253.9811.

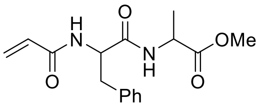

C. Characterization of compounds 2

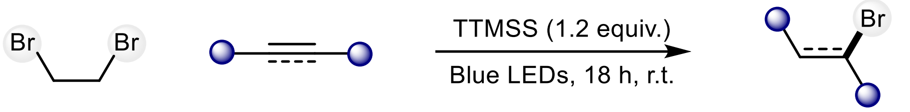

DBE **1** **2**

General Procedure. (*0.5 mmol scale reaction*). In a vial, TTMSS (185 μL, 0.6 mmol, 1.2 equiv.) was added to a solution of the selected unsaturated hydrocarbon **1** (0.5 mmol, 1 equiv.) and the DBE (215 μL, 2.5 mmol, 5 equiv.) in AcOEt (0.5 mL). The reaction mixture was degassed via a freeze pump thaw procedure, and then placed in the EvoluChemTM PhotoRedOx reactor fitted with the 450 nm (18W) EvoluChem light. The reaction was stirred under light irradiation at room temperature for 18 h. Then the reaction mixture was concentrated *in vacuo* and the residue was purified by flash chromatography (silica gel, appropriate mixture of hexane/ethyl acetate) to afford the corresponding product **3**. *The typical set-up experiment is showed in* ***Figure S1****.*

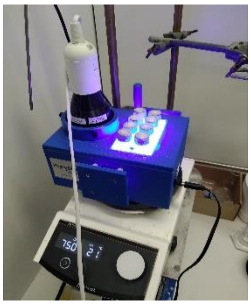

Figure S1. *Set up*

Ethyl 3-bromopropanoate (2a): Colorless oil (50 mg, 55%).**^{1}H NMR** (400 MHz, $CDCl_3$) δ 4.19 (q, J = 7.2 Hz, 2H), 3.58 (t, J = 6.9 Hz, 2H), 2.91 (t, J = 6.9 Hz, 2H), 1.28 (t, J = 7.2 Hz, 3H). ^{13}C NMR (101 MHz, $CDCl_3$) δ 170.7, 61.1, 37.9, 26.1, 14.3. Spectroscopic data obtained are consistent with the data reported in the literature.[12]

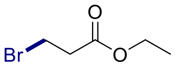

Methyl 4-(((3bromopropanoyl)oxy)methyl)benzoate (2b): Colorless oil (117 mg, 78%). **^{1}H NMR** (400 MHz, $CDCl_3$) δ 8.02 (d, J = 8.6 Hz, 2H), 7.41 (dd, J = 8.1, 0.6 Hz, 2H), 5.20 (s, 2H), 3.90 (s, 3H), 3.58 (t, J = 6.8 Hz, 2H), 2.98 (t, J = 6.7 Hz, 2H). **^{13}C NMR** (101 MHz, $CDCl_3$) δ 170.3, 166.7, 140.6, 130.1, 129.9, 127.9, 66.0, 52.3, 37.7, 25.8. **HRMS (ESI):** Calcd. for $C_{12}H_{14}BrO_4$ [M+H$^+$, 100 %]: 301.0070, found 301.0071.

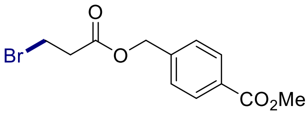

4-cyanophenyl 3-bromopropanoate (2c): Slightly yellow oil (97 mg, 76%). **^{1}H NMR** (400 MHz, $CDCl_3$) δ 7.71 (d, J = 8.9 Hz, 2H), 7.27 (d, J = 9.0 Hz, 2H), 3.69 (t, J = 6.6 Hz, 2H), 3.22 (t, J = 6.6 Hz, 2H). **^{13}C NMR** (101 MHz, $CDCl_3$) δ 168.4, 153.7, 133.8, 122.8, 118.2, 110.1, 37.8, 25.4. **HRMS (ESI):** Calcd. for $C_{10}H_9BrNO_2$ [M+H$^+$, 100 %]: 253.9811, found 253.9807.

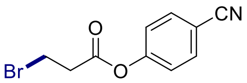

3-bromo-*N*-phenylpropanamide (2d): White solid (80 mg, 70%). **^{1}H NMR** (400 MHz, $CDCl_3$) δ 7.54 – 7.49 (m, 2H), 7.48 (brs, 1H), 7.32 (dd, *J* = 8.5, 7.5 Hz, 2H), 7.21 – 7.08 (m, 1H), 3.70 (t, *J* = 6.6 Hz, 2H), 2.94 (t, *J* = 6.6 Hz, 2H). **^{13}C NMR** (101 MHz, $CDCl_3$) δ 168.2, 137.5, 129.2, 124.9, 120.3, 40.8, 27.2. Spectroscopic data obtained are consistent with the data reported in the literature.[13]

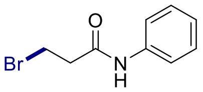

3-bromo-*N*-phenylpropanamide (2e): Off-white solid (87 mg, 69%). **^{1}H NMR** (400 MHz, $CDCl_3$) δ 8.15 (s, 1H), 7.98 (s, 1H), 7.75 (dt, J = 7.5, 2.1 Hz, 1H), 7.46 – 7.35 (m, 2H), 3.69 (t, J = 6.4 Hz, 2H), 2.99 (t, J = 6.4 Hz, 2H). **^{13}C NMR** (101 MHz, $CDCl_3$) δ 168.9, 138.6, 130.1, 128.1, 124.4, 118.7, 112.8, 40.4, 26.9. **HRMS (ESI):** Calcd. for $C_{10}H_{10}BrN_2O$ [M+H$^+$, 100 %]: 252.9971, found 252.9976.

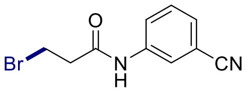

3-bromo-*N*-(3,5-dichlorophenyl)propanamide (2f): White solid (on 0.5 mmol: 136 mg, 92%; on 5 mmol: 1.45 g, 98%).). **^{1}H NMR** (400 MHz, $CDCl_3$) δ 7.90 (s, 1H), 7.47 (brs, 2H), 7.10 (t, *J* = 1.8 Hz, 1H), 3.68 (t, *J* = 6.4 Hz, 2H), 2.96 (t, *J* = 6.4 Hz, 2H). **^{13}C NMR** (101 MHz, $CDCl_3$) δ 168.8, 139.2, 135.4, 124.9, 118.6, 40.5, 26.7. **HRMS (ESI):** Calcd. for $C_{17}H_{16}NO_2$ [M+H$^+$, 100 %]: 295.9239, found 295.9234.

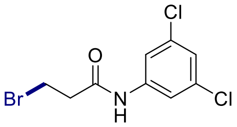

3-bromo-*N*-methyl-*N*-phenylpropanamide (2g): Obtained following the GP, except that the reaction was performed in presence of 4-CzIPN (5 mol%, 20 mg). Slightly yellow oil (79 mg, 65%). **^{1}H NMR** (400 MHz, $CDCl_3$) δ 7.45 – 7.37 (m, 2H), 7.37 – 7.31 (m, 1H), 7.21 – 7.02 (m, 2H), 3.55 (t, *J* = 6.8 Hz, 2H), 3.26 (s, 3H), 2.62 (t, *J* = 6.8 Hz, 2H). **^{13}C NMR** (101 MHz, $CDCl_3$) δ 169.8, 143.3, 130.0, 128.2, 127.4, 37.4, 37.1, 27.6. Spectroscopic data obtained are consistent with the data reported in the literature.[13]

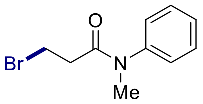

3-bromo-1-morpholinopropan-1-one (2h): Obtained following the GP, except that the reaction was performed in presence of 4-CzIPN (5 mol%, 20 mg). Slightly yellow oil (63 mg, 57%). **^{1}H NMR** (400 MHz, $CDCl_3$) δ 3.86 – 3.56 (m, 8H), 3.56 – 3.34 (m, 2H), 2.89 (t, *J* = 7.1 Hz, 2H). **^{13}C NMR** (101 MHz, $CDCl_3$) δ 168.7, 66.9, 66.7, 46.0, 42.2, 36.2, 27.2. **HRMS (ESI):** Calcd for $C_7H_{13}BrNO_2$ [M+H$^+$, 100 %]: 222.0124, found 222.0129.

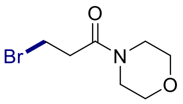

3-bromopropanenitrile (2i): The product was obtained in quantitative NMR yield. However, the latter was not isolated due to its high volatility. Signals reported have been extracted from the crude sample. **^{1}H NMR** (400 MHz, $CDCl_3$) δ 3.53 (t, *J* = 6.7 Hz, 2H), 2.98 (t, *J* = 6.7 Hz, 2H). **^{13}C NMR** (101 MHz, $CDCl_3$) δ 117.2, 24.2, 21.9. Spectroscopic data obtained are consistent with the data reported in the literature.[14]

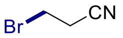

3-bromo-1-(4-methoxyphenyl)propan-1-one (2j): Slightly yellow solid (67 mg, 55%). **^{1}H NMR** (400 MHz, $CDCl_3$) δ 7.93 (d, J = 8.9 Hz, 1H), 6.94 (d, J = 8.9 Hz, 1H), 3.87 (s, 3H), 3.73 (t, J = 6.9 Hz, 2H), 3.51 (t, J = 6.9 Hz, 2H). **^{13}C NMR** (101 MHz, $CDCl_3$) δ 195.6, 164.0, 130.5, 129.5, 114.0, 55.7, 41.3, 26.3. Spectroscopic data obtained are consistent with the data reported in the literature.[15]

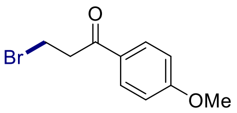

3-bromopyrrolidine-2,5-dione (2k): Colorless oil (51 mg, 57%). Obtained along with 5-10% of starting material. **2k** is not stable on silica gel, giving back the starting material over the purification process. Attempts for the purification of **2k** on neutralized silica gel with 1% of NEt_3 led to the full degradation to starting material. **^{1}H NMR** (400 MHz, $CDCl_3$) δ 9.16 (s, 1H), 4.72 (dd, *J* = 8.6, 3.5 Hz, 1H), 3.76 – 3.35 (m, 1H), 3.28 – 2.93 (m, 1H). **^{13}C NMR** (101 MHz, $CDCl_3$) δ 173.9, 173.9, 41.0, 36.1. **HRMS (ESI):** Calcd. for $C_4H_5BrNO_2$ [M+H$^+$, 100 %]: 177.9498, found 177.9496.

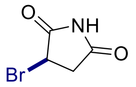

(3-bromoethyl)benzene (2l): Colorless oil (73 mg, 79%); **^{1}H NMR** (400 MHz, $CDCl_3$) δ 7.41 – 7.28 (m, 2H), 7.28 – 7.17 (m, 3H), 3.41 (t, *J* = 6.6 Hz, 2H), 2.92 – 2.70 (m, 2H), 2.35 – 2.07 (m, 2H). **^{13}C NMR** (101 MHz, $CDCl_3$) δ 139.0, 128.8, 128.7, 127.0, 39.5, 33.1. Spectroscopic data obtained are consistent with the data reported in the literature.[16]

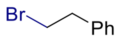

(3-bromopropyl)benzene (2m): Obtained following the GP, except that the reaction was performed in presence of 4-CzIPN (5 mol%, 20 mg). Colorless oil (57 mg, 57%); Obtained as a mixture of regioisomers: Anti-Markovnikov/Markovnikov 74:26 ratio. For the anti-Markovnikov product: **^{1}H NMR** (400 MHz, $CDCl_3$) δ 7.41 – 7.28 (m, 2H), 7.28 – 7.17 (m, 3H), 3.41 (t, *J* = 6.6 Hz, 2H), 2.92 – 2.70 (m, 2H), 2.35 – 2.07 (m, 2H). **^{13}C NMR** (101 MHz, $CDCl_3$) δ 140.7, 128.7, 128.6, 126.3, 34.3, 34.1, 33.2. Spectroscopic data obtained are consistent with the data reported in the literature.[12] For the Markovnikov product: **^{1}H NMR** (400 MHz, $CDCl_3$) δ 7.41 – 7.28 (m, 2H), 7.28 – 7.17 (m, 3H), 4.37 – 4.27 (m, 1H), 3.25 (dd, *J* = 14.0, 7.0 Hz, 1H), 3.09 (dd, *J* = 14.0, 7.0 Hz, 1H), 2.92 – 2.70 (m, 1H), 1.71 (d, *J* = 6.7 Hz, 3H). ^{13}C NMR (101 MHz, $CDCl_3$) δ 138.6, 129.3, 127.0, 126.3, 50.7, 47.6, 25.8. Spectroscopic data obtained are consistent with the data reported in the literature.[12]

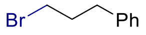

1,5-dibromopentane (2n): Obtained following the GP, except that the reaction was performed in presence of 4-CzIPN (5 mol%, 20 mg). Colorless oil (50 mg, 43%); **^{1}H NMR** (400 MHz, $CDCl_3$) δ 3.52 – 3.23 (m, 4H), 2.19 – 1.68 (m, 4H), 1.74 – 1.34 (m, 4H). **^{13}C NMR** (101 MHz, $CDCl_3$) δ 33.4, 31.9, 26.9. Spectroscopic data obtained are consistent with the data reported in the literature.[17]

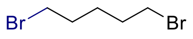

1-bromo-3-((5-bromopentyl)oxo)benzene (2o): Obtained following the GP, except that the reaction was performed in presence of 4-CzIPN (5 mol%, 20 mg) and DBE was used as solvent. Colorless oil (140 mg, 87%); **^{1}H NMR** (400 MHz, $CDCl_3$) δ 7.17 – 7.10 (m, 1H), 7.10 – 7.02 (m, 2H), 6.82 (ddd, *J* = 8.2, 2.4, 1.1 Hz, 1H), 3.94 (t, *J* = 6.8 Hz, 2H), 3.43 (t, *J* = 6.8 Hz, 2H), 2.00 – 1.87 (m, 2H), 1.86 – 1.74 (m, 2H), 1.68 – 1.55 (m, 2H). **^{13}C NMR** (101 MHz, $CDCl_3$) δ 159.9, 130.6, 123.8, 122.9, 117.8, 113.6, 67.9, 33.6, 32.5, 28.4, 24.9. Spectroscopic data obtained are consistent with the data reported in the literature.[18]

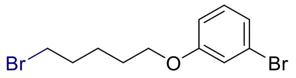

(2-bromovinyl)benzene (2p): Colorless oil (60 mg, 66%); Obtained as a mixture of stereoisomers in a *E*/*Z* = 80:20 ratio **^{1}H NMR** (400 MHz, $CDCl_3$) δ 7.71 – 7.67 (m, , 0.5H), 7.43 – 7.25 (m, 6H), 7.12 (d, *J* = 14.0 Hz, 1H), 7.08 (d, *J* = 8.1 Hz, 0.3H), 6.78 (d, *J* = 14.0 Hz, 1H), 6.44 (d, *J* = 8.1 Hz, 0.3H).**^{13}C NMR** (101 MHz, $CDCl_3$) δ 137.3, 136.1, 135.1, 132.5, 129.1, 128.9, 128.5, 128.4, 128.4, 126.2, 106.7, 106.5. Spectroscopic data obtained are consistent with the data reported in the literature.[19]

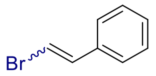

(2-bromovinyl)-3,5-dimethoxybenzene (2q): Colorless oil obtained as a mixture of stereoisomers in a

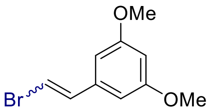

E/*Z* = 71:29 ratio (100 mg, 83%). **^{1}H NMR** (400 MHz, $CDCl_3$) δ 7.03 (d, *J* = 13.9 Hz, 1H), 7.00 – 6.99 (m, 0.3H), 6.86 (dd, *J* = 2.3, 0.6 Hz, 1H), 6.76 (d, *J* = 13.9 Hz, 1H), 6.46 (t, *J* = 2.3 Hz, 0.4H), 6.44 (dd, *J* = 2.3, 0.5 Hz, 2H), 6.43 – 6.39 (m, 1H), 3.81 (s, 3H), 3.79 (s, 6H). **^{13}C NMR** (101 MHz, $CDCl_3$) δ 161.1, 160.6, 137.8, 137.3, 136.7, 132.4, 107.2, 107.1, 106.8, 104.4, 100.8, 100.5, 55.5, 55.5. Spectroscopic data obtained are consistent with the data reported in the literature.[20]

(*Z*)-2-bromo-3-phenylprop-2-en-1-ol (2r): Melty brown solid (55 mg, 52%). **^{1}H NMR** (400 MHz, $CDCl_3$)

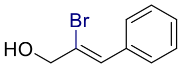

δ 7.66 – 7.57 (m, 2H), 7.45 – 7.29 (m, 3H), 7.09 (s, 1H), 4.42 (d, *J* = 1.2 Hz, 2H), 2.11 (s, 1H). **^{13}C NMR** (101 MHz, $CDCl_3$) δ 135.1, 129.1, 128.3, 128.0, 125.4, 69.5. Spectroscopic data obtained are consistent with the data reported in the literature.[21]

1-bromooct -1-ene (2s): Colorless oil obtained as as a mixture of stereoisomers in a *E*/*Z* = 63:37 ratio

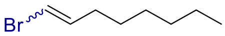

(35 mg, 37%). yellow oil (35 mg, 37%). **^{1}H NMR** (400 MHz, $CDCl_3$) δ 6.21 – 5.98 (m, 2H), 2.19 (ddd, *J* = 14.4, 6.9, 1.2 Hz, 1.3H), 2.03 (ddd, *J* = 14.7, 7.3, 1.4 Hz, 0.7H), 1.46 – 1.23 (m, 8H), 0.93 – 0.84 (m, 3H).**^{13}C NMR** (101 MHz, $CDCl_3$) δ 138.4, 135.2, 107.7, 104.1, 33.1, 31.8, 31.7, 29.8, 29.0, 28.8, 28.7, 28.3, 22.7, 22.7, 14.2, 14.2. Spectroscopic data obtained are consistent with the data reported in the literature.[22]

1-bromo-5-chloropent-1-ene (2t): The product was obtained in 92% NMR yield. Colorless oil obtained

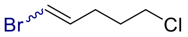

as a mixture of stereoisomers in a *E*/*Z* = 72:28 ratio (55 mg, 60%). Note that the compound **2s** is highly volatile, thus it should not be dried under high vacuum and at room temperature. **^{1}H NMR** (400 MHz, $CDCl_3$) δ 6.37 – 5.94 (m, 2H), 3.62 – 3.55 (m, 2H), 2.44 – 2.36 (m, 1.5H), 2.30 – 2.23 (m, 0.6H), 2.03 – 1.81 (m, 2H). **^{13}C NMR** (101 MHz, $CDCl_3$) δ 136.2, 133.2, 109.3, 105.9, 44.3, 44.0, 31.3, 31.2, 30.1, 27.3. Spectroscopic data obtained are consistent with the data reported in the literature.[23]

(4-bromobut-3-en-1-yl)benzene (2u): Colorless oil as a mixture of stereoisomers in a *E*/*Z* = 68:32 ratio

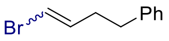

(97 mg, 92%). **^{1}H NMR** (400 MHz, $CDCl_3$) δ 7.34 – 7.28 (m, 2H), 7.25 – 7.16 (m, 3H), 6.27 – 6.03 (m, 3H), 2.78 – 2.70 (m, 2H), 2.58 – 2.51 (m, 1.3H), 2.41 – 2.34 (m, 0.7H). **^{13}C NMR** (101 MHz, $CDCl_3$) δ 141.3, 141.0, 137.2, 134.0, 128.6, 128.5, 128.5, 128.5, 126.3, 126.2, 108.5, 105.1, 35.1, 34.8, 34.3, 31.5. Spectroscopic data obtained are consistent with the data reported in the literature.[24]

dimethyl 2-bromomaleate (2v): Colorless oil obtained as a mixture of stereoisomers in a *E*/*Z* = 81:19

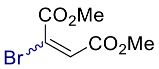

ratio (51 mg, 46%). **^{1}H NMR** (400 MHz, $CDCl_3$) δ 7.49 (s, 1H), 6.48 (s, 0.2H), 3.88 (s, 3H), 3.87 (s, 0.8H), 3.81 (s, 3H), 3.74 (s, 0.8H). **^{13}C NMR** (101 MHz, $CDCl_3$) δ 164.2, 164.0, 163.5, 162.3, 130.7, 127.2, 126.5, 125.1, 54.1, 53.5, 52.4, 52.4. Spectroscopic data obtained are consistent with the data reported in the literature.[25]

3-(2-bromo-2-phenylvinyl)oxazolidin-2-one (2w): Green solid (47 mg, 35%). Only one stereoisomer

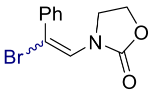

was obtained, the *Z*/*E*-configuration was not determined. **^{1}H NMR** (400 MHz, $CDCl_3$) δ 7.35 – 7.31 (m, 5H), 6.95 (s, 1H), 4.44 (t, J = 7.8 Hz, 2H), 3.73 (t, J = 7.8 Hz, 2H). **^{13}C NMR** (101 MHz, $CDCl_3$) δ 155.4, 135.6, 133.7, 128.9, 128.9, 116.5, 62.8, 45.3. **HRMS (ESI):** Calcd. for $C_{11}H_{11}BrNO_2$ [M+H^{+}, 100 %]: 267.9959, found 267.9968.

Methyl (3-bromopropanoyl)alaninate (2x): Obtained following the GP, except that the reaction was performed in presence of 4-CzIPN (5 mol%, 20 mg). Yellow pale solid (63 mg, 53%). **^{1}H NMR** (400 MHz, $CDCl_3$) δ 6.31 (s, 0H), 4.61 (p, *J* = 7.2 Hz, 1H), 3.75 (s, 3H), 3.67 – 3.48 (m, 2H), 2.91 – 2.63 (m, 2H), 1.41 (d, *J* = 7.1 Hz, 3H). **^{13}C NMR** (101 MHz, $CDCl_3$) δ 173.5, 169.3, 52.7, 48.3, 39.5, 27.2, 18.6. **HRMS (ESI):** Calcd. for $C_7H_{13}BrNO_3$ [M+H$^+$, 100 %]: 238.0073, found 238.0078.

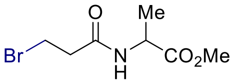

Methyl (3-bromopropanoyl)phenylalanyalaninate (2y): Obtained following the GP, except that the reaction was performed in presence of 4-CzIPN (5 mol%, 20 mg). Yellow pale solid (110 mg, 58%). **^{1}H NMR** (400 MHz, $CDCl_3$) δ 7.34 – 7.19 (m, 5H), 7.02 (d, *J* = 8.0 Hz, 1H), 6.96 (d, *J* = 7.2 Hz, 1H), 4.86 (q, *J* = 7.2 Hz, 1H), 4.52 (t, *J* = 7.2 Hz, 1H), 3.75 (s, 3H), 3.58 (t, *J* = 6.6 Hz, 2H), 3.09 (qd, *J* = 13.8, 6.9 Hz, 2H), 2.78 (t, *J* = 6.6 Hz, 2H), 1.38 (d, *J* = 7.2 Hz, 3H). **^{13}C NMR** (101 MHz, $CDCl_3$) δ 172.8, 170.9, 169.9, 136.5, 129.5, 128.7, 127.1, 54.5, 52.6, 48.3, 39.4, 38.6, 27.3, 18.1. **HRMS (ESI):** Calcd. for $C_{16}H_{22}BrN_2O_3$ [M+H$^+$, 100 %]: 385.0757, found 385.0759.

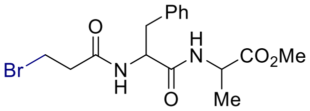

(8*R*,9*S*,13*S*,14*S*)-13-methyl-17-oxo-7,8,9,11,12,13,14,15,16,17-decahydro-6*H*-cyclopenta[*a*]phenanthren-3-yl 3-bromopropanoate (2z): Obtained following the GP, except that the reaction mixture was purified by washing several time with a mixture of 1/1 Et_2O/pentane, giving **2z** in pure form. White solid (152 mg, 75%). **^{1}H NMR** (400 MHz, $CDCl_3$) δ 7.29 (dd, *J* = 8.6, 1.1 Hz, 1H), 6.87 (dd, *J* = 8.4, 2.6 Hz, 1H), 6.83 (d, *J* = 2.4 Hz, 1H), 3.67 (t, *J* = 6.8 Hz, 2H), 3.15 (t, *J* = 6.8 Hz, 2H), 2.91 (dd, *J* = 8.7, 3.9 Hz, 2H), 2.63 – 2.45 (m, 1H), 2.41 – 2.37 (m, 1H), 2.32 – 2.23 (m, 1H), 2.20 – 1.90 (m, 4H), 1.70 – 1.36 (m, 6H), 0.90 (s, 3H). **^{13}C NMR** (101 MHz, $CDCl_3$) δ 220.8, 169.4, 148.3, 138.2, 137.7, 126.5, 121.5, 118.7, 50.4, 48.0, 44.2, 38.0, 37.9, 35.9, 31.6, 29.4, 26.4, 25.8, 25.8, 21.6, 13.9. **HRMS (ESI):** Calcd. for $C_{21}H_{26}BrO_3$ [M+H$^+$, 100 %]: 405.1060, found 405.1054.

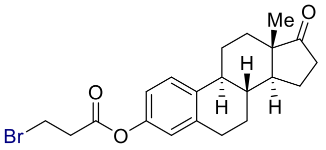

3-bromopropyl 2-(4-isobutylphenyl)propanoate (2aa): Obtained following the GP, except that the reaction was performed in presence of 4-CzIPN (5 mol%, 20 mg) and DBE was used as solvent. Yellow pale oil (73 mg, 46%). **^{1}H NMR** (400 MHz, $CDCl_3$) δ 7.25 – 7.15 (m, 2H), 7.14 – 7.06 (m, 2H), 4.28 – 4.12 (m, 2H), 3.70 (q, *J* = 7.2 Hz, 1H), 3.28 (td, *J* = 6.6, 0.7 Hz, 2H), 2.45 (d, *J* = 7.2 Hz, 2H), 2.10 (tt, *J* = 6.6, 6.0 Hz, 2H), 1.85 (dtd, *J* = 13.3, 6.7, 0.6 Hz, 1H), 1.50 (d, *J* = 7.2 Hz, 3H), 0.90 (d, *J* = 6.6 Hz, 6H). **^{13}C NMR** (101 MHz, $CDCl_3$) δ 174.7, 140.8, 137.8, 129.5, 127.2, 62.4, 45.2, 45.1, 31.7, 30.3, 29.4, 22.5, 18.4. **HRMS (ESI):** Calcd. for $C_{16}H_{24}BrO_2$ [M+H$^+$, 100 %]: 327.0960, found 327.0953

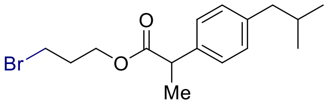

4-cyanophenyl 3-iodopropanoate (3a): Slightly yellow oil (126 mg, 84%). **^{1}H NMR** (400 MHz, $CDCl_3$) δ 7.72 (d, *J* = 8.9 Hz, 2H), 7.30 (d, *J* = 8.9 Hz, 2H), 3.45 (td, *J* = 6.8, 0.7 Hz, 2H), 3.30 (td, *J* = 6.9, 0.7 Hz, 2H). **^{13}C NMR** (101 MHz, $CDCl_3$) δ 168.9, 153.7, 133.8, 122.8, 118.2, 110.1, 38.5, -4.7. **HRMS (ESI):** Calcd. for $C_{10}H_9INO_2$ [M+H$^+$, 100 %]: 301.9673, found 301.9669.

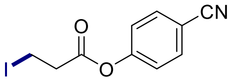

Methyl 4-(((3-iodopropanoyl)oxy)methyl)benzoate (3b): colorless oil (110 mg, 63%). **^{1}H NMR** (400 MHz, $CDCl_3$) δ 8.05 – 8.00 (m, 2H), 7.44 – 7.39 (m, 2H), 5.19 (s, 2H), 3.90 (s, 3H), 3.33 (t, *J* = 7.0 Hz, 2H), 3.04 (t, *J* = 7.0 Hz, 2H). **^{13}C NMR** (101 MHz, $CDCl_3$) δ 170.9, 166.7, 140.6, 130.1, 129.9, 127.9, 66.0, 52.3, 38.6, -4.1. **HRMS (ESI):** Calcd. for $C_{12}H_{13}IO_4$ [M+H$^+$, 100 %]: 348.9938, found 348.9931.

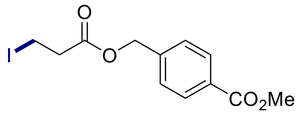

3-iodo-*N*-methyl-*N*-phenylpropanamide (3c): Obtained following the GP, except that the reaction was performed in presence of 4-CzIPN (5 mol%, 20 mg) and the purification was carried out with DCM/ethyl acetate eluent mixture. Slightly yellow oil (41 mg, 29%). **^{1}H NMR** (400 MHz, CDCl3) δ 7.48 – 7.39 (m, 2H), 7.39 – 7.32 (m, 1H), 7.21 – 7.14 (m, 2H), 3.32 (t, J = 7.0 Hz, 2H), 3.28 (s, 3H), 2.69 (t, J = 7.0 Hz, 2H). **^{13}C NMR** (101 MHz, $CDCl_3$) δ 170.6, 143.4, 130.1, 128.3, 127.5, 38.2, 37.6. **HRMS (ESI):** Calcd. for $C_{10}H_{13}INO$ [M+H$^+$, 100 %]: 290.0036, found 290.0028.

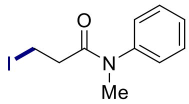

Methyl (3-iodopropanoyl)alaninate (3d): Obtained following the GP, except that the reaction was performed in presence of 4-CzIPN (5 mol%, 20 mg), and DCM was used as solvent. Orange solid (110 mg, 77%). **^{1}H NMR** (400 MHz, $CDCl_3$) δ 6.30 (d, *J* = 6.9 Hz, 1H), 4.60 (p, *J* = 7.2 Hz, 1H), 3.74 (s, 3H), 3.43 – 3.28 (m, 2H), 2.92 – 2.74 (m, 2H), 1.41 (d, *J* = 7.2 Hz, 3H). **^{13}C NMR** (101 MHz, $CDCl_3$) δ 173.5, 170.0, 52.7, 48.3, 40.4, 18.6, -2.1. **HRMS (ESI):** Calcd. for $C_7H_{13}INO_3$ [M+H$^+$, 100 %]: 285.9940, found 285.9943.

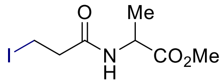

4-cyanophenyl 5-chloropentanoate (5): Pale yellow solid (63 mg, 50%). **^{1}H NMR** (400 MHz, $CDCl_3$) δ 7.68 (d, *J* = 8.9 Hz, 2H), 7.23 (d, *J* = 9.0 Hz, 2H), 3.59 (t, *J* = 6.1 Hz, 2H), 2.64 (t, *J* = 7.1 Hz, 2H), 1.97 – 1.83 (m, 4H). **^{13}C NMR** (101 MHz, $CDCl_3$) δ 170.9, 154.0, 133.8, 122.8, 118.3, 109.9, 44.4, 33.6, 31.8, 22.1. **HRMS (ESI):** Calcd. for $C_{12}H_{13}ClNO_2$ [M+H$^+$, 100 %]: 238.0629, found 238.0630.

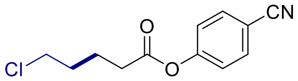

D. Mechanistic investigations

Experiment in the dark.

In a vial wrapped with aluminum foil was added DBE (130 µL, 1.5 mmol, 5.0 equiv.) in AcOEt (0.3 mL). The solution was left in the dark for 10-15 minutes before adding ethyl acrylate **1a** (0.3 mmol, 1.0 equiv.) and TTMSS (110 µL, 0.36 mmol, 1.2 equiv.). Then, the reaction mixture was degassed via a freeze pump thaw procedure and stirred in the dark for 18 h. NMR analysis showed that no product **2a** was formed in those conditions.

Deuterium labeling experiments.

In DCM-*d*₂. For determining the hydrogen source in this reaction, a deuterium labeling experiment in DCM-d_2 was carried out. The DCM-d_2 was selected as solvent for this experiment instead of AcOEt-d_8 for economic reasons. Deuterium incorporation was determined by ^{1}H NMR analysis. NMR analyses showed that no deuterium was incorporated into compound **2a**, suggesting that the solvent is unlikely the hydrogen source in this process. ^{1}H NMR yield of **2a** = 83%.

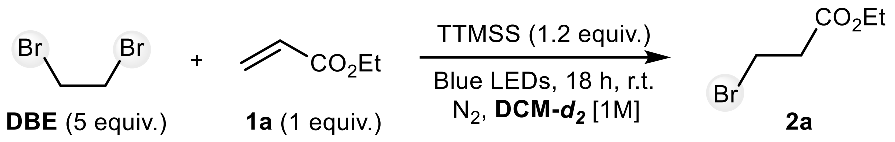

Figure S2. *Reaction performed in* ***DCM***-d₂.

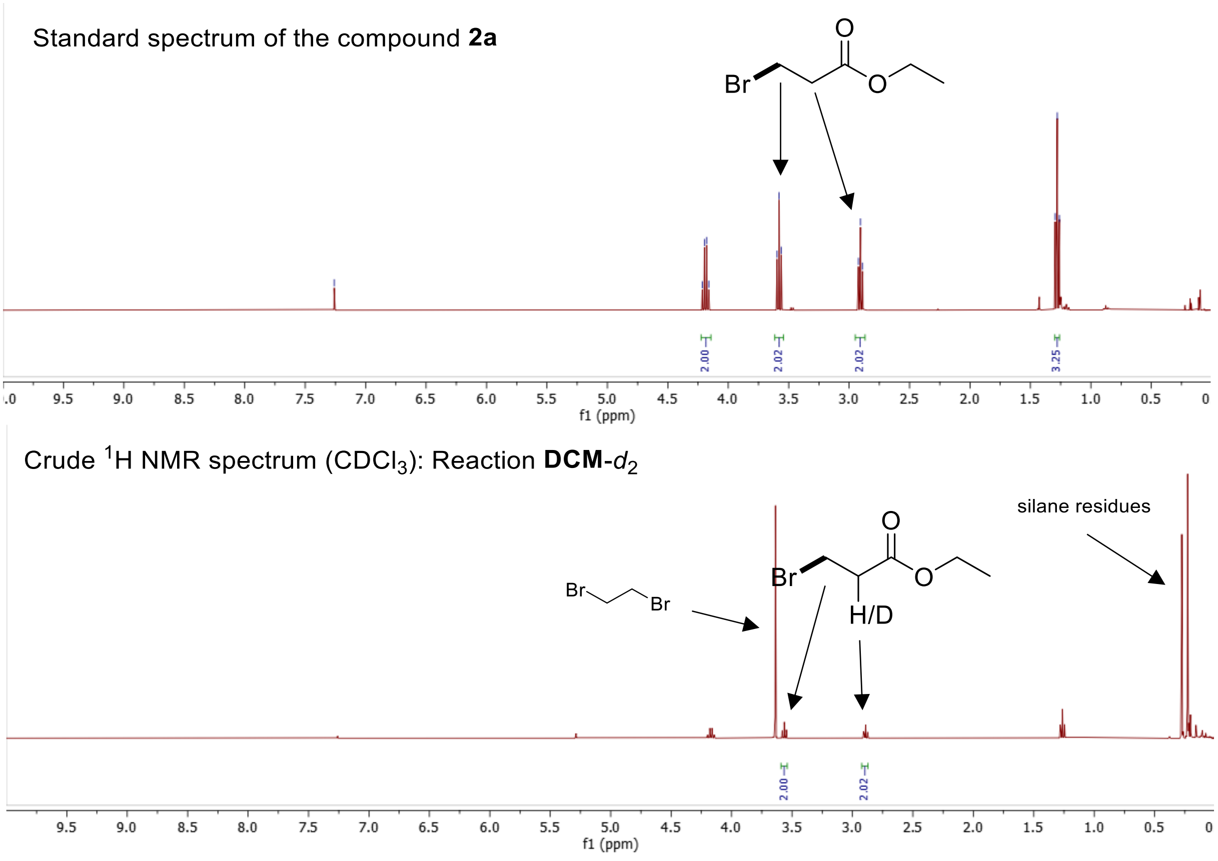

Figure S3*. Determination of deuterium incorporation for the reaction performed in* ***DCM***-d₂

With $(TMS)_3Si$-D. The deuterated supersilane $(TMS)_3SiD$ was prepared according to a literature procedure.[26] Deuterium incorporation was determined by ^1H NMR analysis. The latter showed that 84% of deuterium was incorporated into compound **2a**, supporting the proposition that the supersilane is the hydrogen source in the reaction. ^1H NMR yield of **2a** + **2a**-d_1= quant.

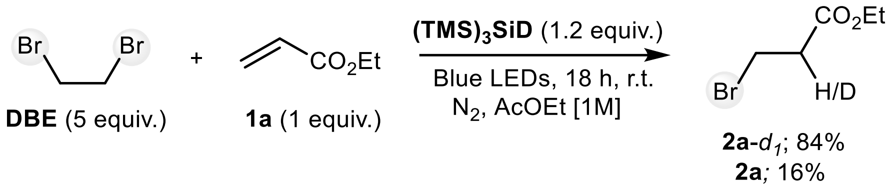

***Figure S4.** Reaction performed with **deuterated TTMSS**.*

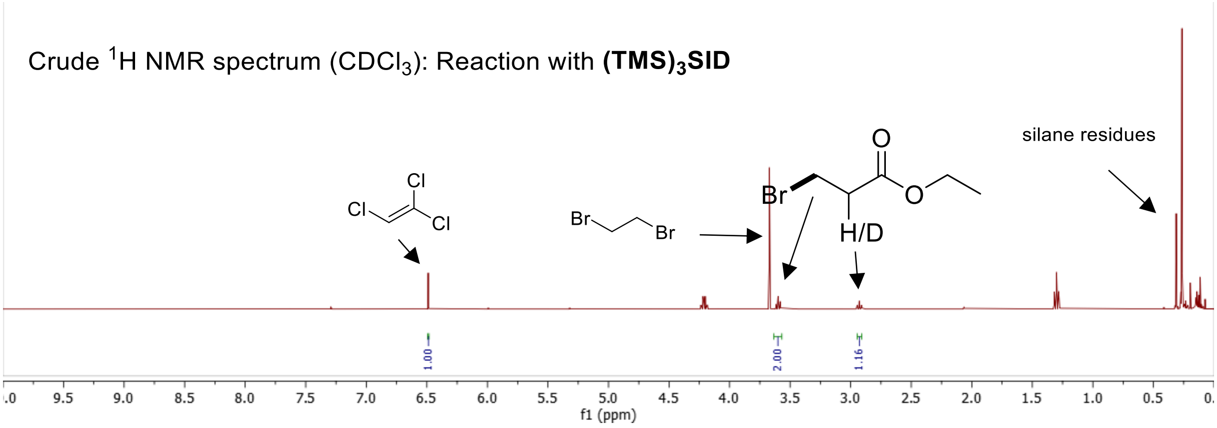

***Figure S5**. Determination of deuterium incorporation for the reaction performed with **deuterated supersilane**.*

Radical trapping experiment

The reaction was set up following the GP, except that TEMPO (5.0 equiv.) was added. MS and NMR analyses showed that the reaction was completely inhibited in presence of TEMPO, thus the product **2a** was not formed. Moreover, the TEMPO-$Si(TMS)_3$ and TEMPO-CH_2CH_2Br adducts were detected by MS. Unfortunately, the formation of the TEMPO-Br adduct was not observed in this experiment.

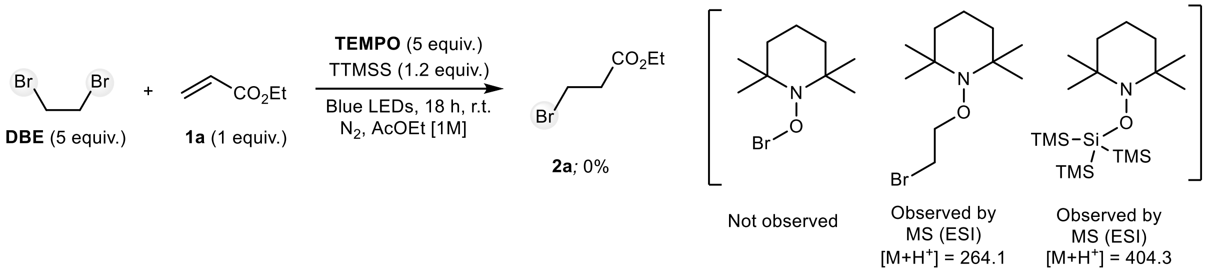

Figure S6. Reaction performed in presence of TEMPO.

Experiment using classical initiators

In a vial wrapped with aluminum foil was added DBE (130 µL, 1.5 mmol, 5.0 equiv.) in AcOEt (0.3 mL). The solution was left in the dark for 10-15 minutes before adding ethyl acrylate **1a** (0.3 mmol, 1.0 equiv.), TTMSS (110 µL, 0.36 mmol, 1.2 equiv.), and the appropriate initiator. Then, the reaction mixture was degassed via a freeze pump thaw procedure and stirred in the dark for 18 h at the appropriate temperature. Results obtained are listed in the **Figure S7**, yields refer to NMR yields.

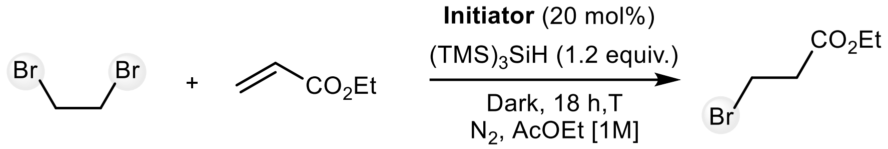

DBE (5 equiv.) **1a** (1 equiv.) **2a**

$(BzO)_2$, 50°C	21%
$(BzO)_2$, 80°C	quant.
AIBN, 50°C	quant.
AIBN,80°C	quant.

Figure S7. *Results obtained using classical initiators*

E. References

[1] Chanthamath, S.; Takaki, S.; Shibatomi, K.; Iwasa, S. *Angew. Chem. Int. Ed Engl.* **2013**, *52*, 5818–5821.
[2] Kokosza, K.; Balzarini, J.; Piotrowska, D. G. *Bioorg. Med. Chem.* **2013**, *21*, 1097–1108.
[3] Kwarcinski, F. E.; Steffey, M. E.; Fox, C. C.; Soellner, M. B. *ACS Med. Chem. Lett.* **2015**, 6, 898–901.
[4] Klussmann, M.; Boess, E.; Karanestora, S.; Bosnidou, A.-E.; Schweitzer-Chaput, B.; Hasenbeck, M. *Synlett* **2015**, *26*, 1973–1976.
[5] Liu, Q.; Zhu, F.-P.; Jin, X.-L.; Wang, X.-J.; Chen, H.; Wu, L.-Z. *Chem. Eur. J.* **2015**, *21*, 10326–10329.
[6] Bugarin, A.; Jones, K. D.; Connell, B. T. *Chem. Commun.* **2010**, *46*, 1715–1717.
[7] Della-Felice, F.; Zanini, M.; Jie, X.; Tan, E.; Echavarren, A. M. *Angew. Chem. Int. Ed Engl.* **2021**, *60* (11), 5693–5698.
[8] Hamada, T.; Ye, X.; Stahl, S. S. *J. Am. Chem. Soc*. **2008**, *130*, 833–835.
[9] Streuff, J.; Nieger, M.; Muñiz, K. *Chem. Eur. J.* **2006**, *12*, 4362–4371.
[10] Hell, S. M.; Meyer, C. F.; Ortalli, S.; Sap, J. B. I.; Chen, X.; Gouverneur, V. *Chem. Sci.* **2021**, *12*, 12149–12155.
[11] Li, N.; Gui, Y.; Chu, M.; You, M.; Qiu, X.; Liu, H.; Wang, S.; Deng, M.; Ji, B. *Org. Lett.* **2021**, *23* (21), 8460–8464.
[12] Cruz, D. A.; Sinka, V.; de Armas, P.; Steingruber, H. S.; Fernández, I.; Martín, V. S.; Miranda, P. O.; Padrón, J. I. *Org. Lett.* **2021**, *23*, 6105–6109.
[13] L. Pasquinucci, O. Prezzavento A. Marrazzo, E. Amata, S. Ronsisvalle, Z. Georgoussi, D.-D. Fourla, G. M. Scoto, C. Parenti, G. Aricò, G. Ronsisvalle. *Bioorg. Med. Chem.* **2010**, 4975.
[14] Murakami, T.; Furusawa, K. *Synthesis* **2002**, 479–482
[15] M.V. Barysevich, Y. M. Aniskevic, A. L. Hurski. *Synlett*. **2021**, 32, 1934-1938.
[16] Cahiez, G.; Gager, O.; Moyeux, A.; Delacroix, T. *Adv. Synth. Catal.* **2012**, *354*, 1519–1528.
[17] Galli, M.; Fletcher, C. J.; del Pozo, M.; Goldup, S. M. *Org. Biomol. Chem*. **2016**, *14*, 5622–5626.
[18] L. Salernoa, V. Pittalà, G. Romeo, M. N. Modica, A. Marrazzo, M. A. Siracusa, V. Sorrenti, C. Di Giacomo, L. Vanella, N. N. Parayath, K. Greish *Eur. J. Med. Chem.* **2015**, *96*, 162–172.
[19] F. Wei, H. Li, C. Song, Y. Ma, L. Zhou, C.-H. Tung, Z. Xu. *Org. Lett.* **2015**, *17*, 2860–2863.
[20] J. P. Burke, M. Sabat, D. A. Iovan, W. H. Myers, J. J. Chruma. *Org. Lett.* **2010**, *14*, 3192–3195.
[21] H. Weischedel, D. Schmidt, J. Conrad, U. Beifuss. *Tetrahedron* **2018**, *74*, 6426–6441.
[22] Birepinte, M.; Liautard, V.; Chabaud, L.; Pucheault, M. *Org. Lett.* **2020**, *22*, 2838–2843.
[23] Brown, H. C.; Larock, R. C.; Gupta, S. K.; Rajagopalan, S.; Bhat, N. G. *J. Org. Chem.* **1989**, *54*, 6079–6084.
[24] G. Jones, M. E. Fantina, A. H. Pachtman. *J. Org. Chem.* **1976**, *41*, 329–333.
[25] G. Jones, M. E. Fantina, A. H. Pachtman. *J. Org. Chem.* **1976**, *41*, 329–333.
[26] Pickford, H. D.; Nugent, J.; Owen, B.; Mousseau, J. J.; Smith, R. C.; Anderson, E. A. *J. Am. Chem. Soc.* **2021**, *143*, 9729–9736.

F. NMR Spectra of compounds 1, 2, 3, 4, 5

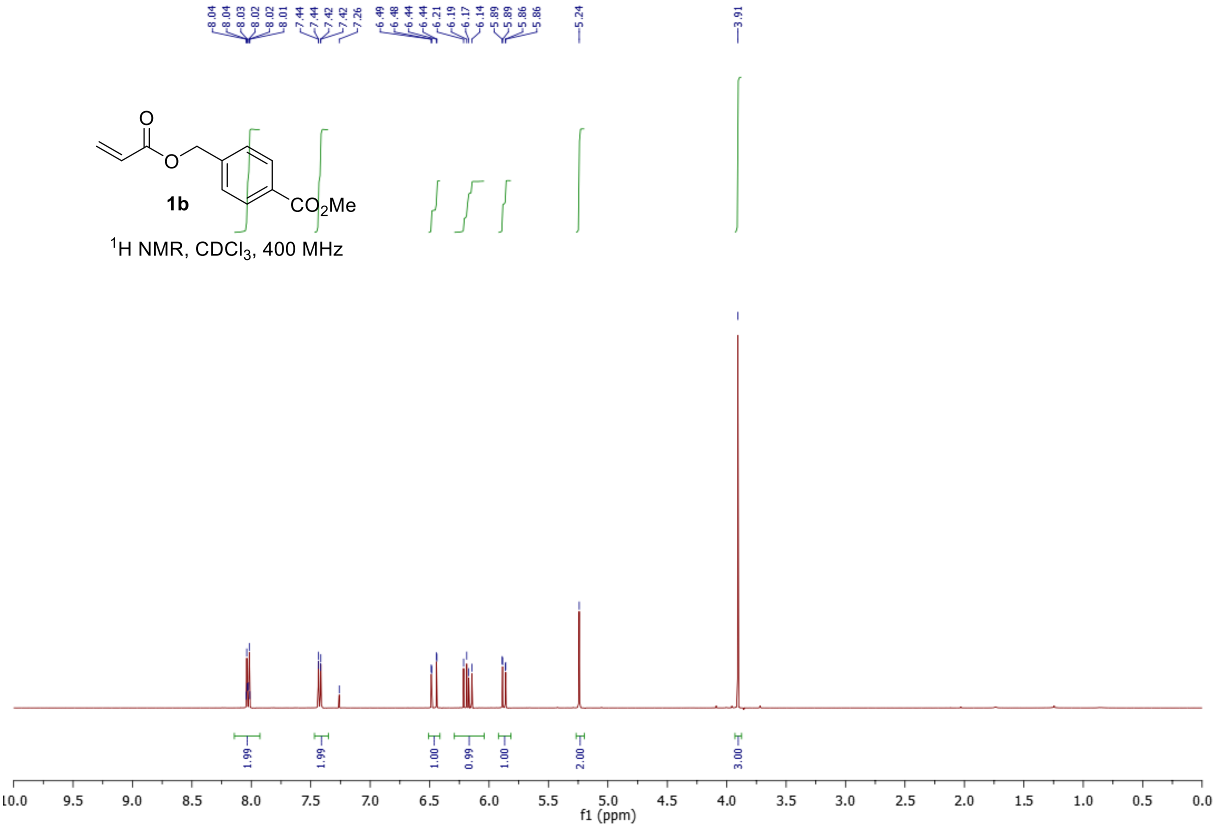

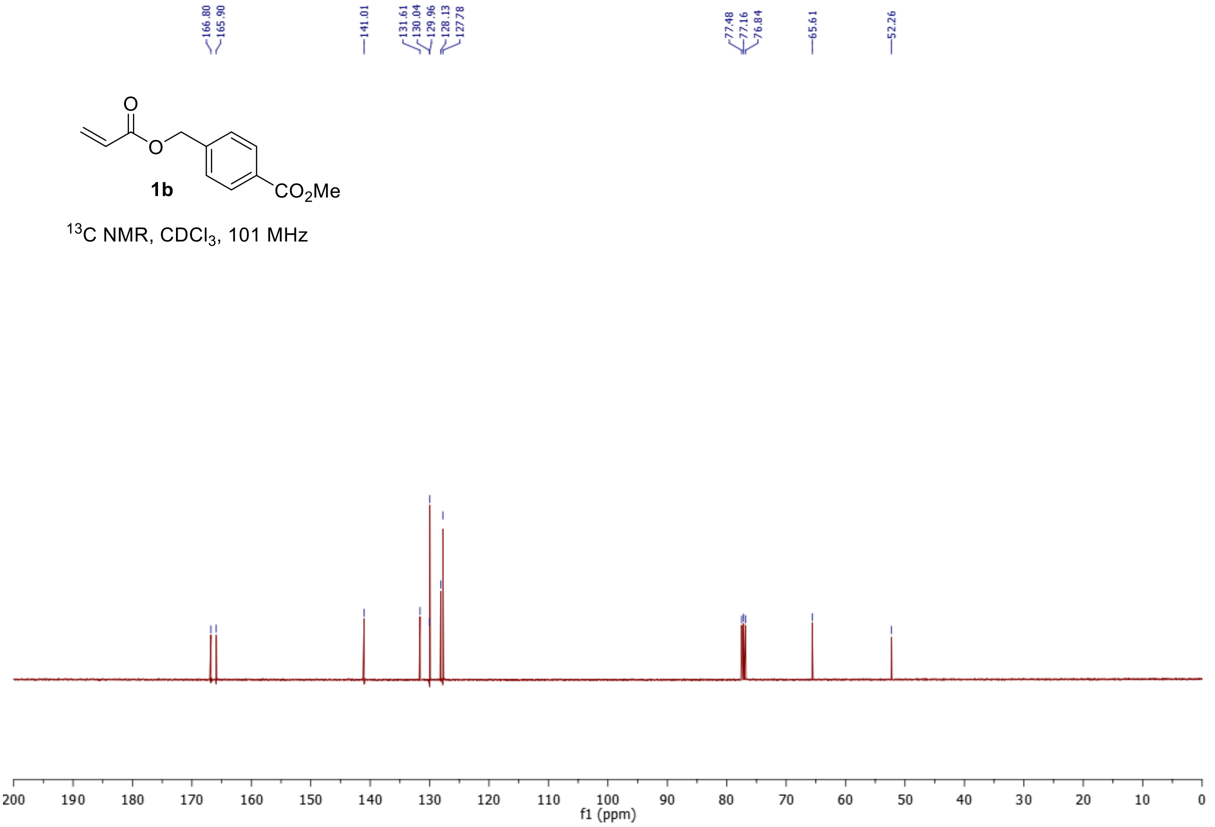

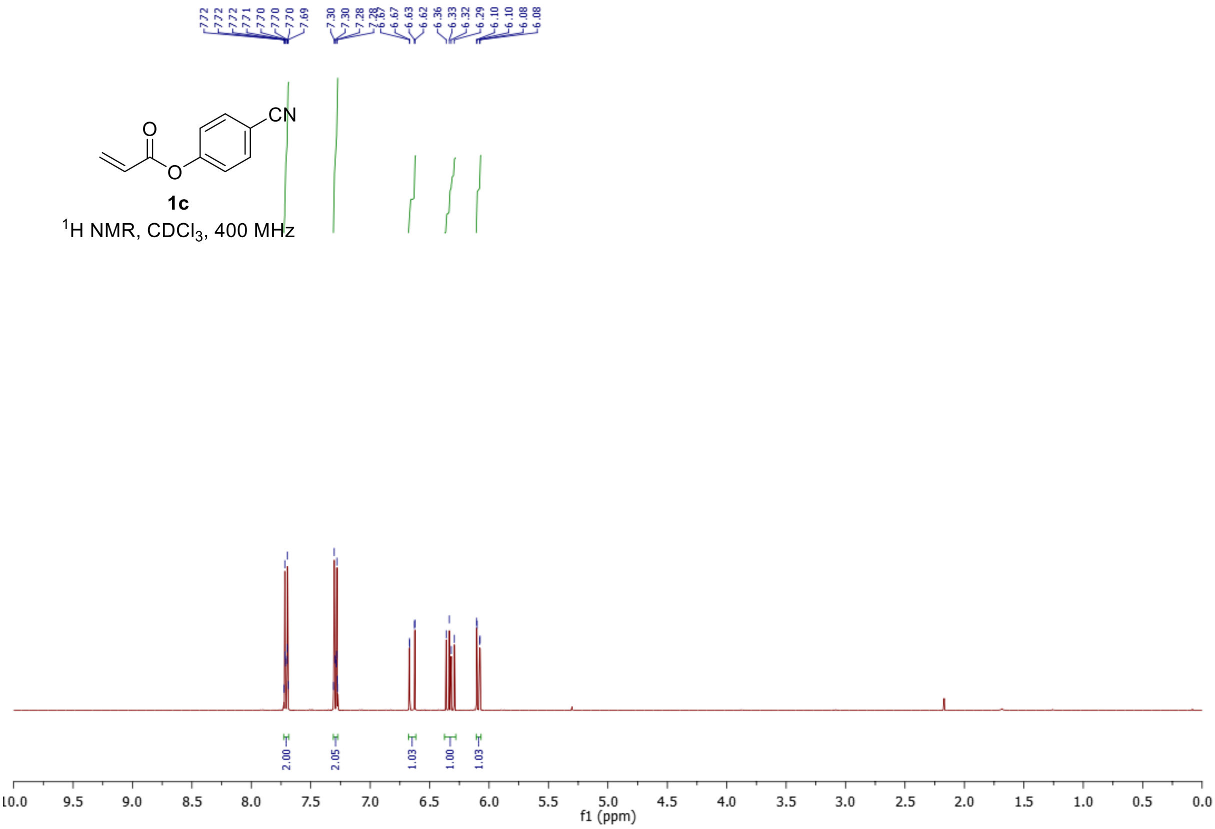
7.72
7.72
7.72
7.71
7.70
7.70
7.70
7.69
7.30
7.30
7.28
7.28
6.67
6.67
6.63
6.62
6.36
6.33
6.32
6.29
6.10
6.10
6.08
6.08
CN
O
O
1c
^{1}H NMR, $CDCl_3$, 400 MHz
2.00
2.05
1.03
1.00
1.03
f1 (ppm)

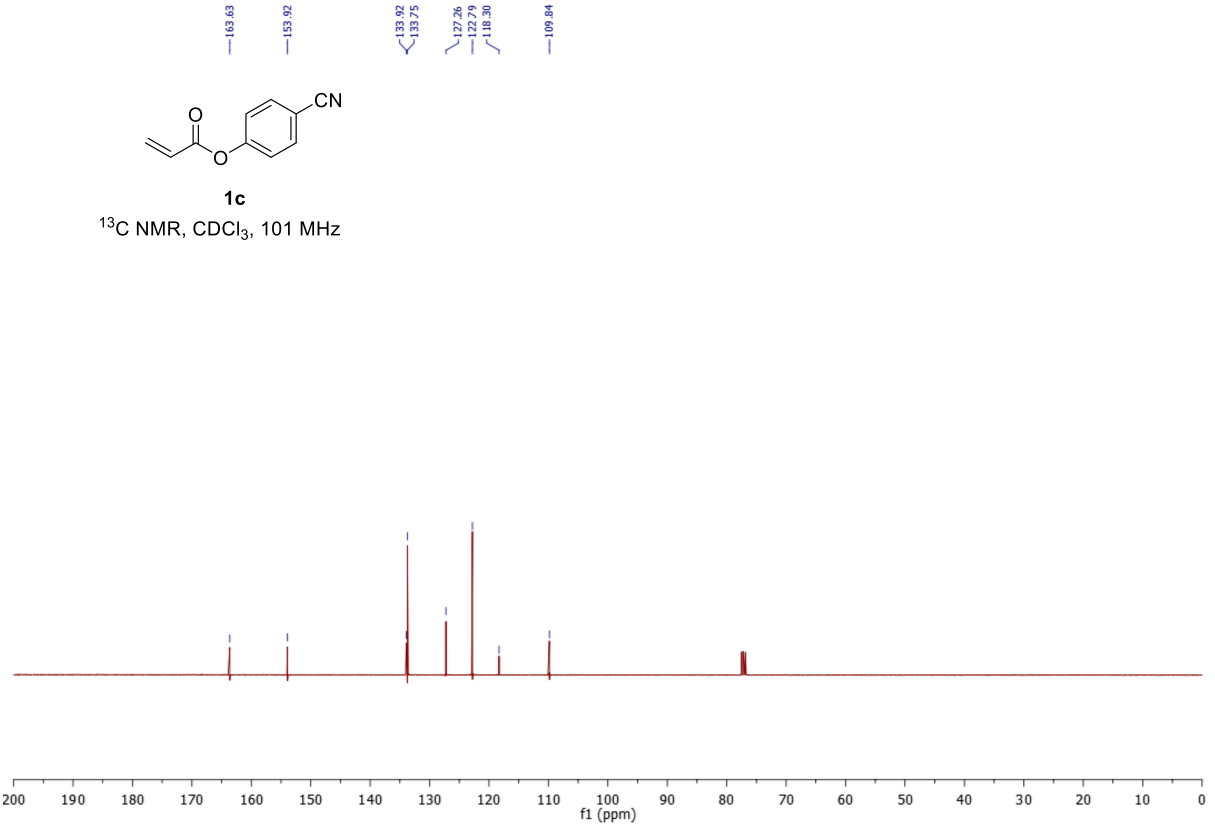
163.63
153.92
133.92
133.75
127.26
122.79
118.30
109.84
CN
O
O
1c
^{13}C NMR, $CDCl_3$, 101 MHz
f1 (ppm)

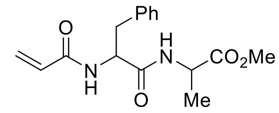

1y

^{1}H NMR, $CDCl_3$, 400 MHz

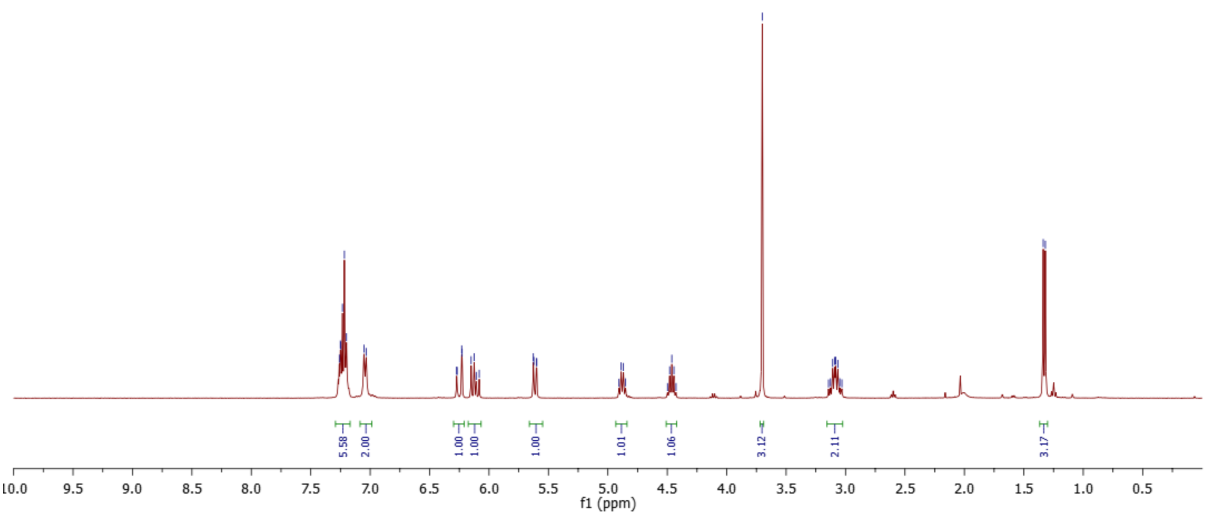

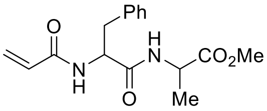

1y

^{13}C NMR, $CDCl_3$, 101 MHz

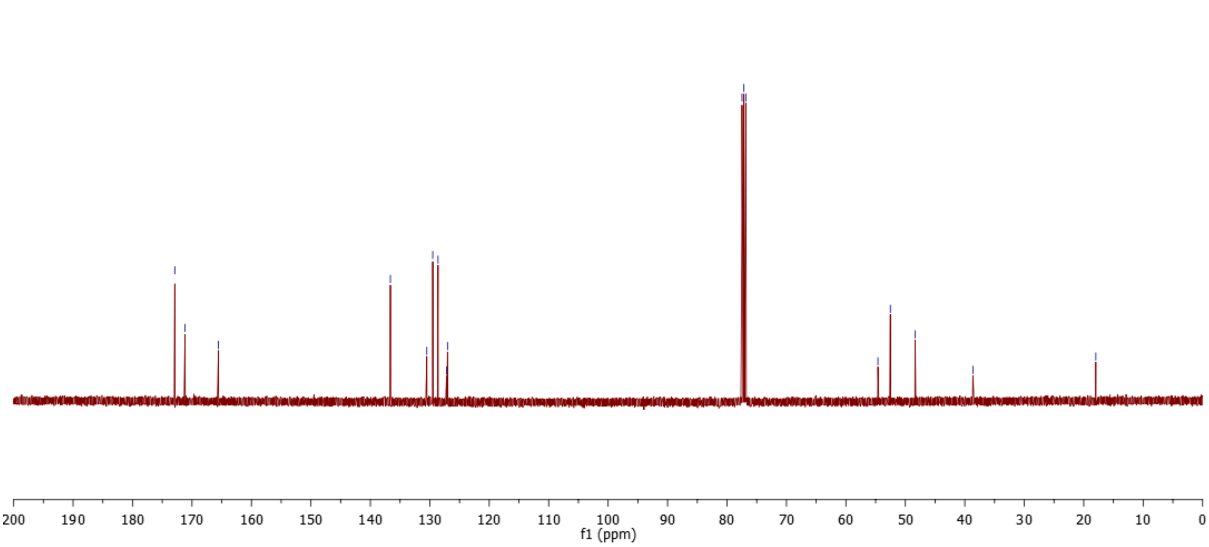

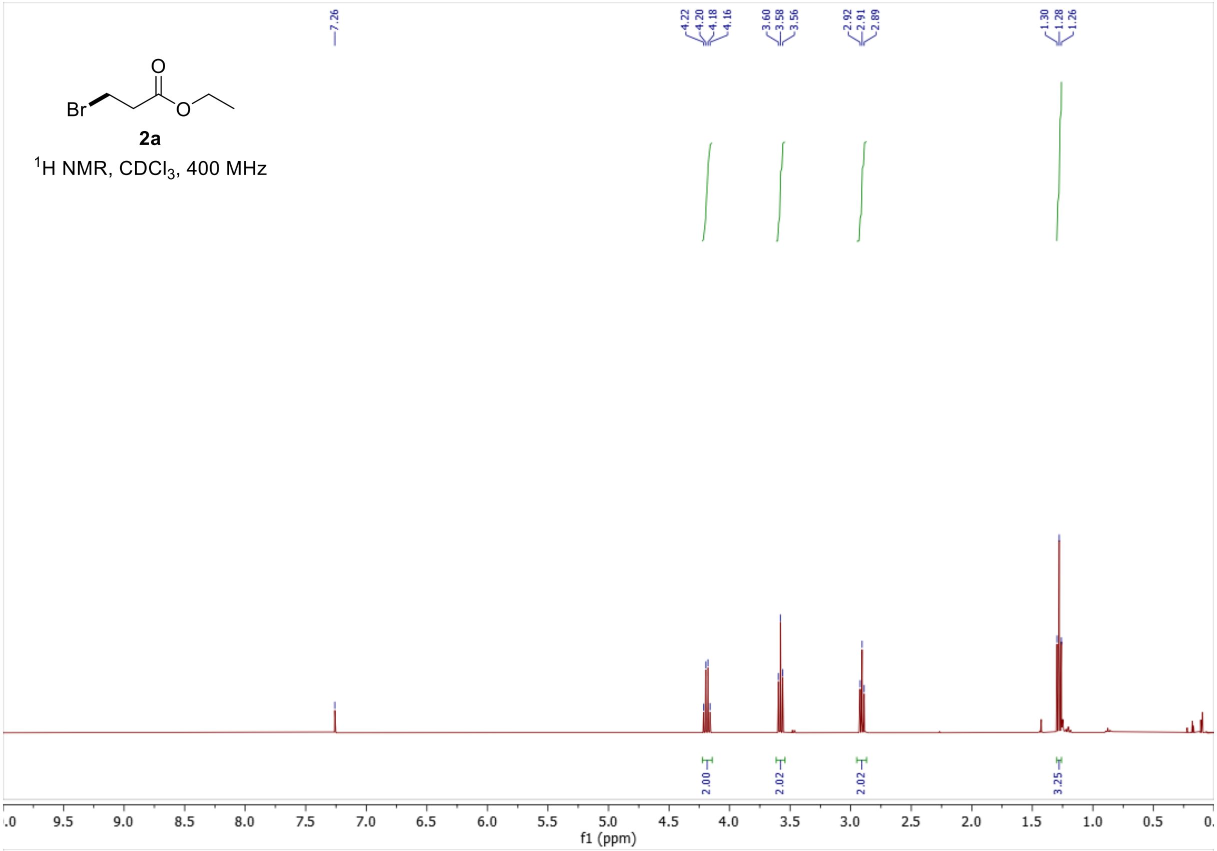
7.26
4.22
4.20
4.18
4.16
3.60
3.58
3.56
2.92
2.91
2.89
1.30
1.28
1.26
Br
O
O
2a
^{1}H NMR, CDCl$_3$, 400 MHz
2.00
2.02
2.02
3.25
f1 (ppm)

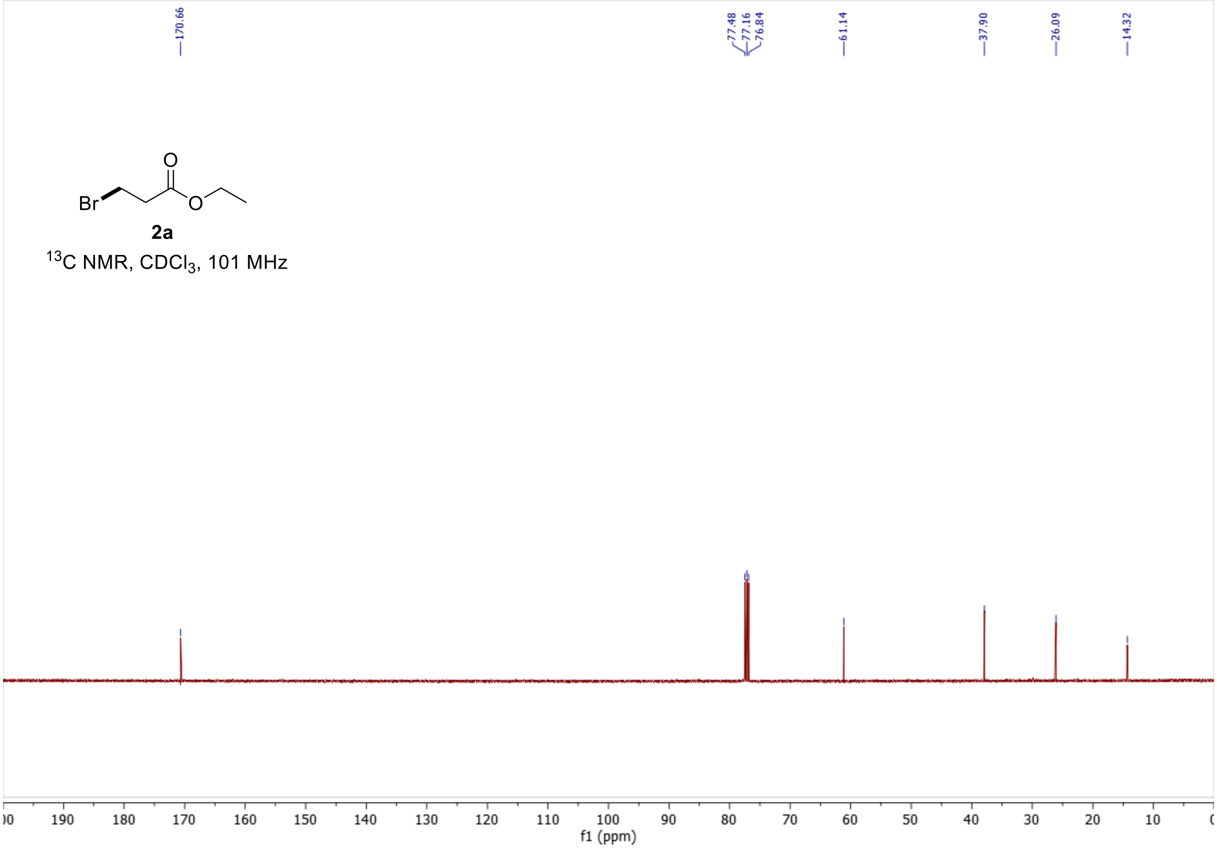
170.66
77.48
77.16
76.84
61.14
37.90
26.09
14.32
Br
O
O
2a
^{13}C NMR, CDCl$_3$, 101 MHz
f1 (ppm)

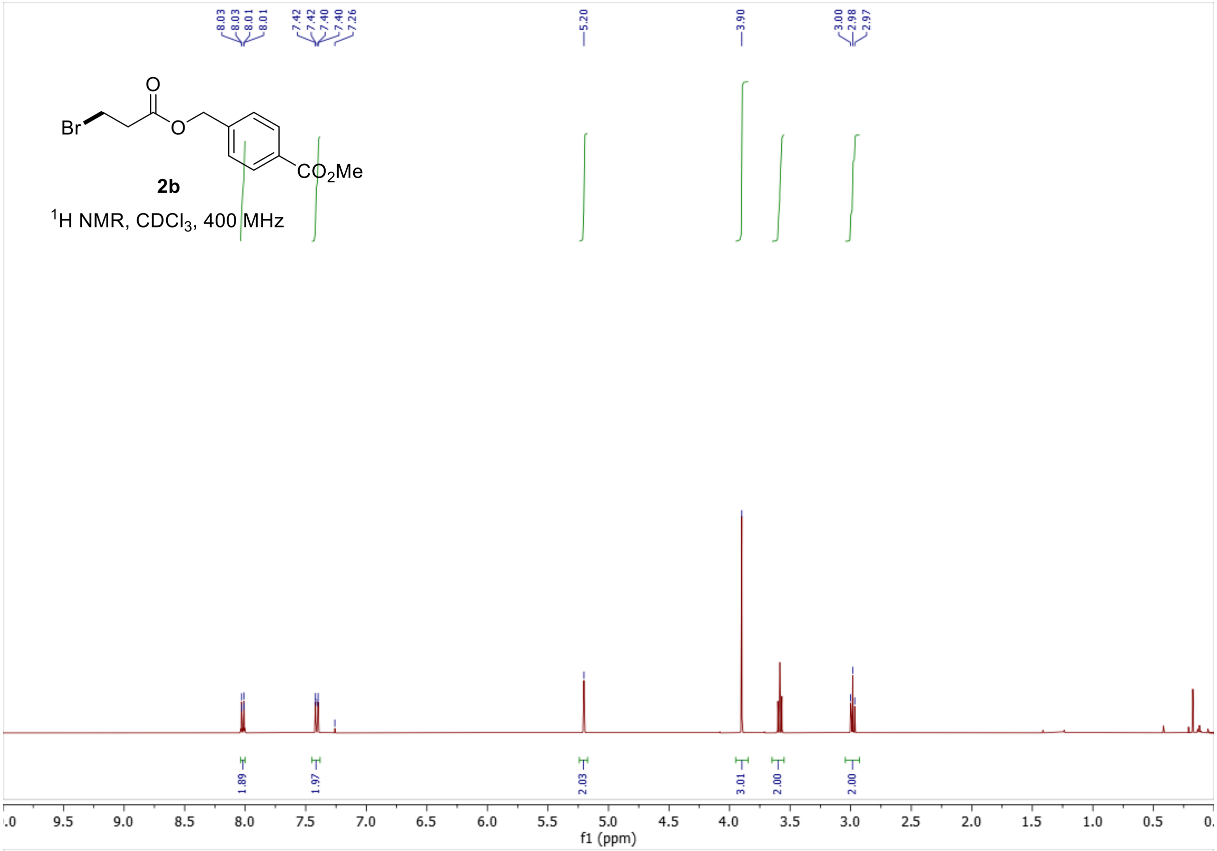
8.03
8.03
8.01
8.01
7.42
7.42
7.40
7.40
7.26
5.20
3.90
3.00
2.98
2.97
Br
O
O
CO_2Me
2b
^{1}H NMR, $CDCl_3$, 400 MHz
1.89
1.97
2.03
3.01
2.00
2.00
f1 (ppm)

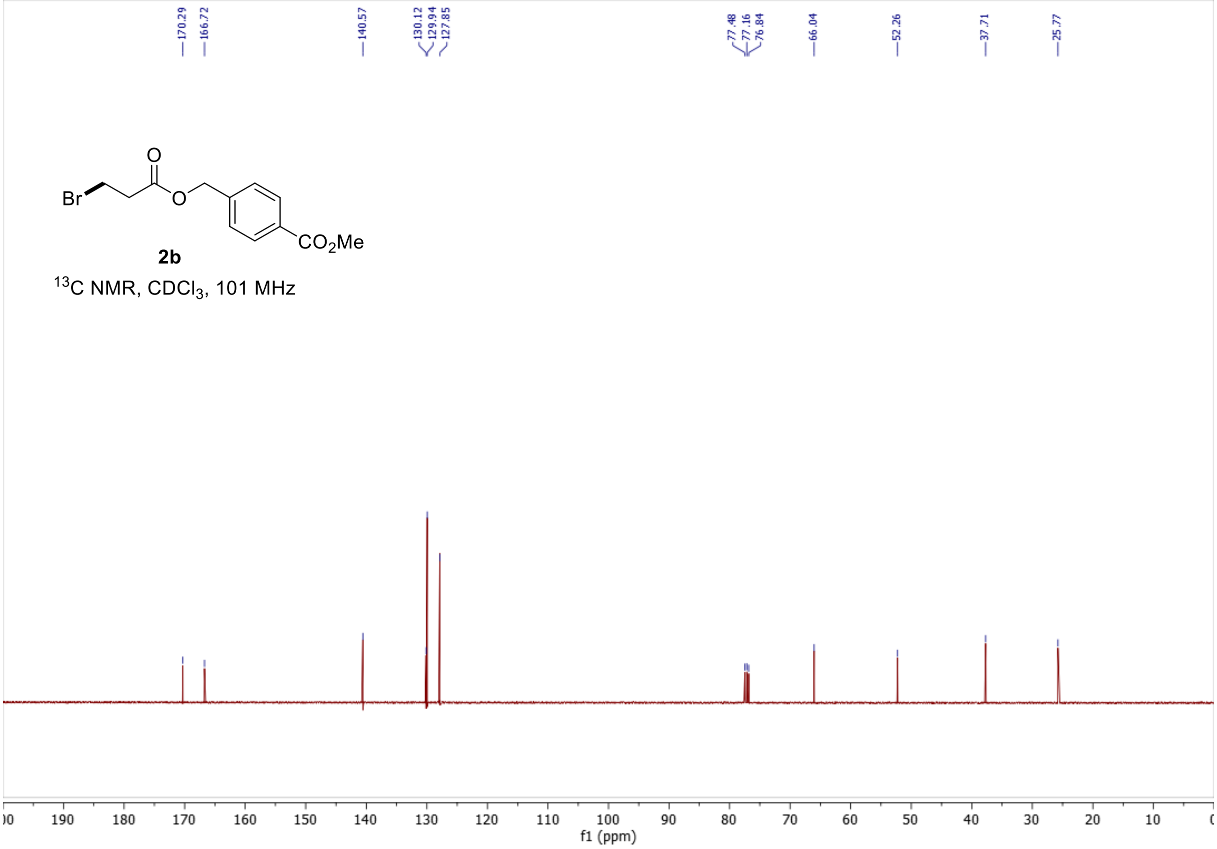
170.29
166.72
140.57
130.12
129.94
127.85
77.48
77.16
76.84
66.04
52.26
37.71
25.77
Br
O
O
CO_2Me
2b
^{13}C NMR, $CDCl_3$, 101 MHz
f1 (ppm)

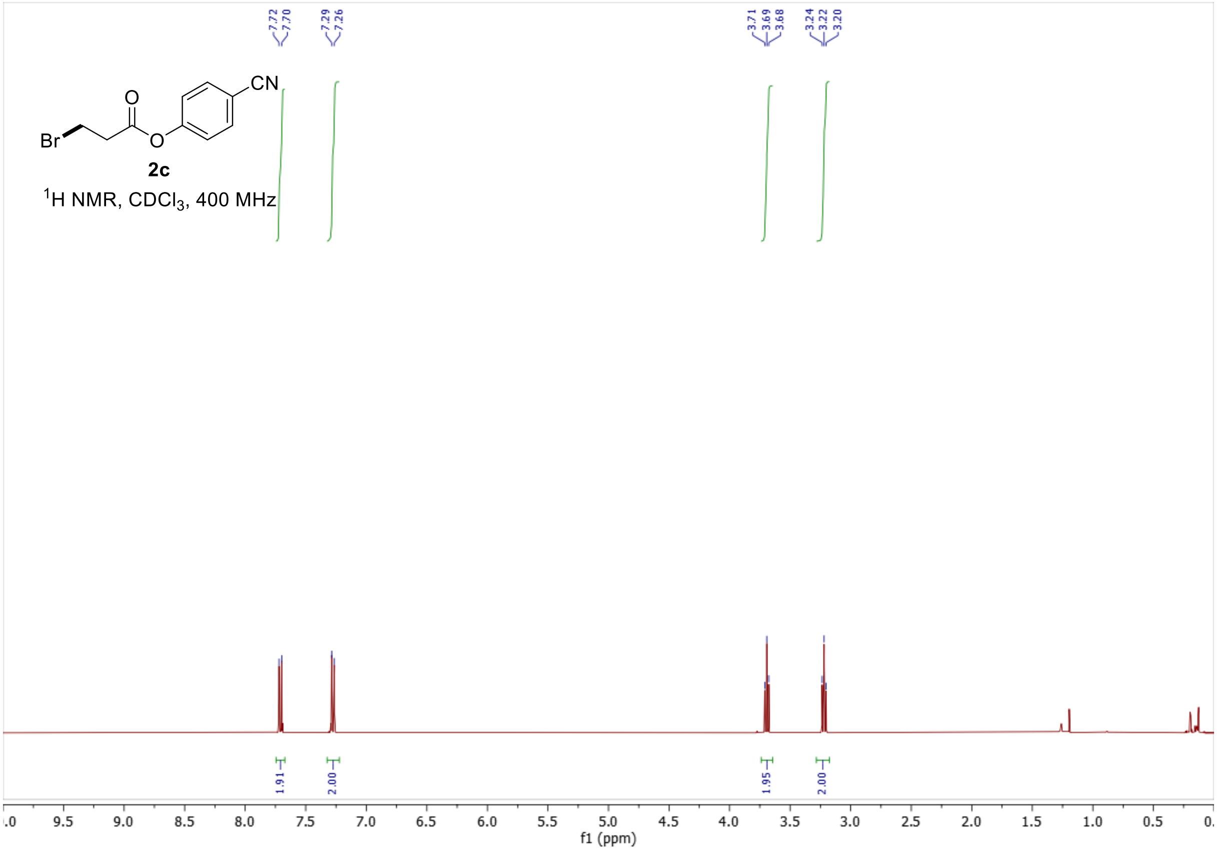

2c
^{1}H NMR, $CDCl_3$, 400 MHz
f1 (ppm)

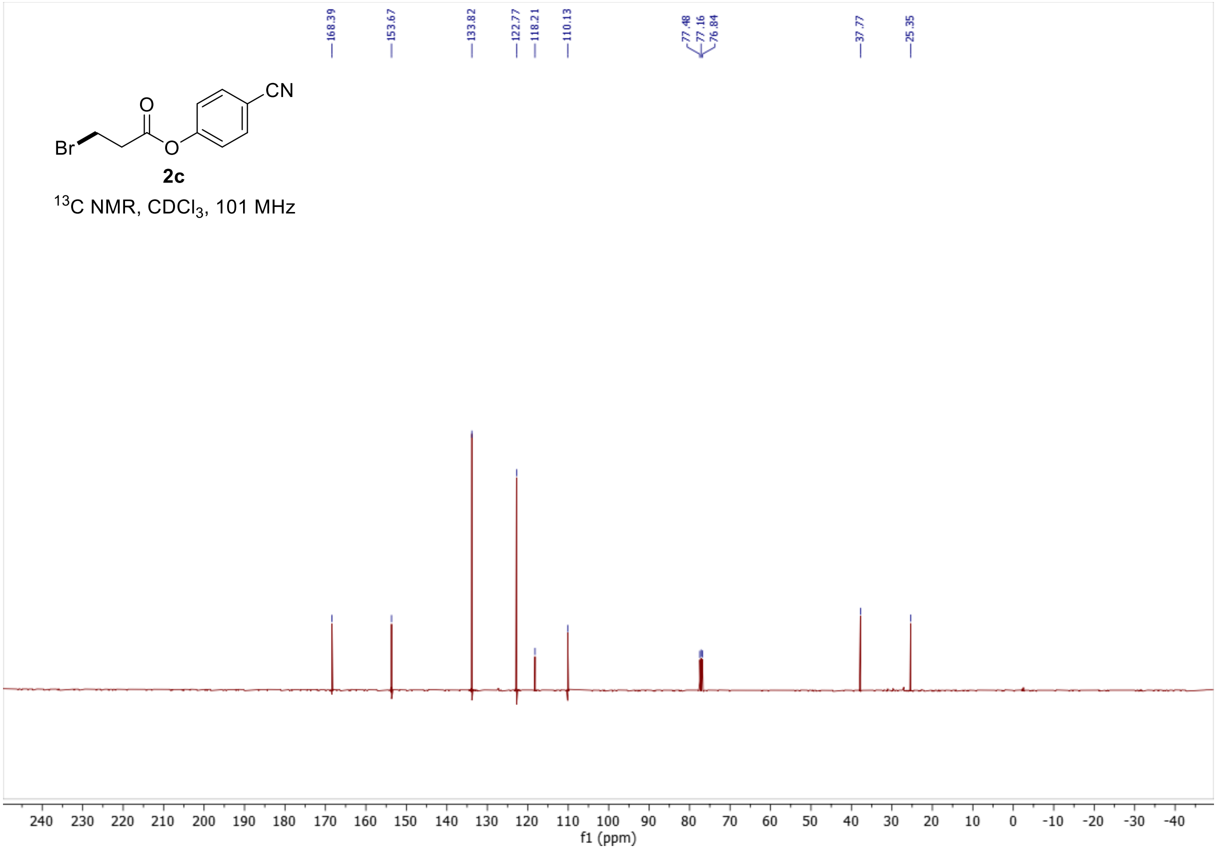

2c
^{13}C NMR, $CDCl_3$, 101 MHz
f1 (ppm)

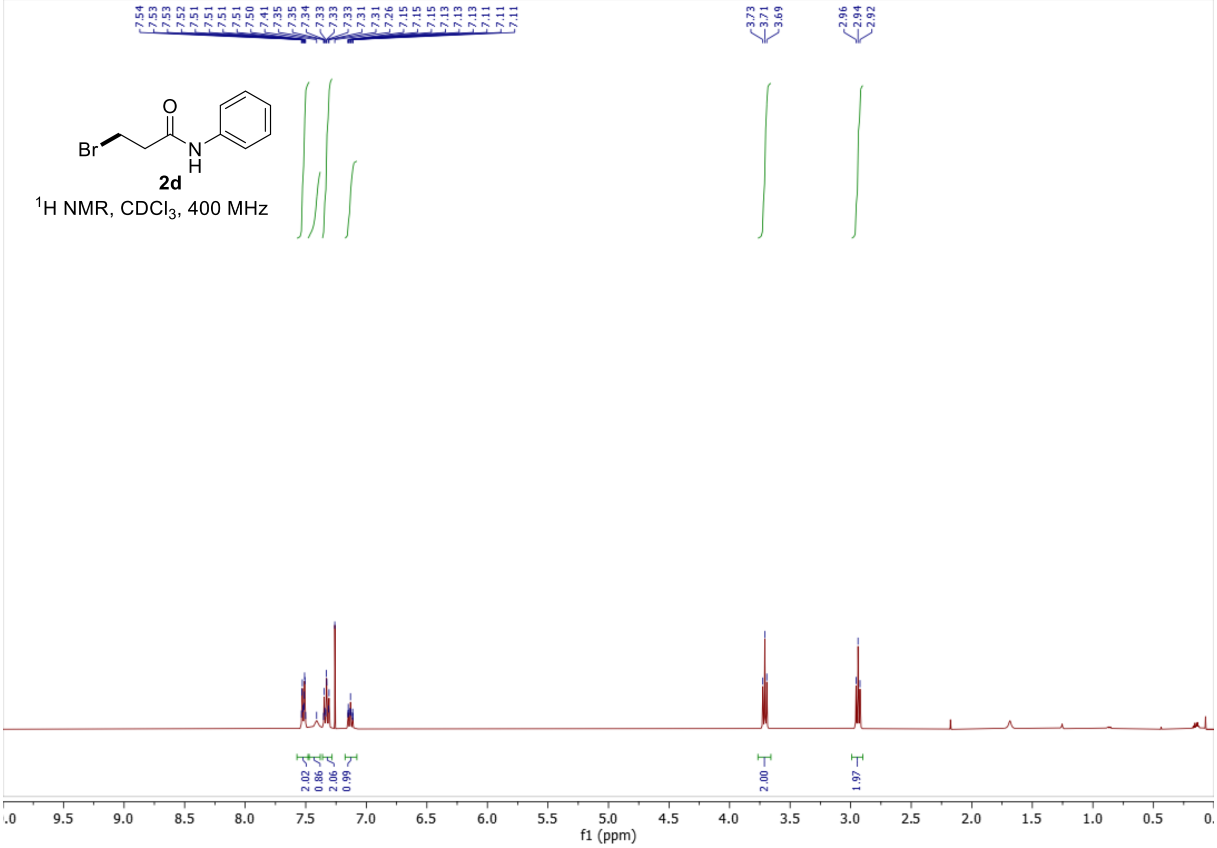

Br
O
N
H
2d
¹H NMR, CDCl3, 400 MHz
f1 (ppm)

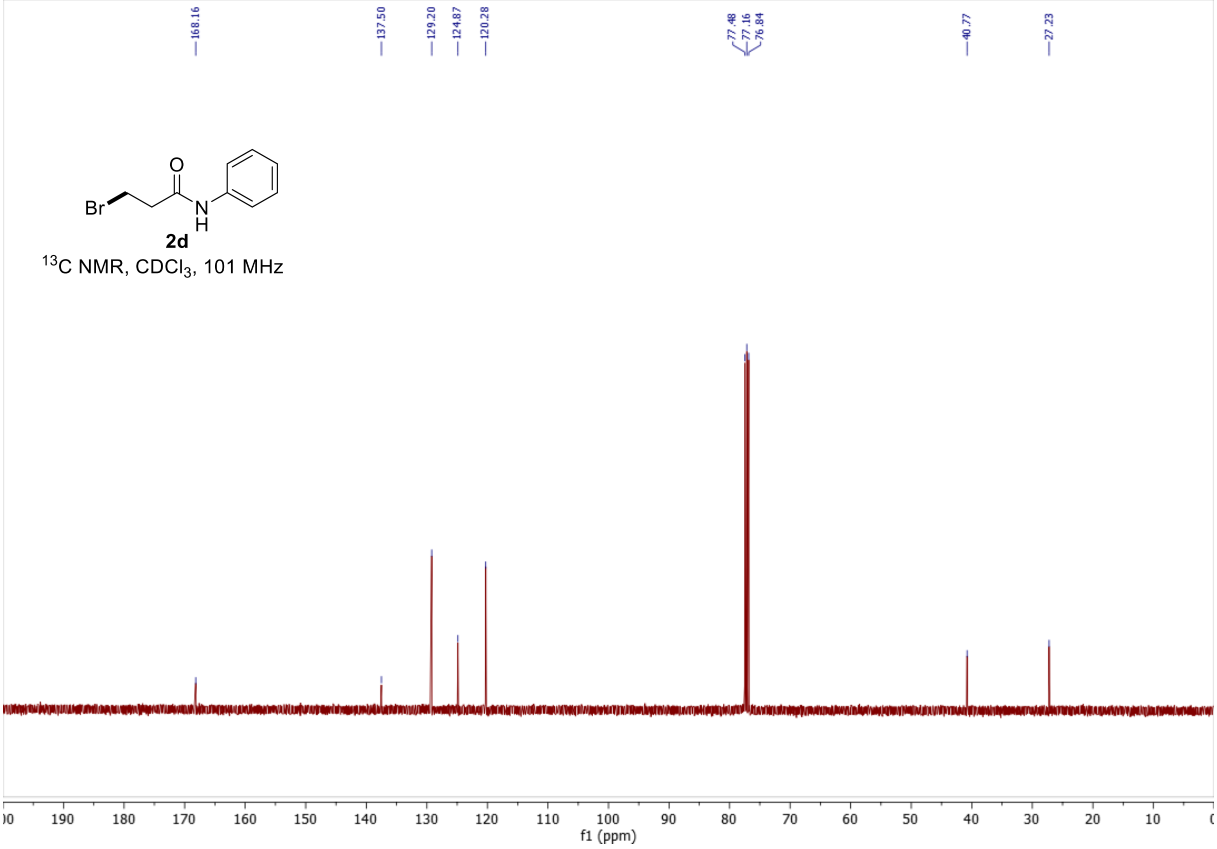

168.16
137.50
129.20
124.87
120.28
77.48
77.16
76.84
40.77
27.23
Br
O
N
H
2d
13C NMR, CDCl3, 101 MHz
f1 (ppm)

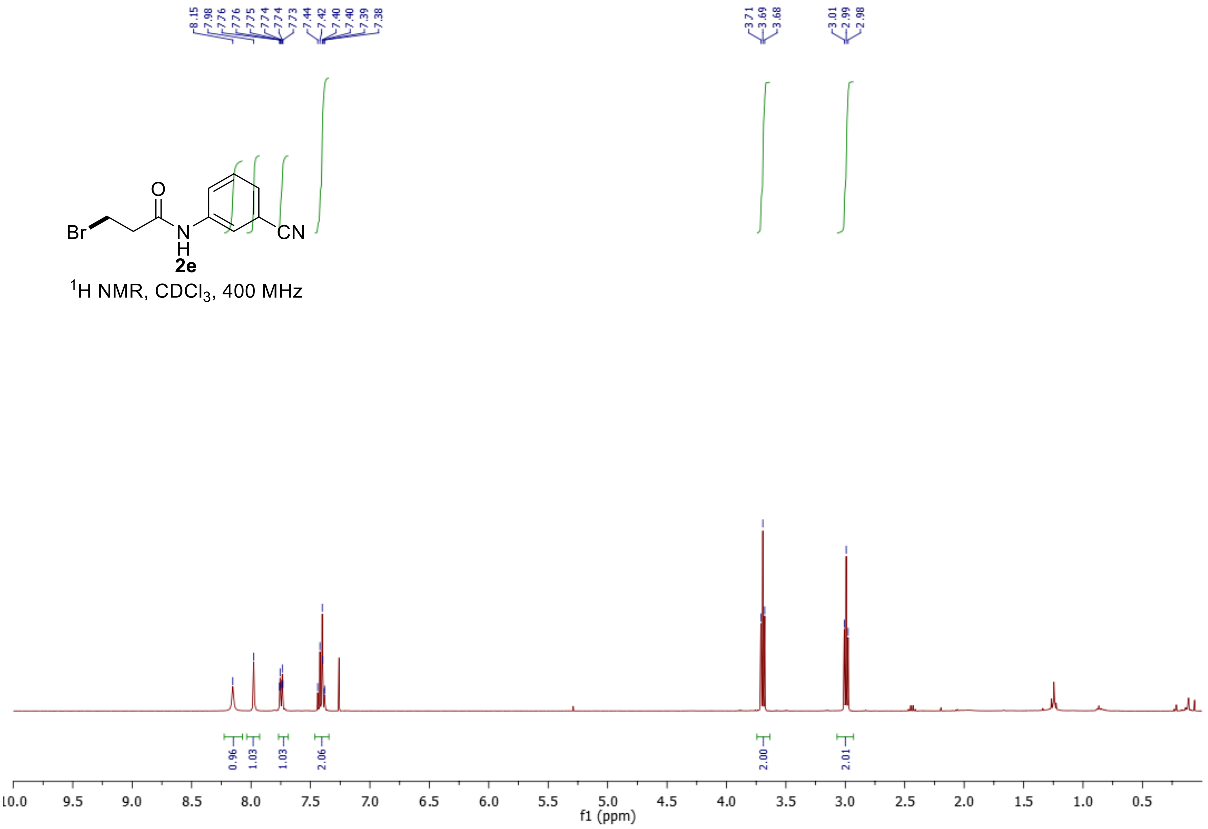

2e

^{1}H NMR, $CDCl_3$, 400 MHz

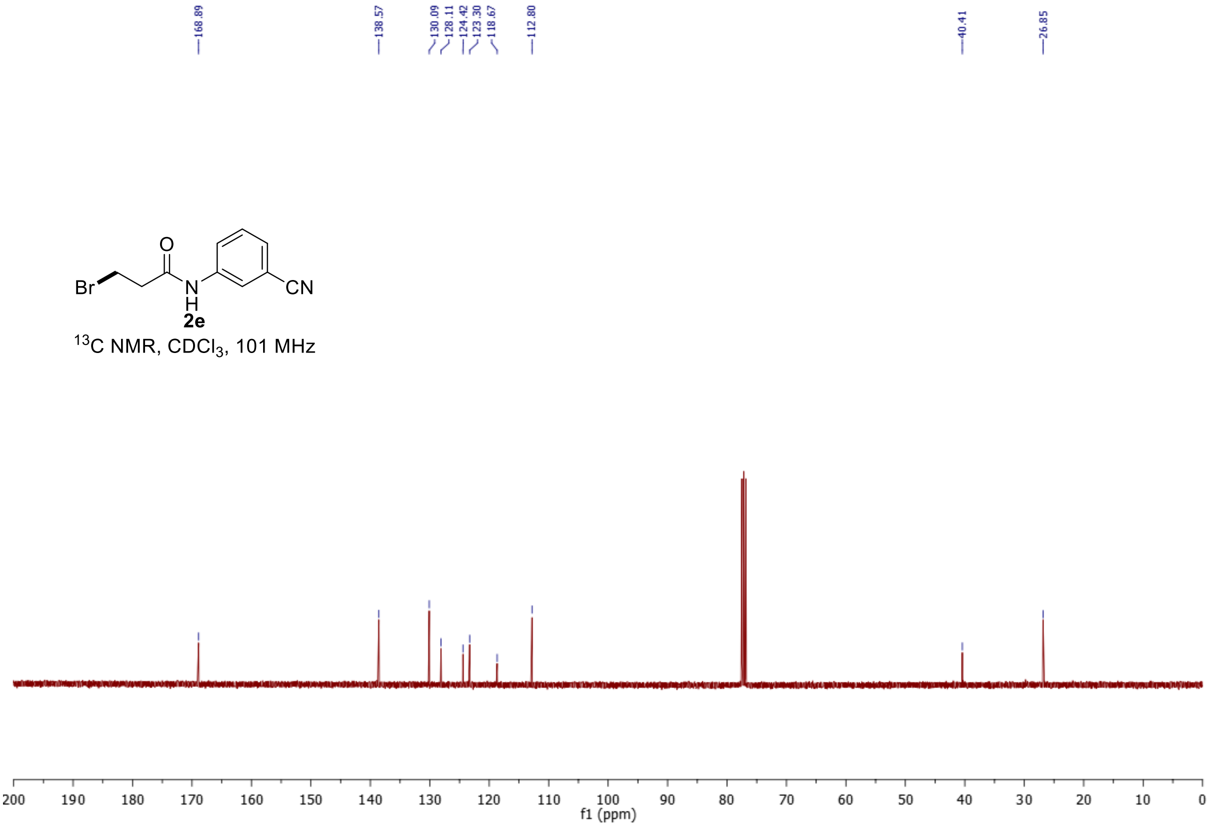

2e

^{13}C NMR, $CDCl_3$, 101 MHz

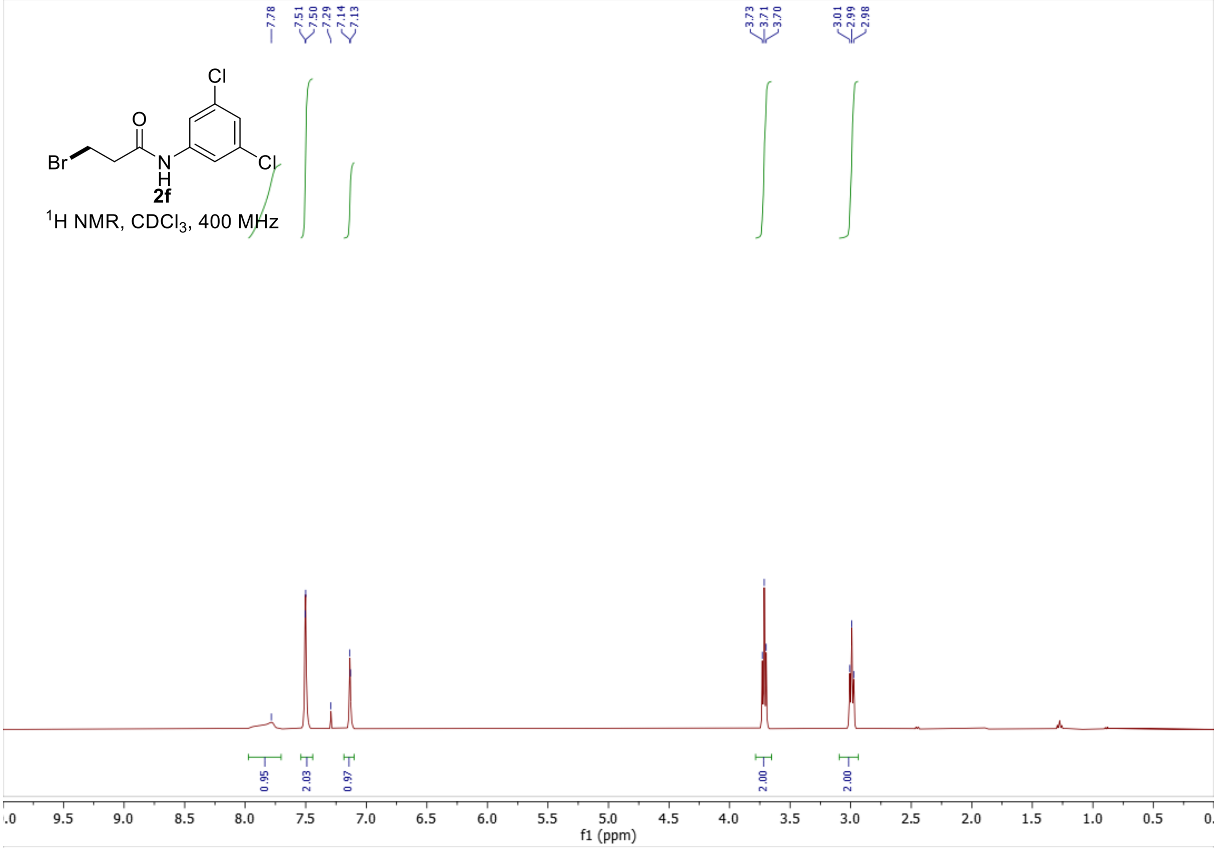
7.78
7.51
7.50
7.29
7.14
7.13
3.73
3.71
3.70
3.01
2.99
2.98
Cl
O
Br
N
H
Cl
2f
^{1}H NMR, $CDCl_3$, 400 MHz
0.95
2.03
0.97
2.00
2.00
f1 (ppm)

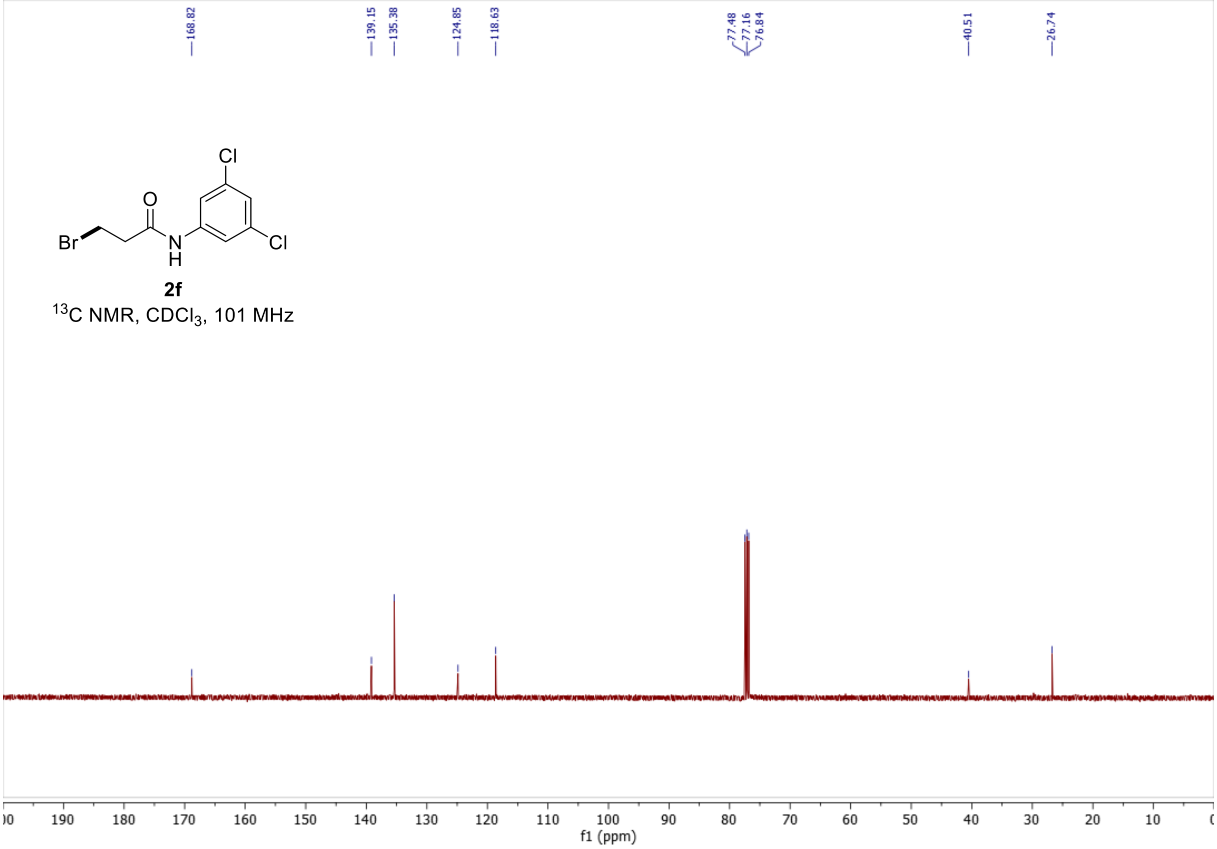
168.82
139.15
135.38
124.85
118.63
77.48
77.16
76.84
40.51
26.74
Cl
O
Br
N
H
Cl
2f
^{13}C NMR, $CDCl_3$, 101 MHz
f1 (ppm)

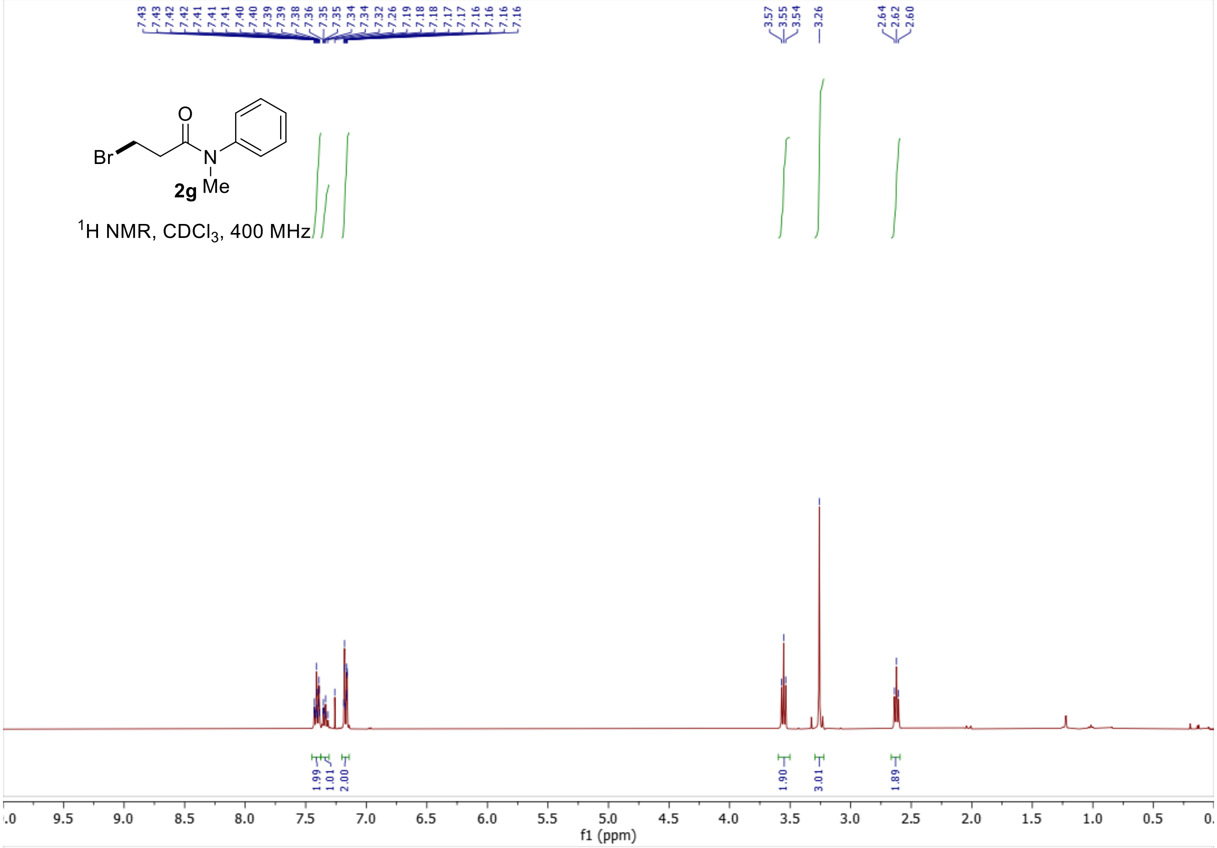
2g
^{1}H NMR, CDCl$_3$, 400 MHz
f1 (ppm)

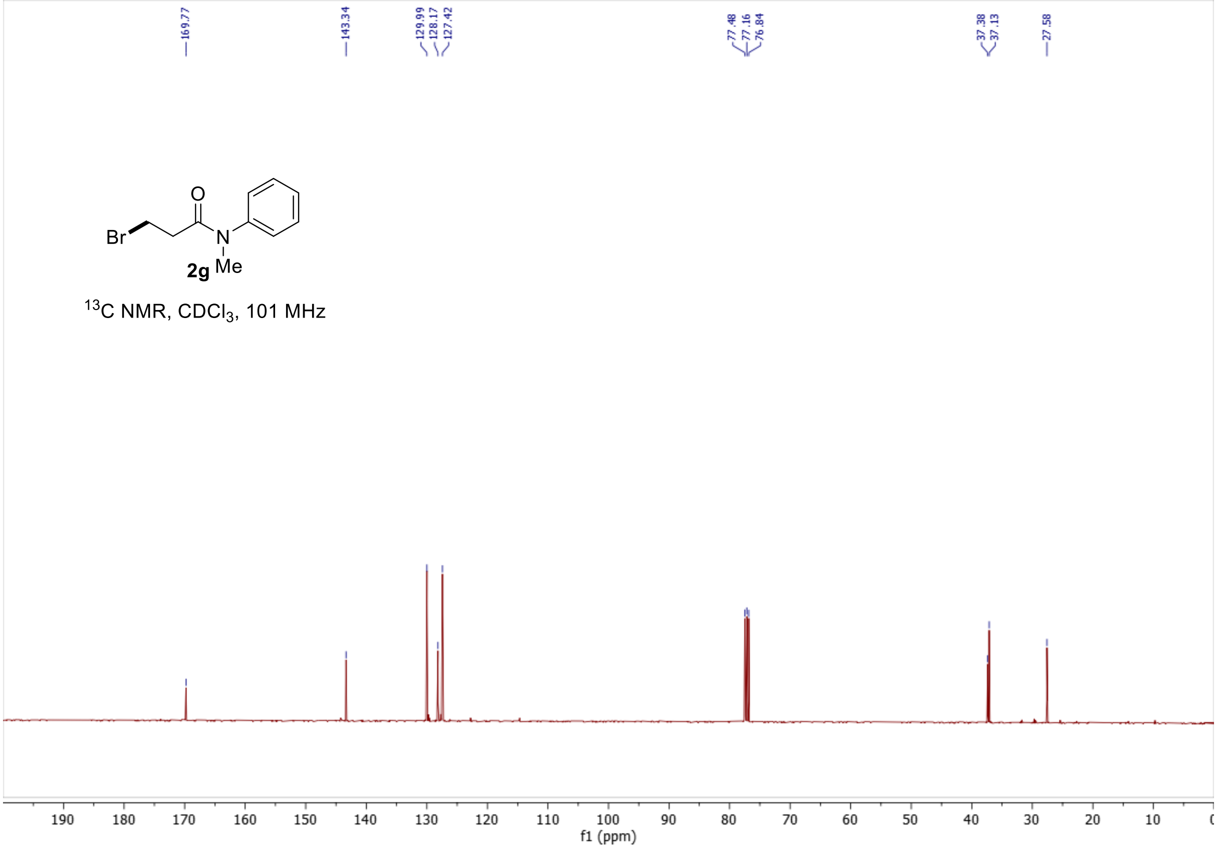
2g
^{13}C NMR, CDCl$_3$, 101 MHz
f1 (ppm)

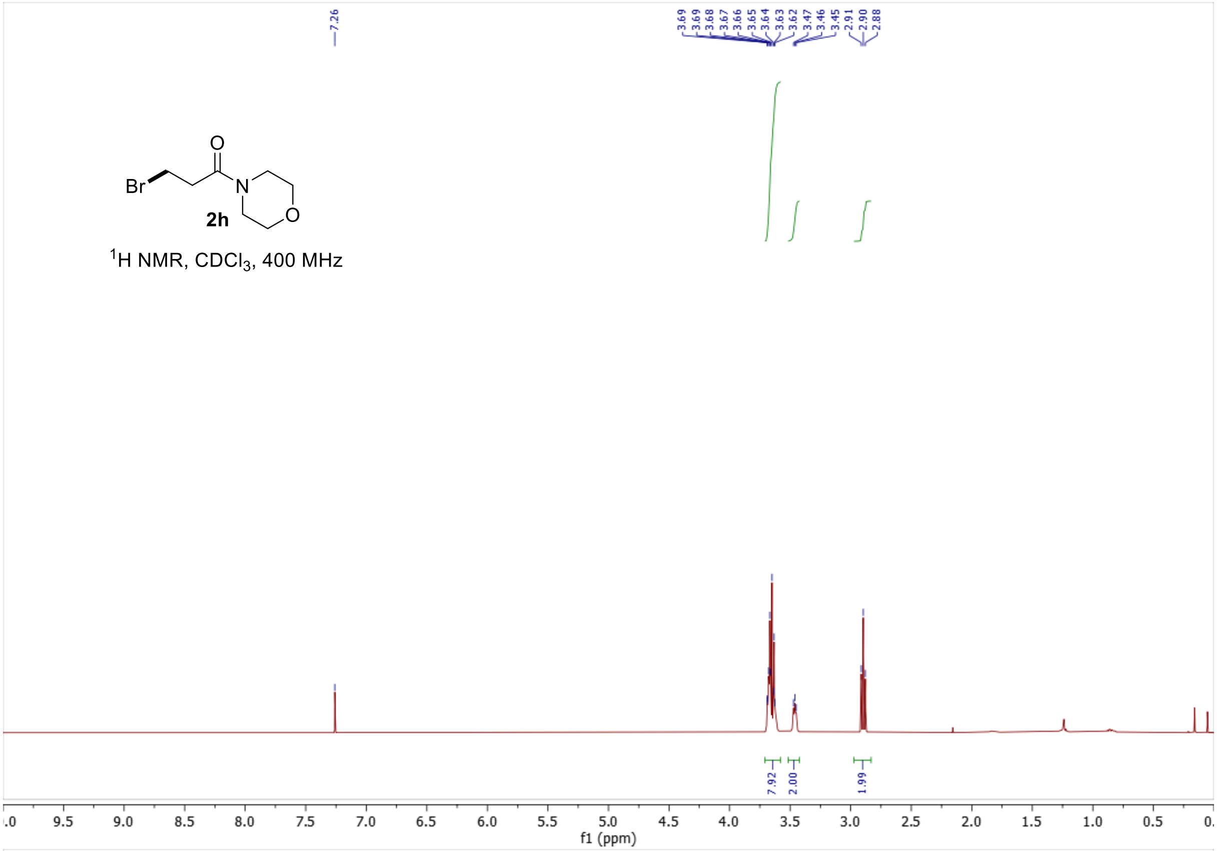

7.26
3.69 3.69 3.68 3.67 3.66 3.65 3.64 3.63 3.62 3.47 3.46 3.45 2.91 2.90 2.88
Br
O
N
O
2h
1H NMR, CDCl3, 400 MHz
7.92
2.00
1.99
f1 (ppm)

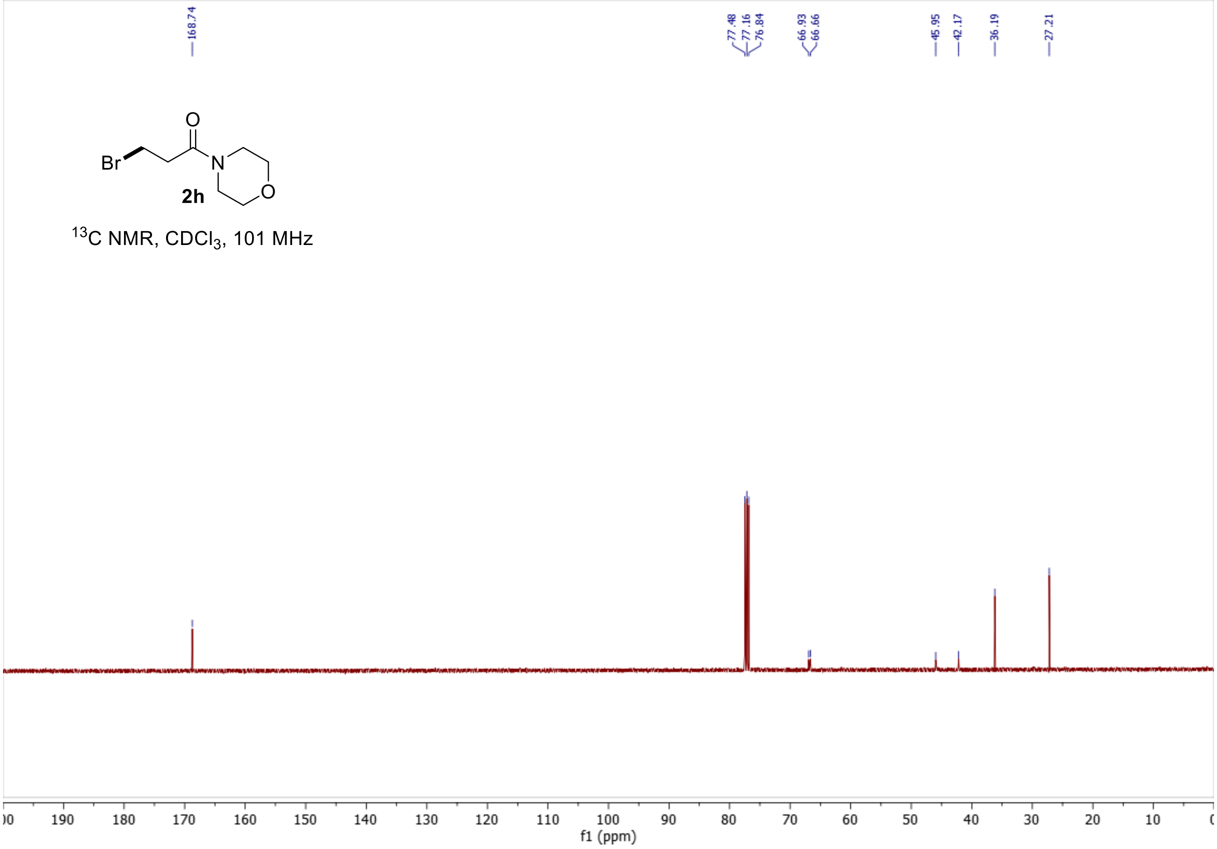

168.74
77.48 77.16 76.84
66.93 66.66
45.95
42.17
36.19
27.21
Br
O
N
O
2h
13C NMR, CDCl3, 101 MHz
f1 (ppm)

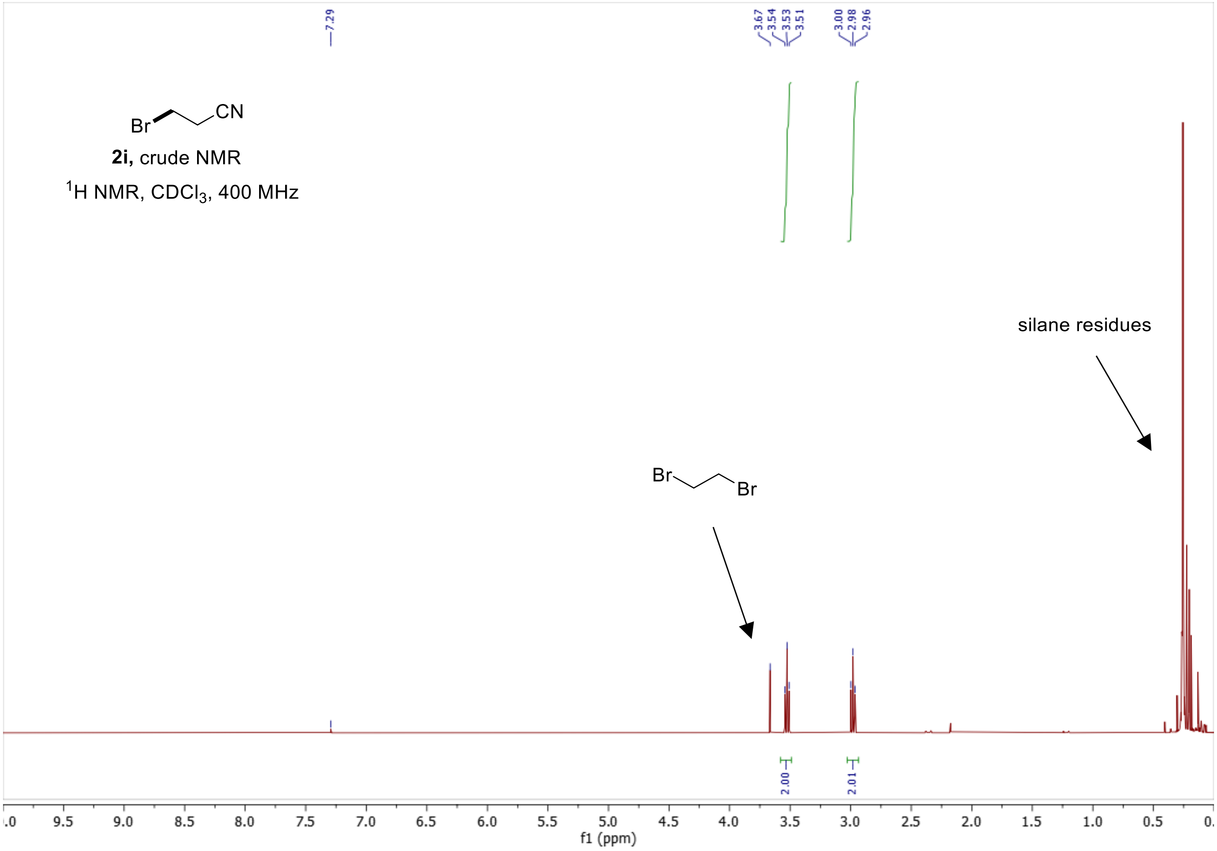

7.29
3.67
3.54
3.53
3.51
3.00
2.98
2.96
Br
CN
2i, crude NMR
1H NMR, CDCl3, 400 MHz
silane residues
Br
Br
2.00
2.01
f1 (ppm)

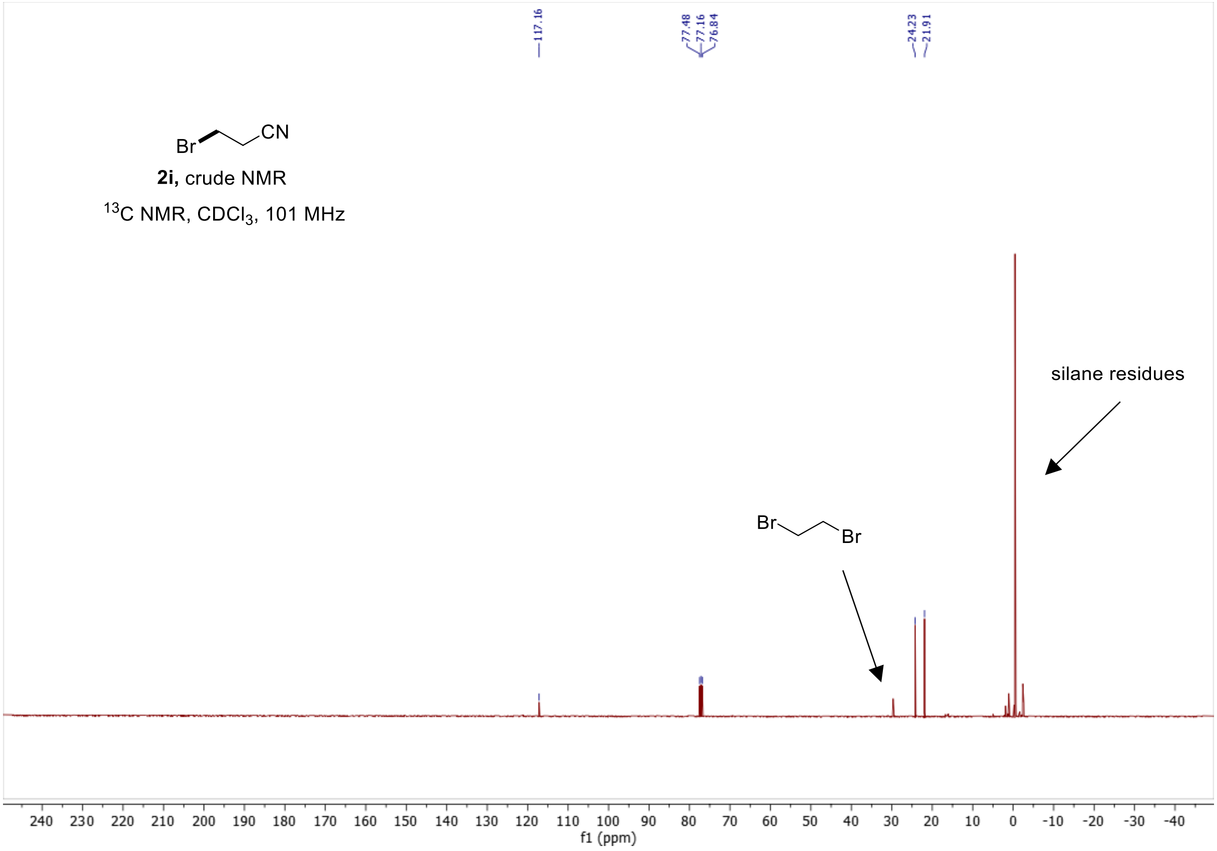

117.16
77.48
77.16
76.84
24.23
21.91
Br
CN
2i, crude NMR
13C NMR, CDCl3, 101 MHz
silane residues
Br
Br
f1 (ppm)

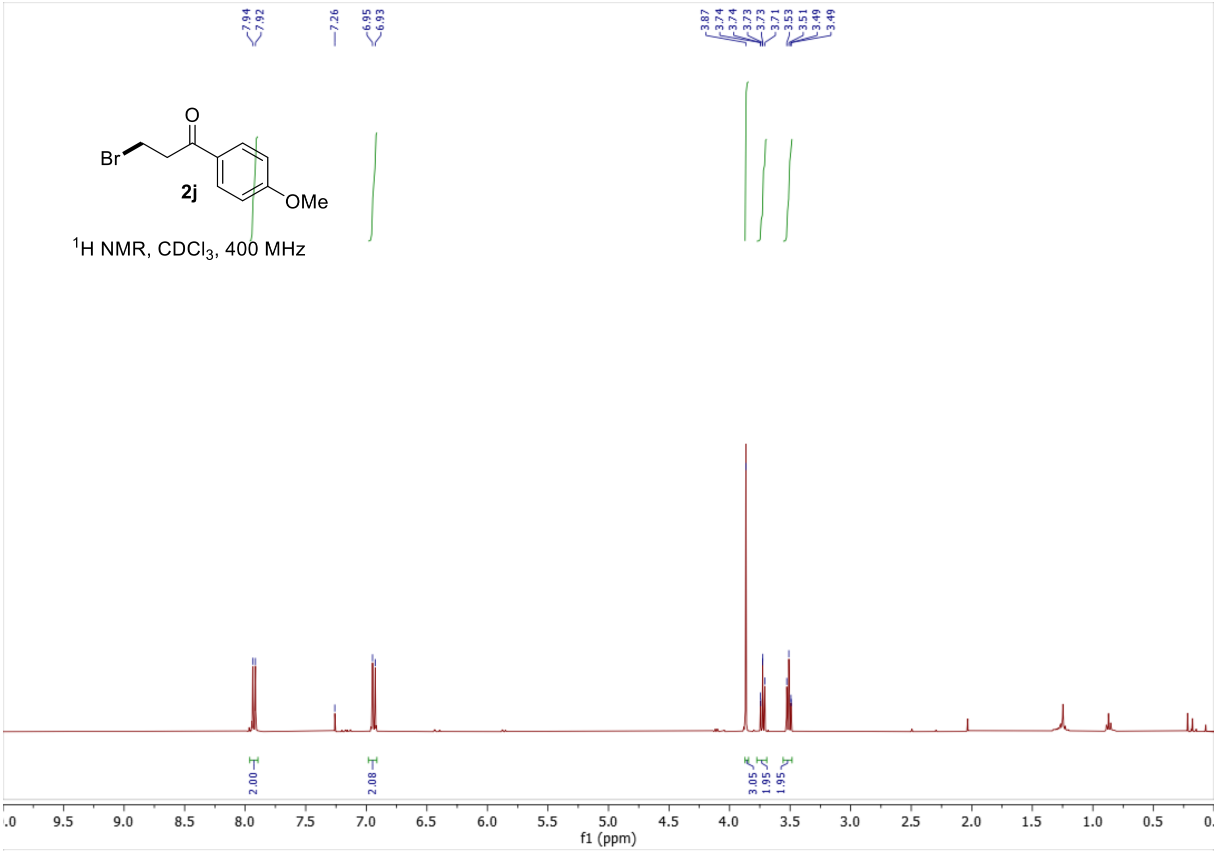
7.94
7.92
7.26
6.95
6.93
3.87
3.74
3.74
3.73
3.73
3.71
3.53
3.51
3.49
3.49
Br
O
2j
OMe
1H NMR, CDCl3, 400 MHz
2.00
2.08
3.05
1.95
1.95
f1 (ppm)

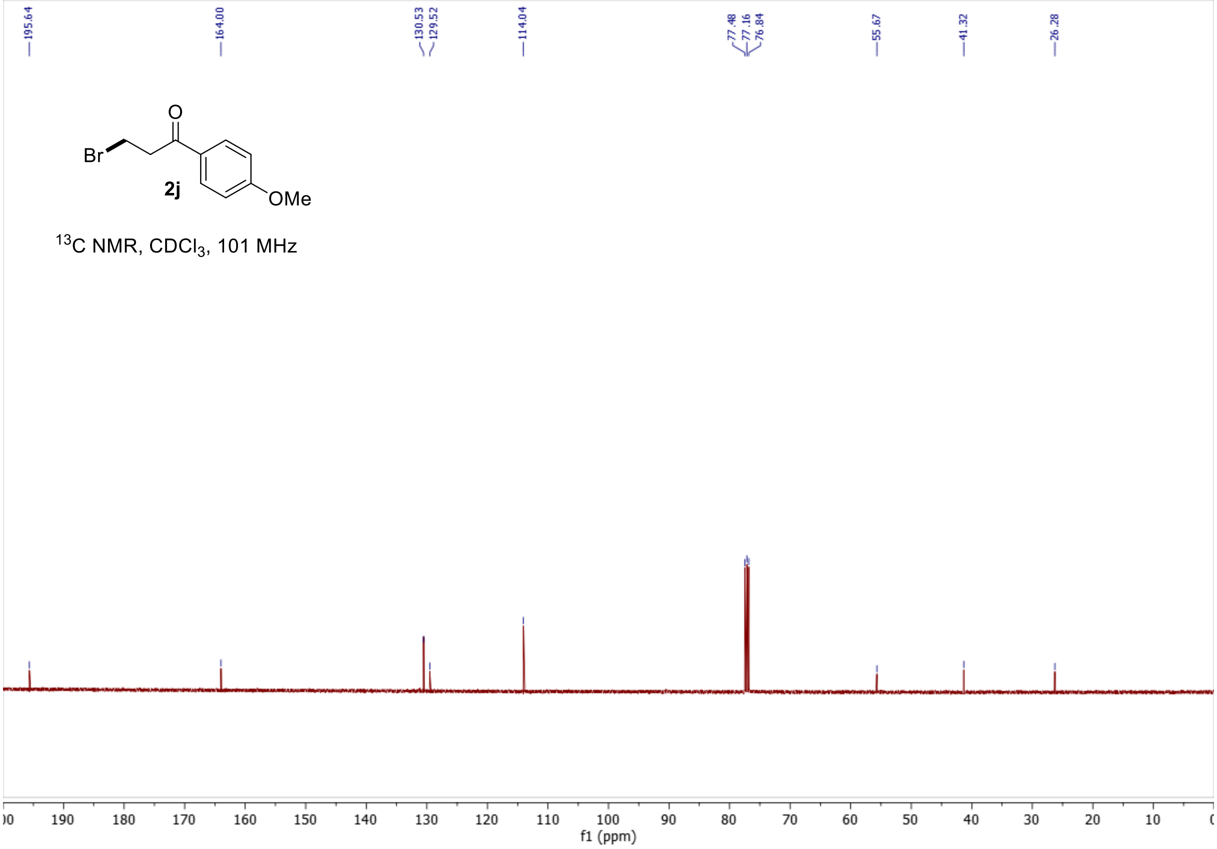
195.64
164.00
130.53
129.52
114.04
77.48
77.16
76.84
55.67
41.32
26.28
Br
O
2j
OMe
13C NMR, CDCl3, 101 MHz
f1 (ppm)

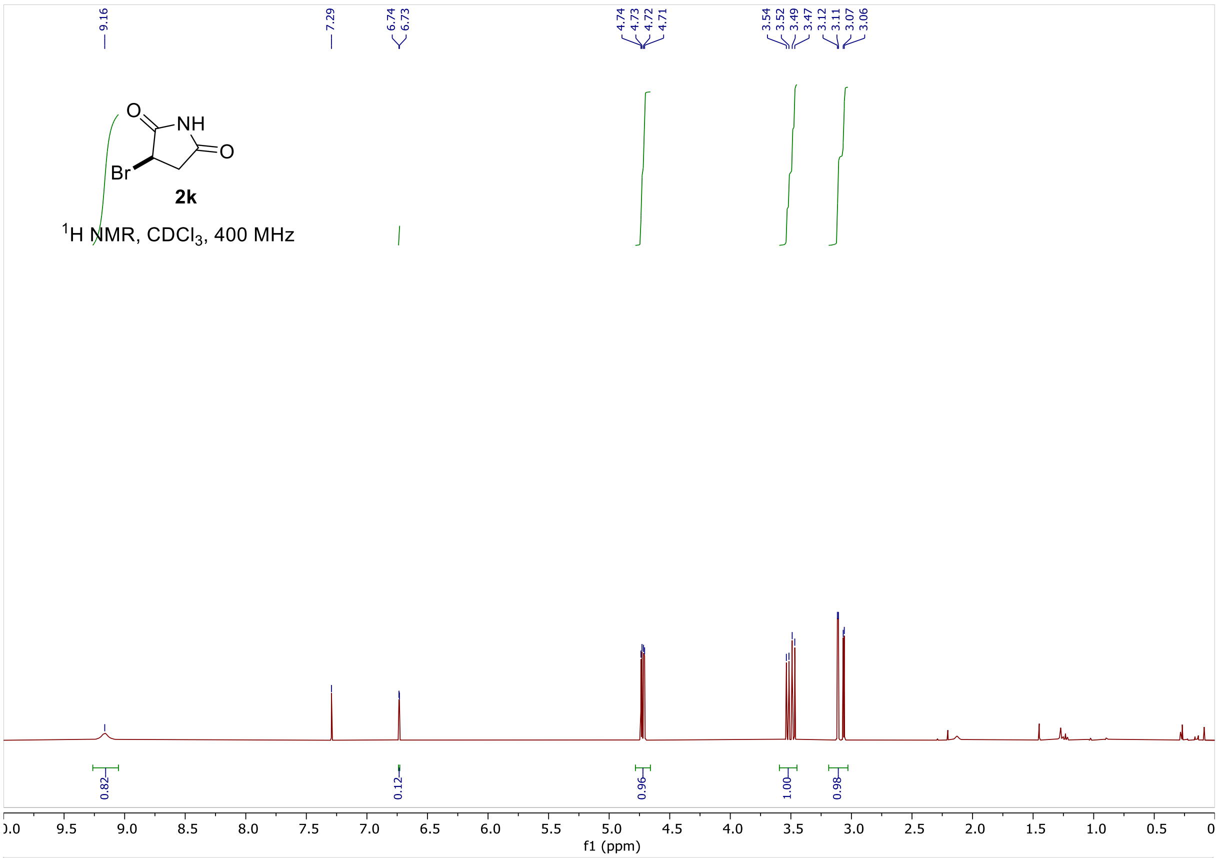
9.16
7.29
6.74
6.73
4.74
4.73
4.72
4.71
3.54
3.52
3.49
3.47
3.12
3.11
3.07
3.06
O
NH
O
Br
2k
^{1}H NMR, CDCl$_3$, 400 MHz
0.82
0.12
0.96
1.00
0.98
f1 (ppm)

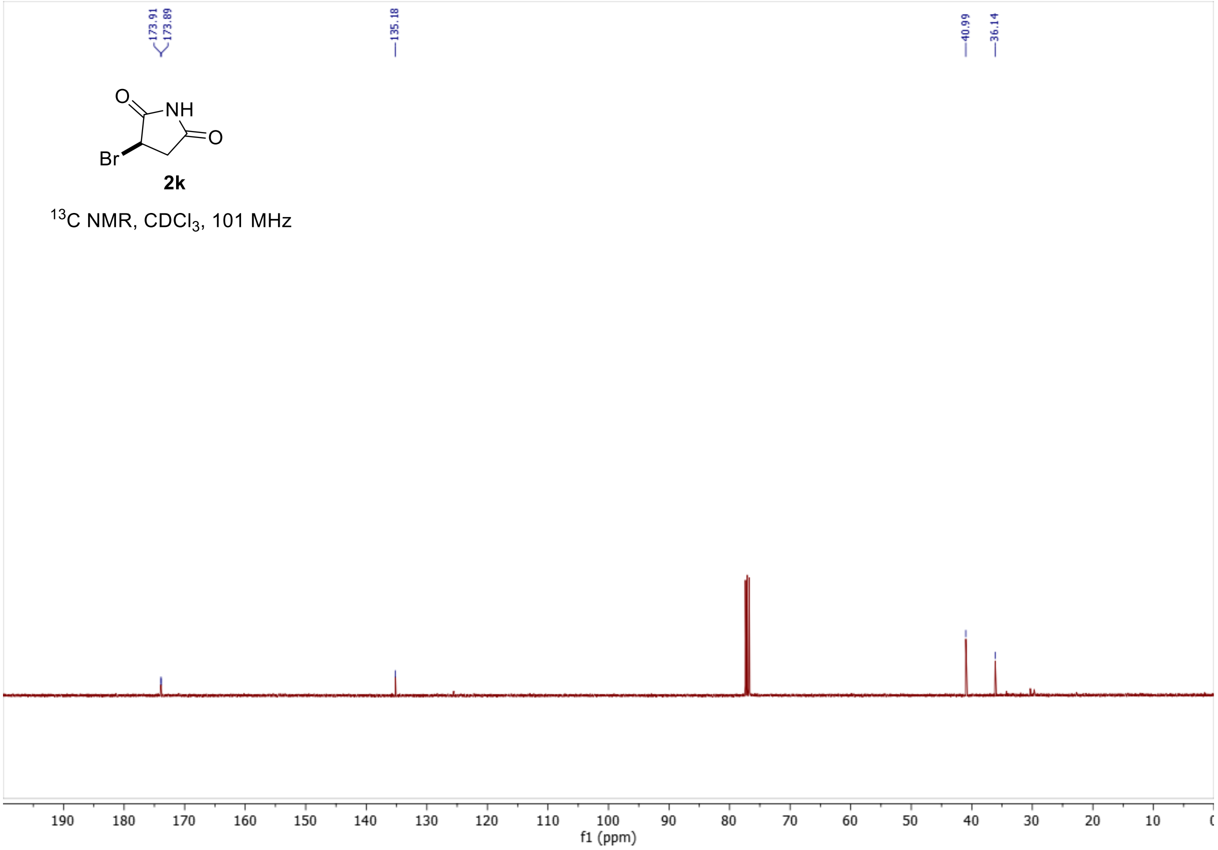
173.91
173.89
135.18
40.99
36.14
O
NH
O
Br
2k
^{13}C NMR, CDCl$_3$, 101 MHz
f1 (ppm)

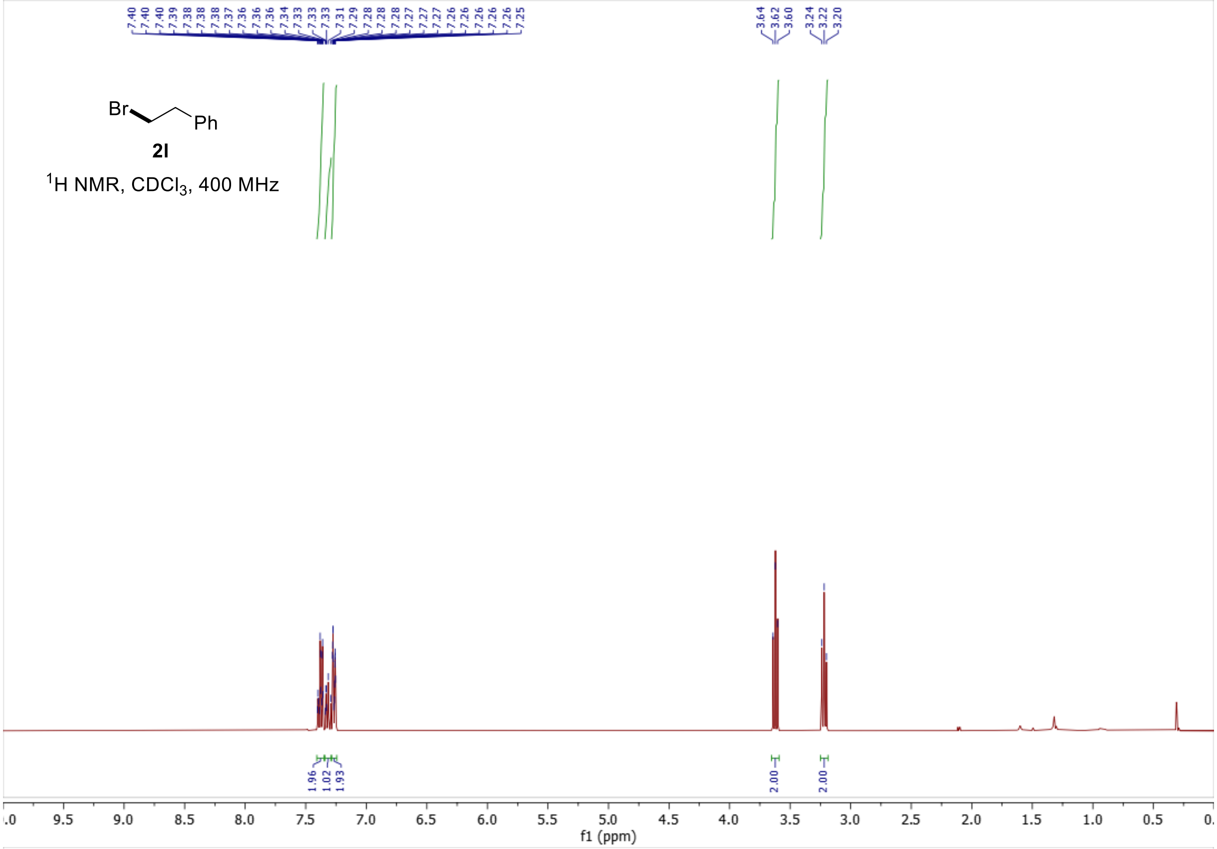
Br
Ph
2l
^{1}H NMR, CDCl$_3$, 400 MHz
f1 (ppm)

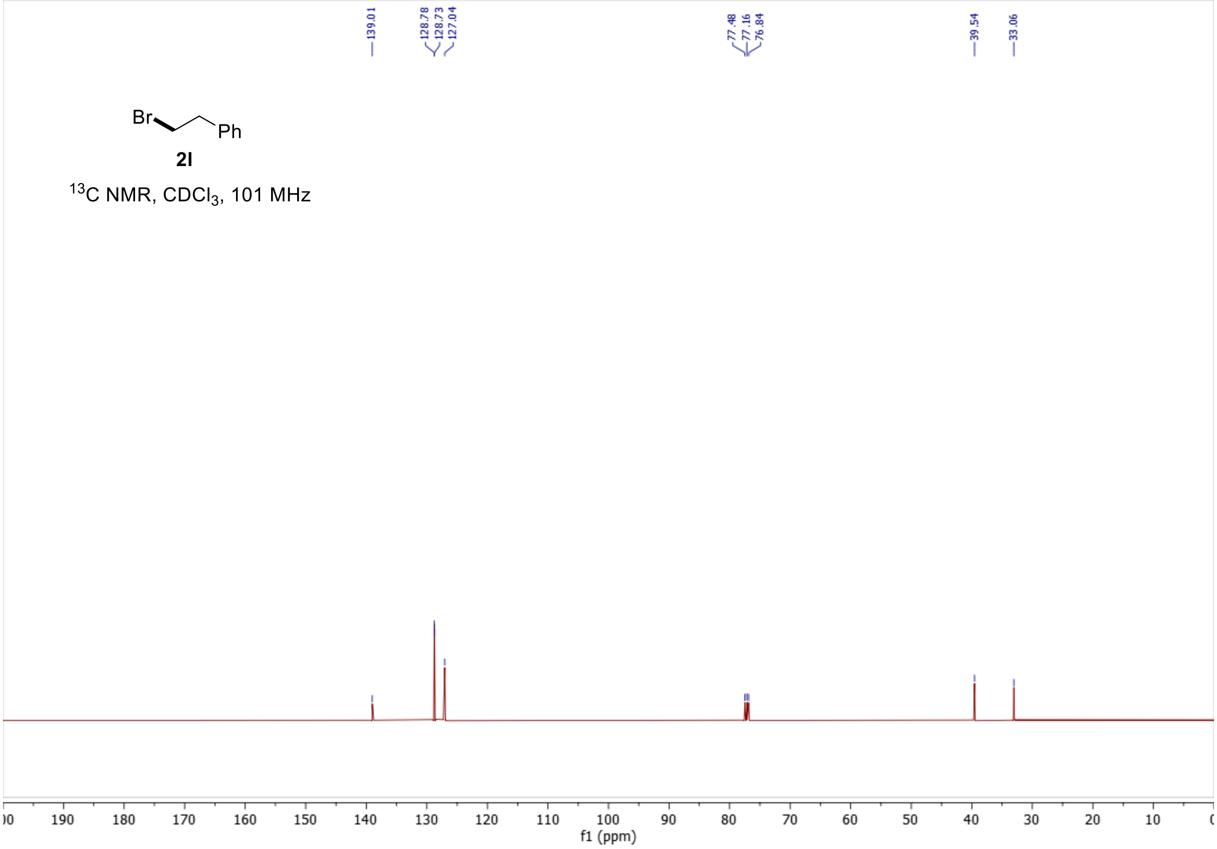
Br
Ph
2l
^{13}C NMR, CDCl$_3$, 101 MHz
139.01
128.78
128.73
127.04
77.48
77.16
76.84
39.54
33.06
f1 (ppm)

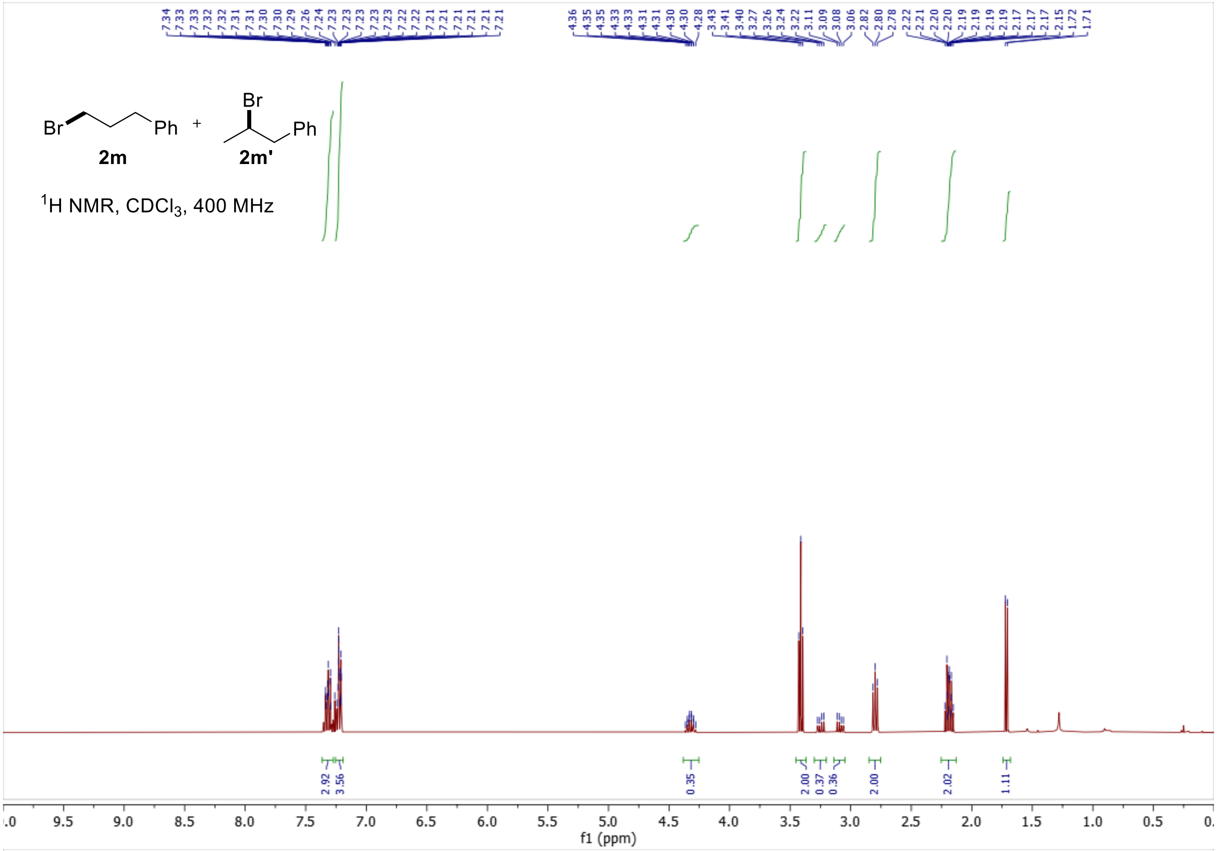
2m
2m'
^{1}H NMR, $CDCl_3$, 400 MHz
f1 (ppm)

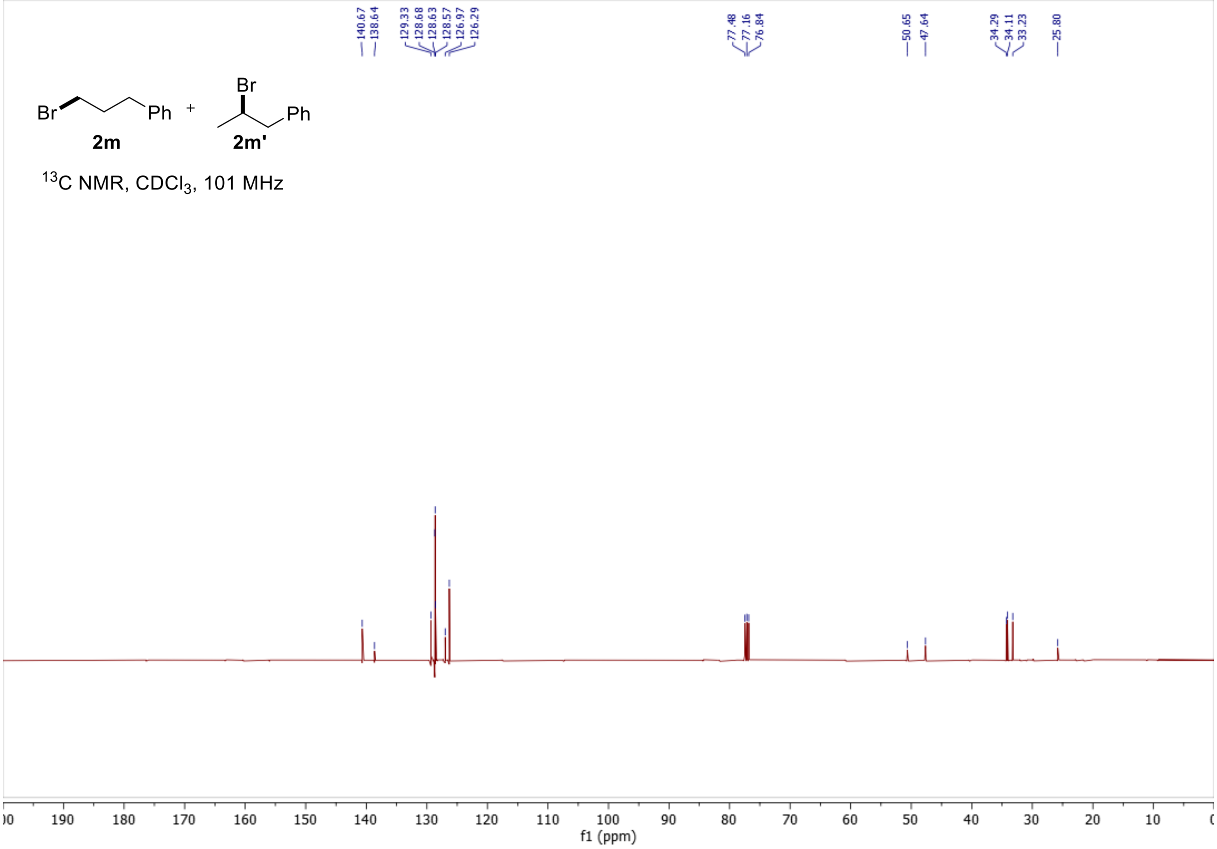
2m
2m'
^{13}C NMR, $CDCl_3$, 101 MHz
f1 (ppm)

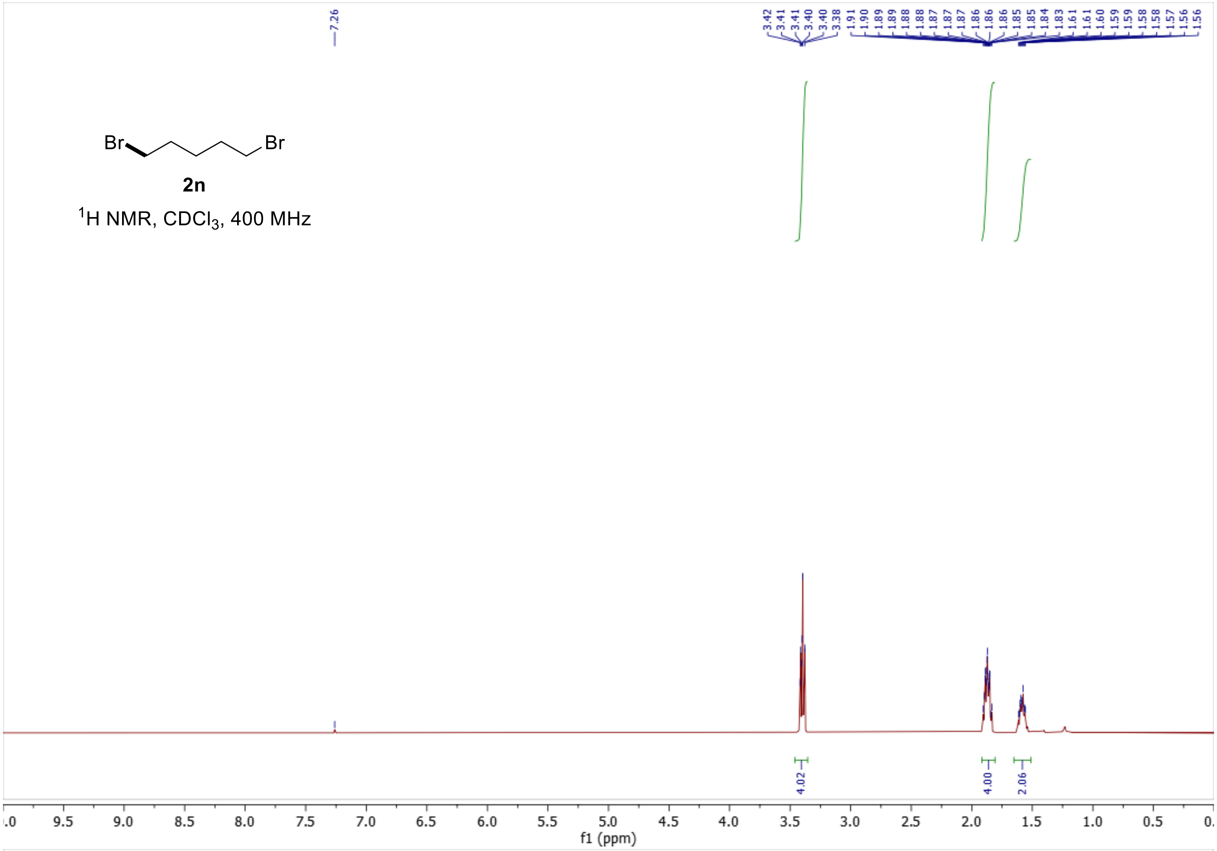
Br
Br
2n
¹H NMR, CDCl₃, 400 MHz
f1 (ppm)

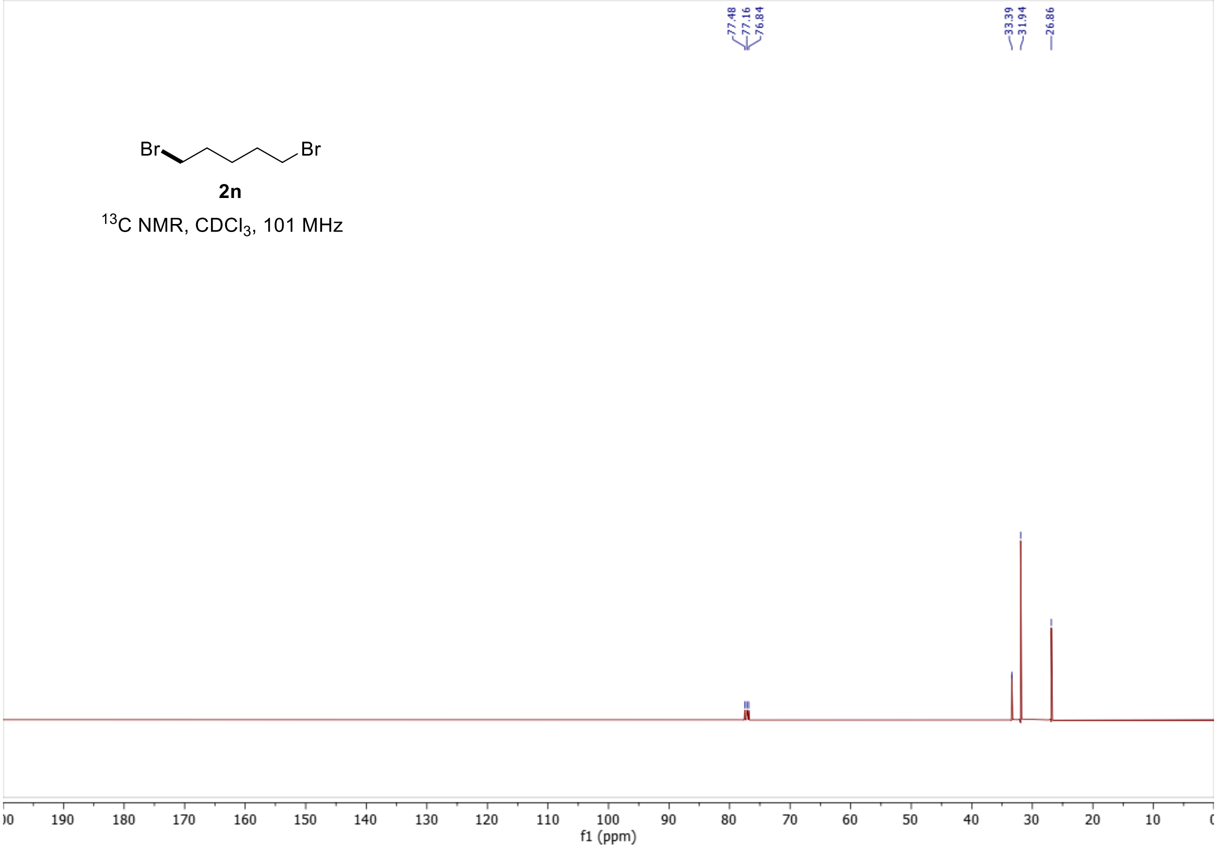
Br
Br
2n
¹³C NMR, CDCl₃, 101 MHz
f1 (ppm)

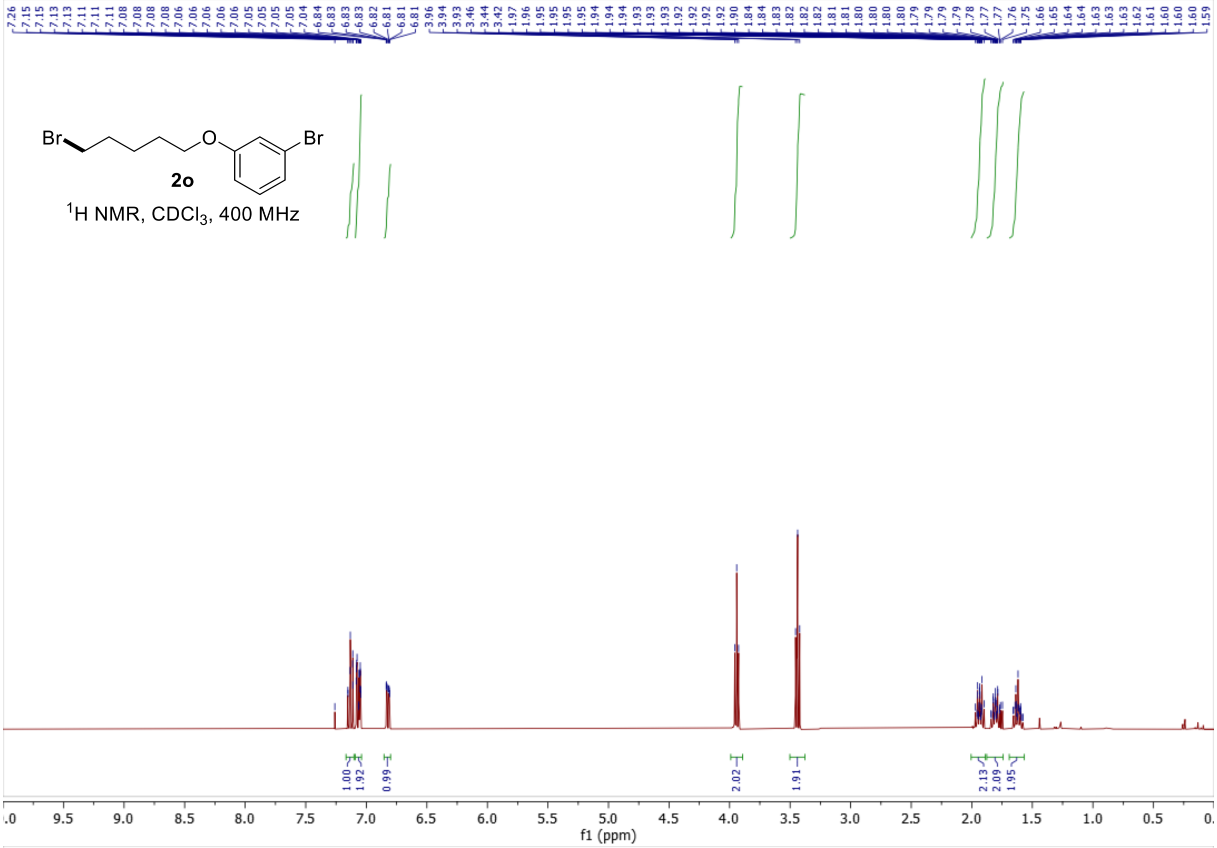
Br
O
Br
2o
^{1}H NMR, CDCl$_3$, 400 MHz
f1 (ppm)

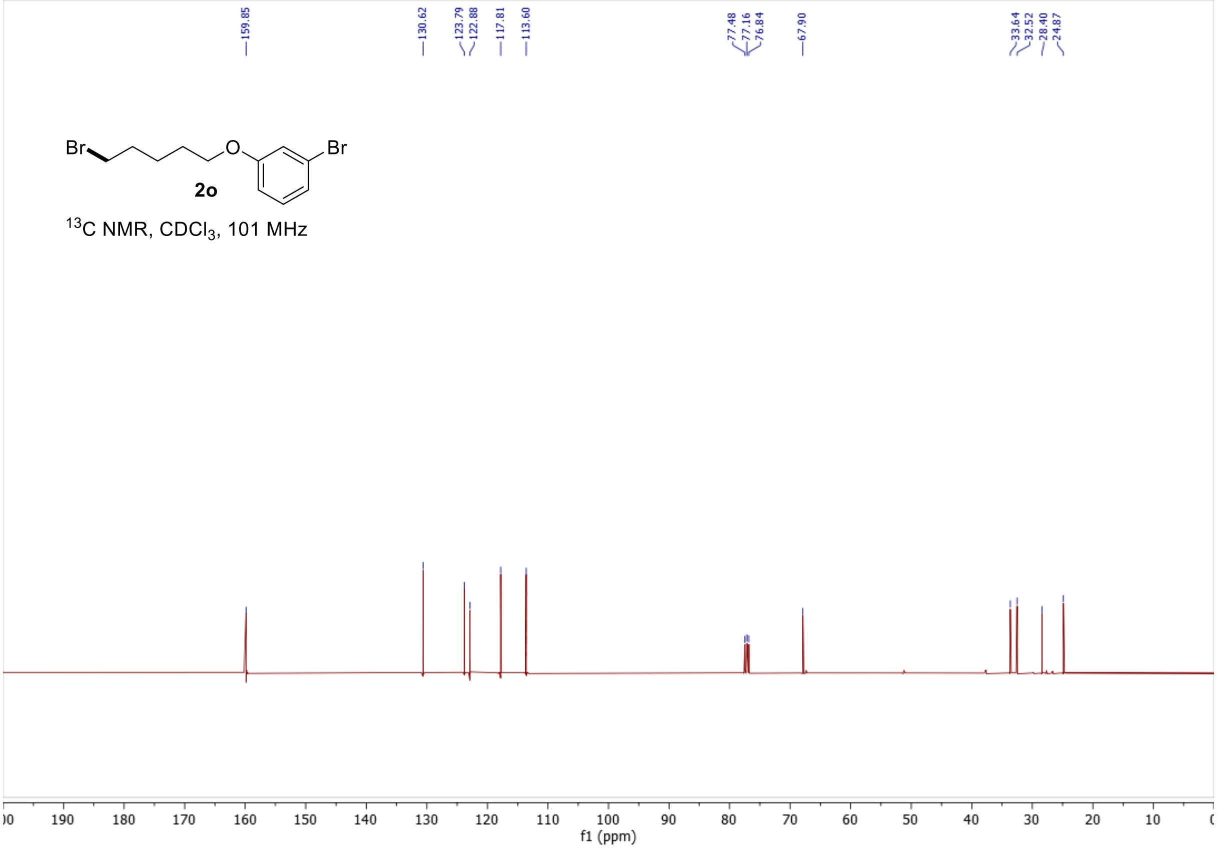
Br
O
Br
2o
^{13}C NMR, CDCl$_3$, 101 MHz
f1 (ppm)

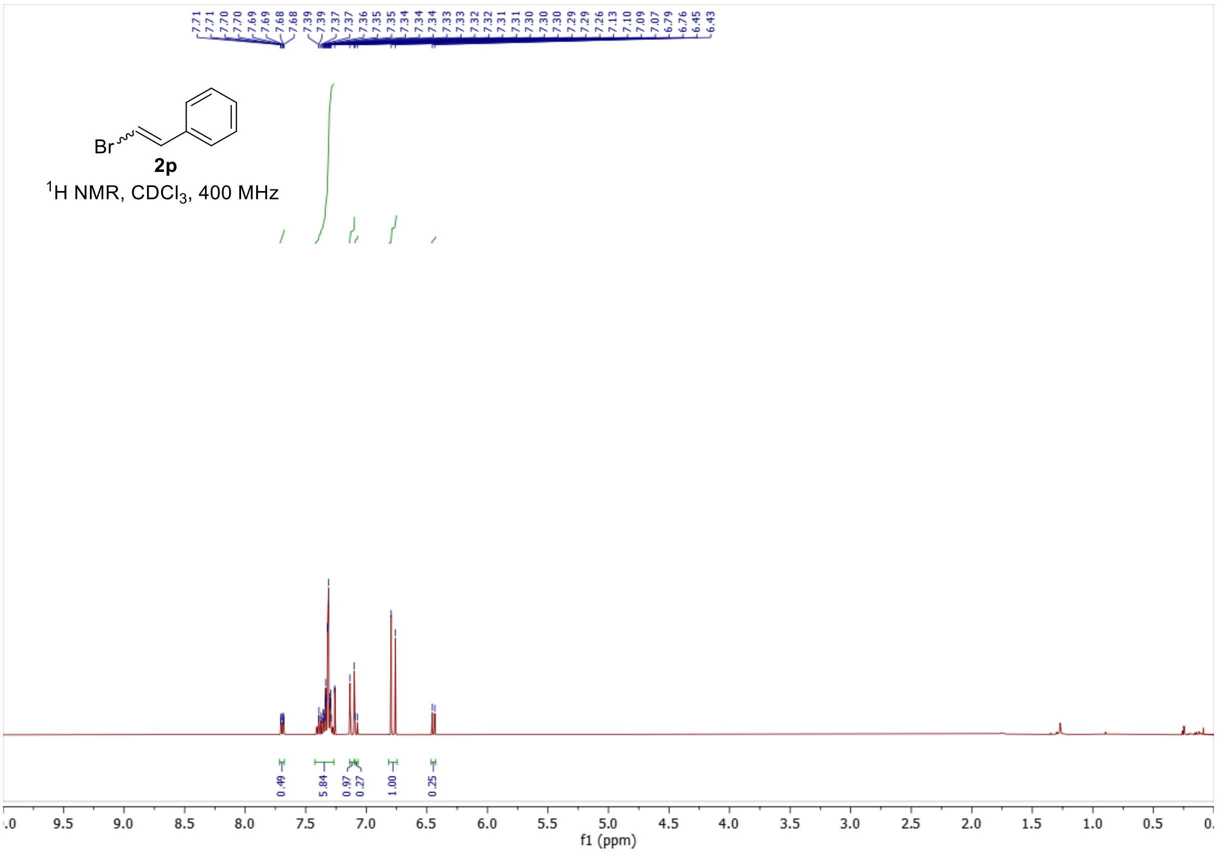

Br
2p
1H NMR, CDCl3, 400 MHz
f1 (ppm)

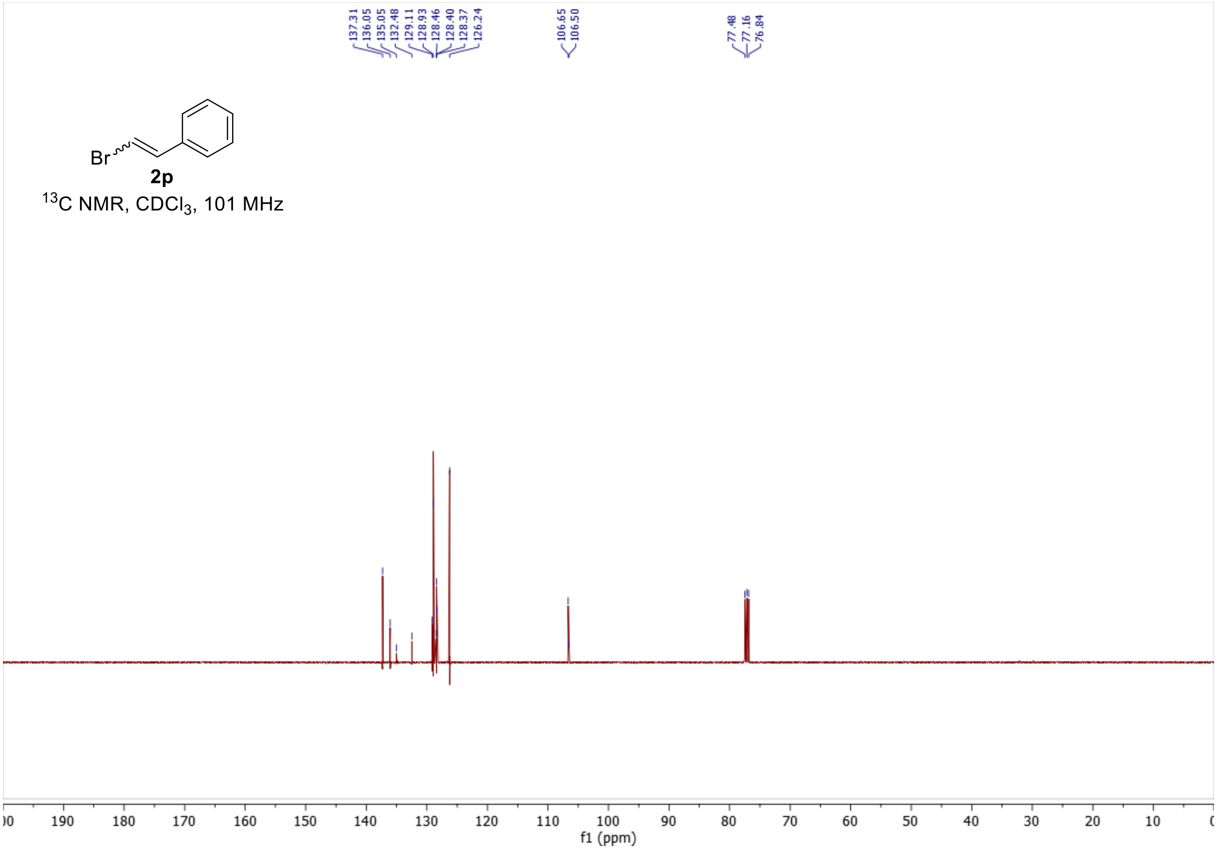

Br
2p
13C NMR, CDCl3, 101 MHz
f1 (ppm)

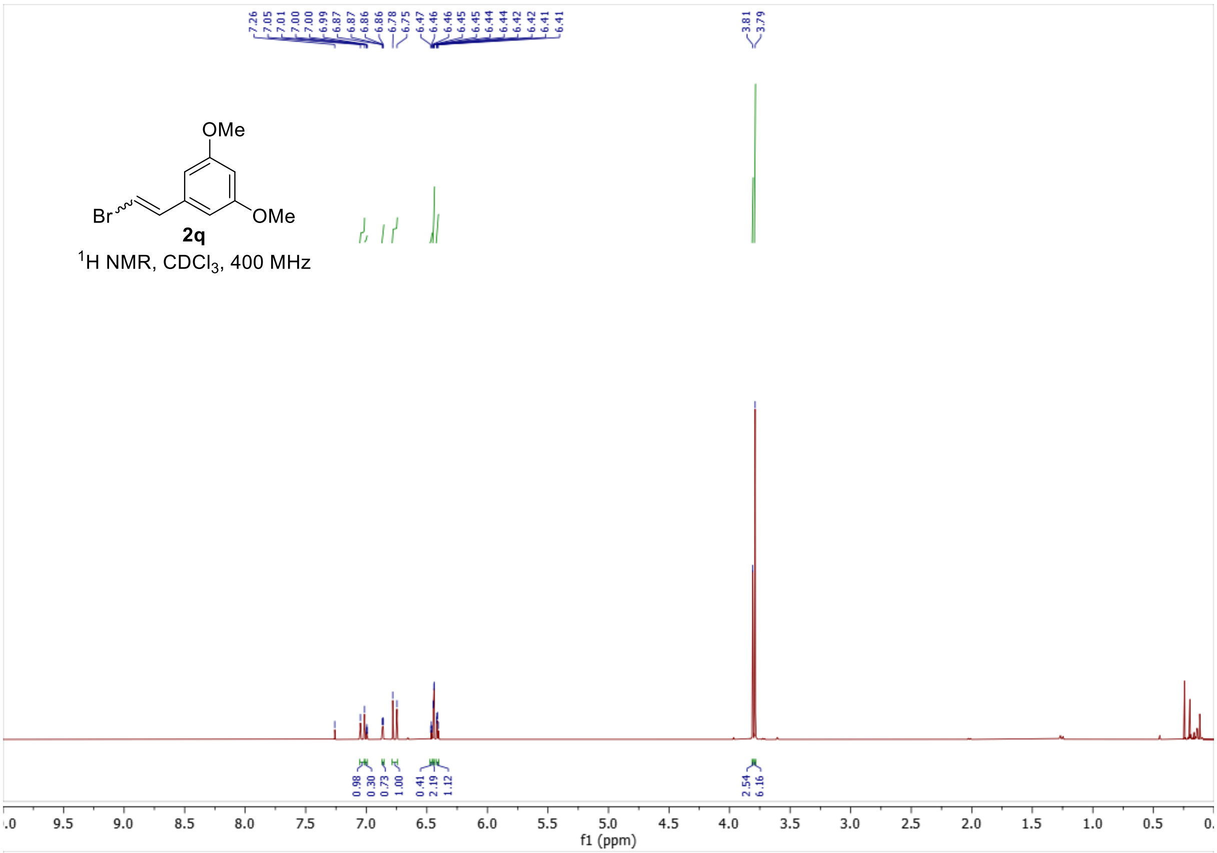
OMe
Br
OMe
2q
1H NMR, CDCl3, 400 MHz
7.26 7.05 7.01 7.00 7.00 6.99 6.87 6.87 6.86 6.86 6.78 6.75 6.47 6.46 6.46 6.45 6.45 6.44 6.44 6.42 6.42 6.41 6.41
3.81 3.79
0.98 0.30 0.73 1.00 0.41 2.19 1.12
2.54 6.16
f1 (ppm)

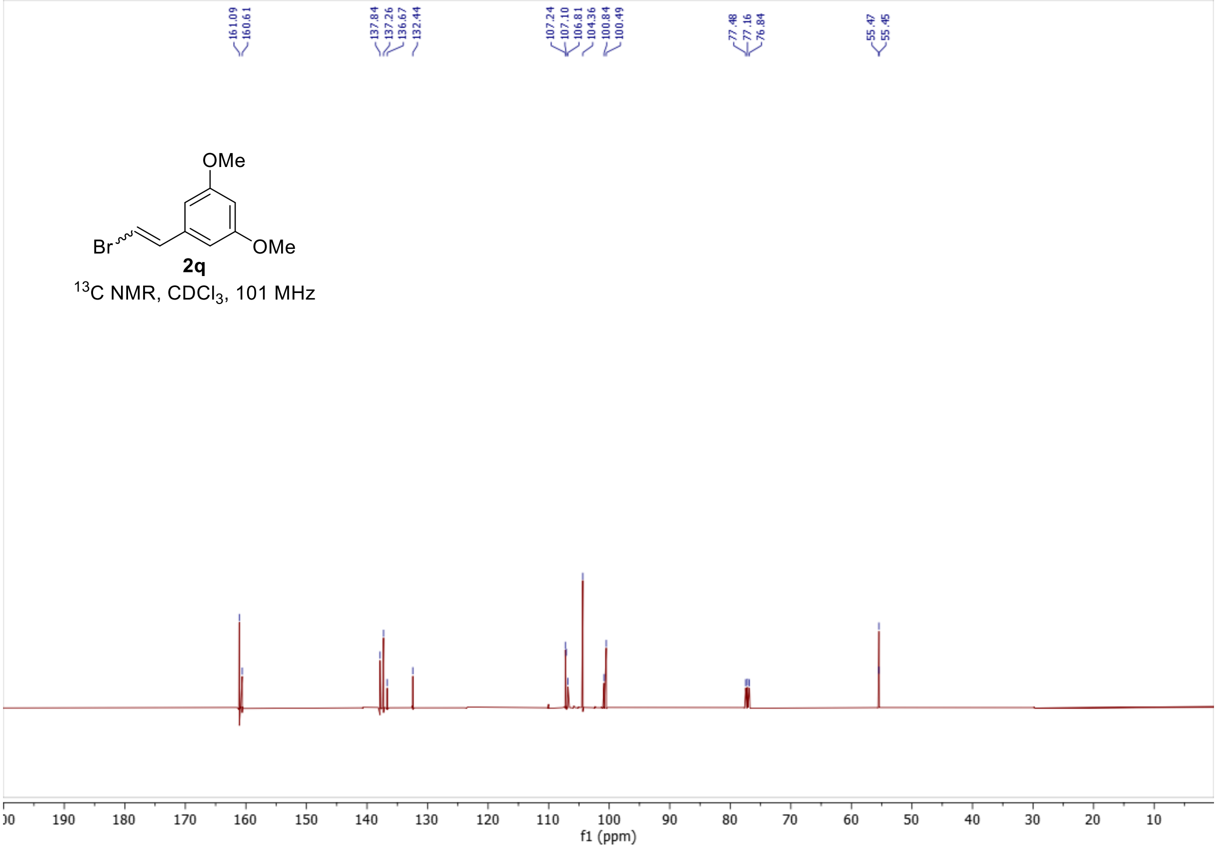
OMe
Br
OMe
2q
13C NMR, CDCl3, 101 MHz
161.09 160.61
137.84 137.26 136.67 132.44
107.24 107.10 106.81 104.36 100.84 100.49
77.48 77.16 76.84
55.47 55.45
f1 (ppm)

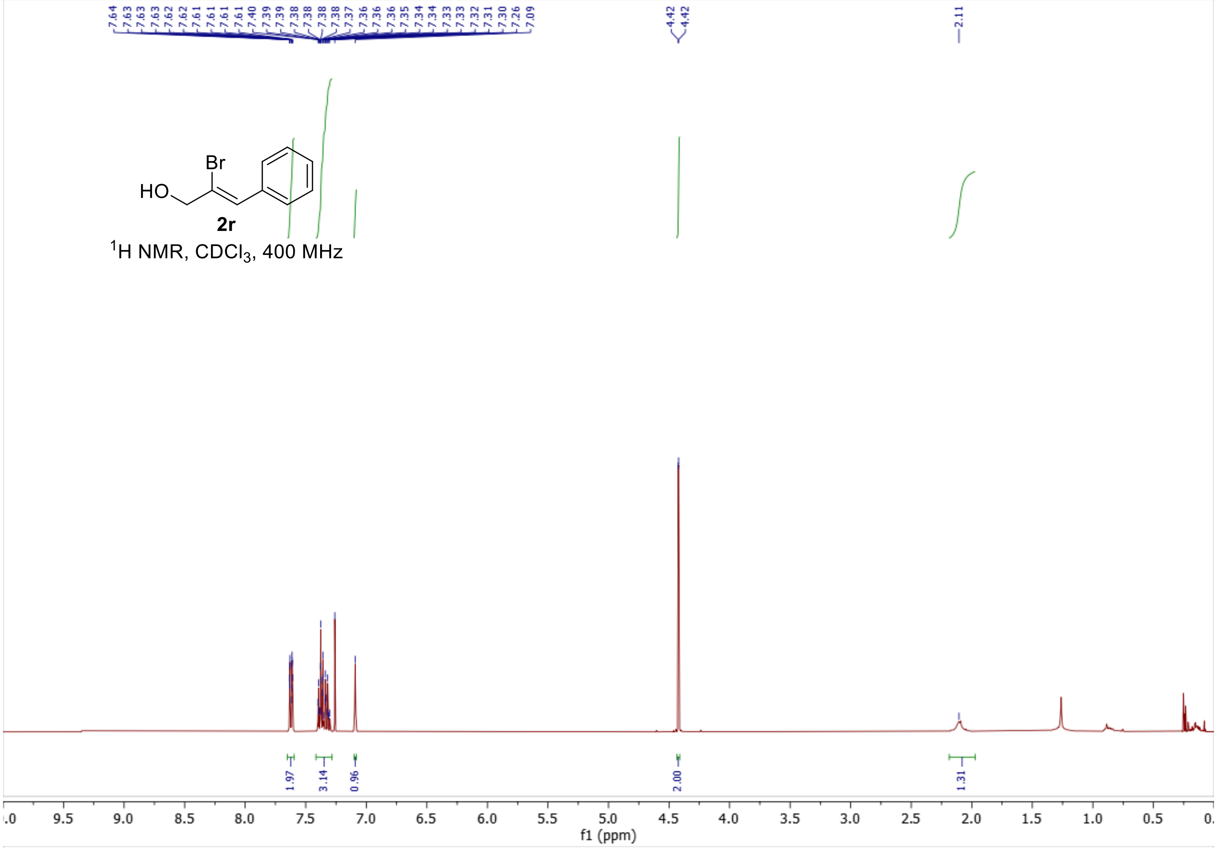
Br
HO
2r
^{1}H NMR, CDCl$_3$, 400 MHz
7.64 7.63 7.63 7.63 7.62 7.62 7.61 7.61 7.61 7.61 7.40 7.39 7.39 7.38 7.38 7.38 7.38 7.37 7.36 7.36 7.36 7.35 7.34 7.34 7.33 7.33 7.32 7.31 7.30 7.26 7.09
4.42 4.42
2.11
1.97
3.14
0.96
2.00
1.31
f1 (ppm)

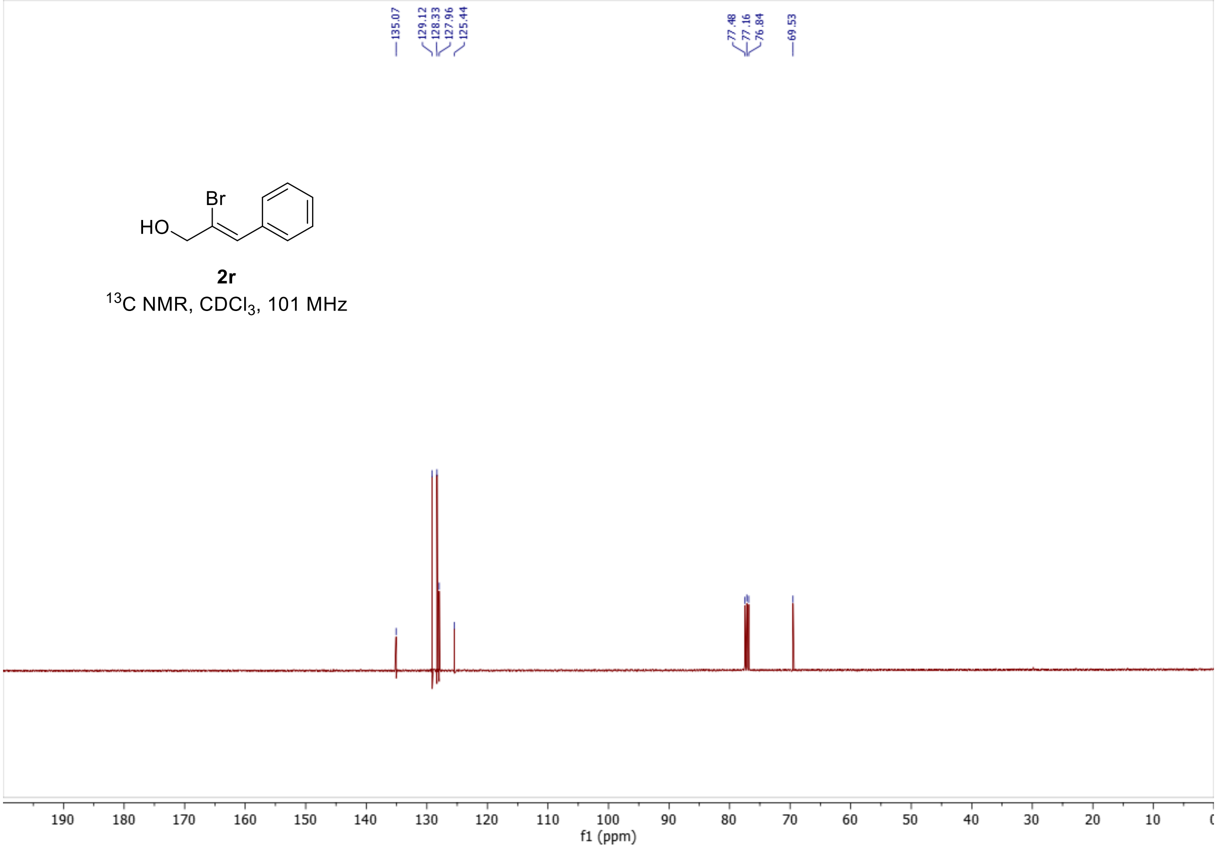
135.07
129.12
128.33
127.96
125.44
77.48
77.16
76.84
69.53
Br
HO
2r
^{13}C NMR, CDCl$_3$, 101 MHz
f1 (ppm)

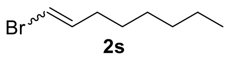

2s

^{1}H NMR, $CDCl_3$, 400 MHz

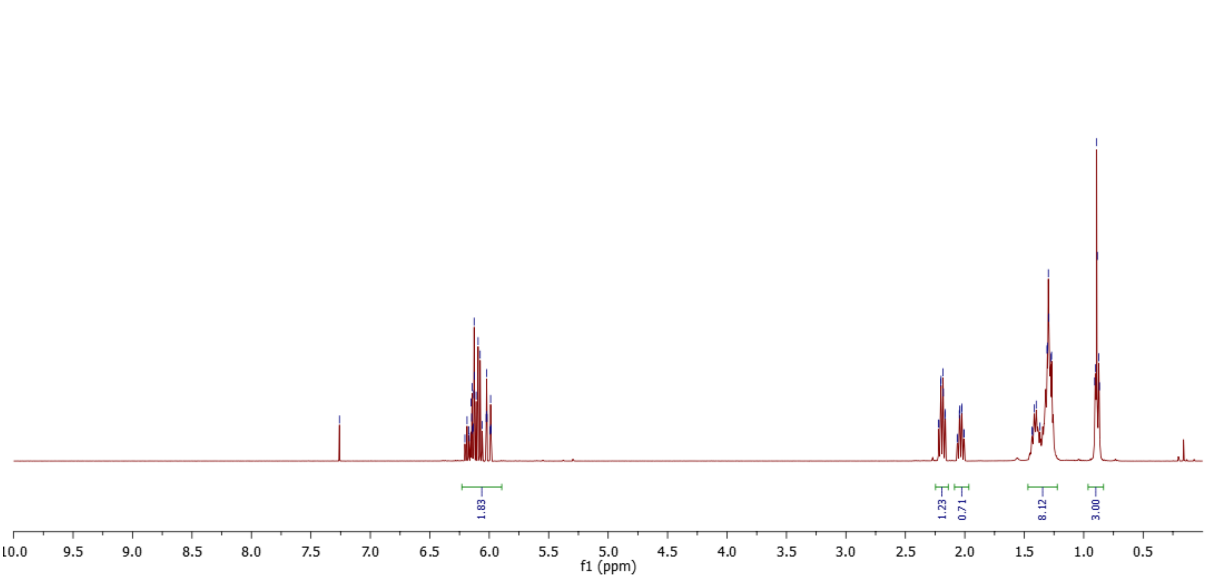

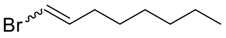

2s

^{13}C NMR, $CDCl_3$, 101 MHz

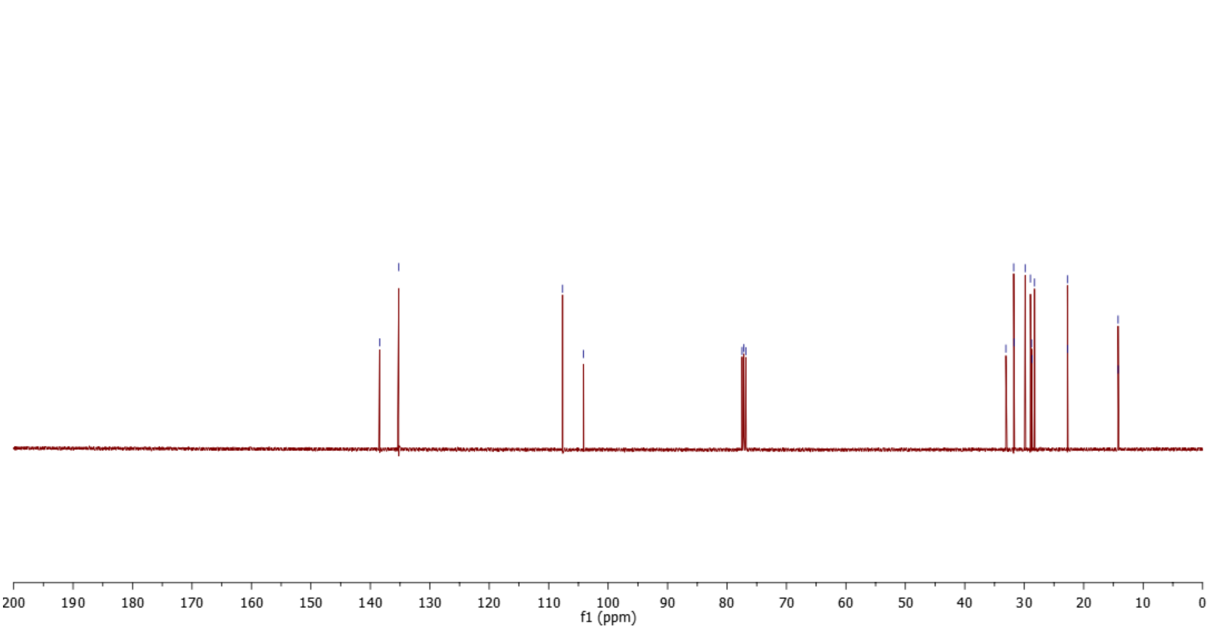

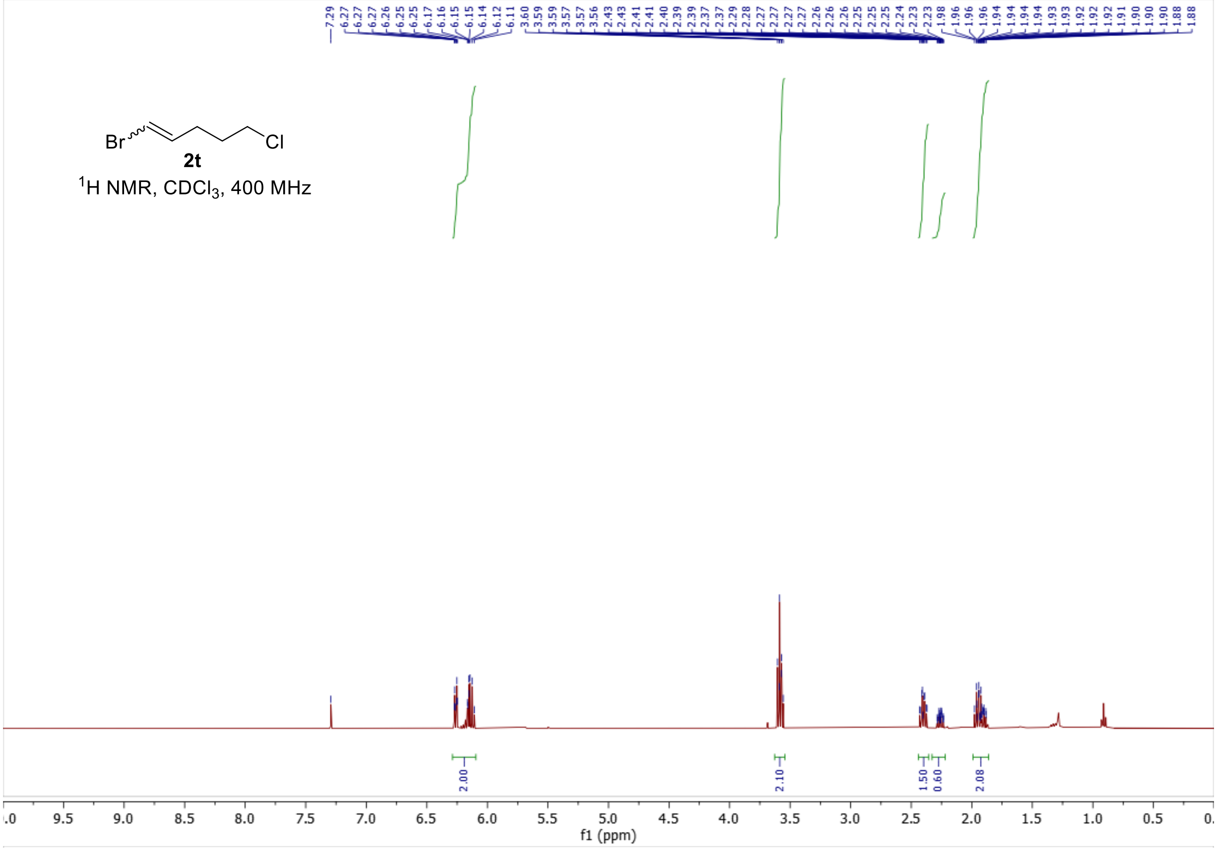
7.29
6.27 6.27 6.27 6.26 6.25 6.25 6.17 6.16 6.15 6.15 6.14 6.12 6.11
3.60 3.59 3.59 3.57 3.57 3.56
2.43 2.43 2.41 2.41 2.40 2.39 2.39 2.37 2.37 2.29 2.28 2.27 2.27 2.27 2.27 2.26 2.26 2.26 2.25 2.25 2.25 2.24 2.23 2.23
1.98 1.96 1.96 1.96 1.94 1.94 1.94 1.94 1.93 1.93 1.92 1.92 1.92 1.91 1.90 1.90 1.90 1.88 1.88
Br
Cl
2t
^{1}H NMR, $CDCl_3$, 400 MHz
2.00
2.10
1.50
0.60
2.08
f1 (ppm)

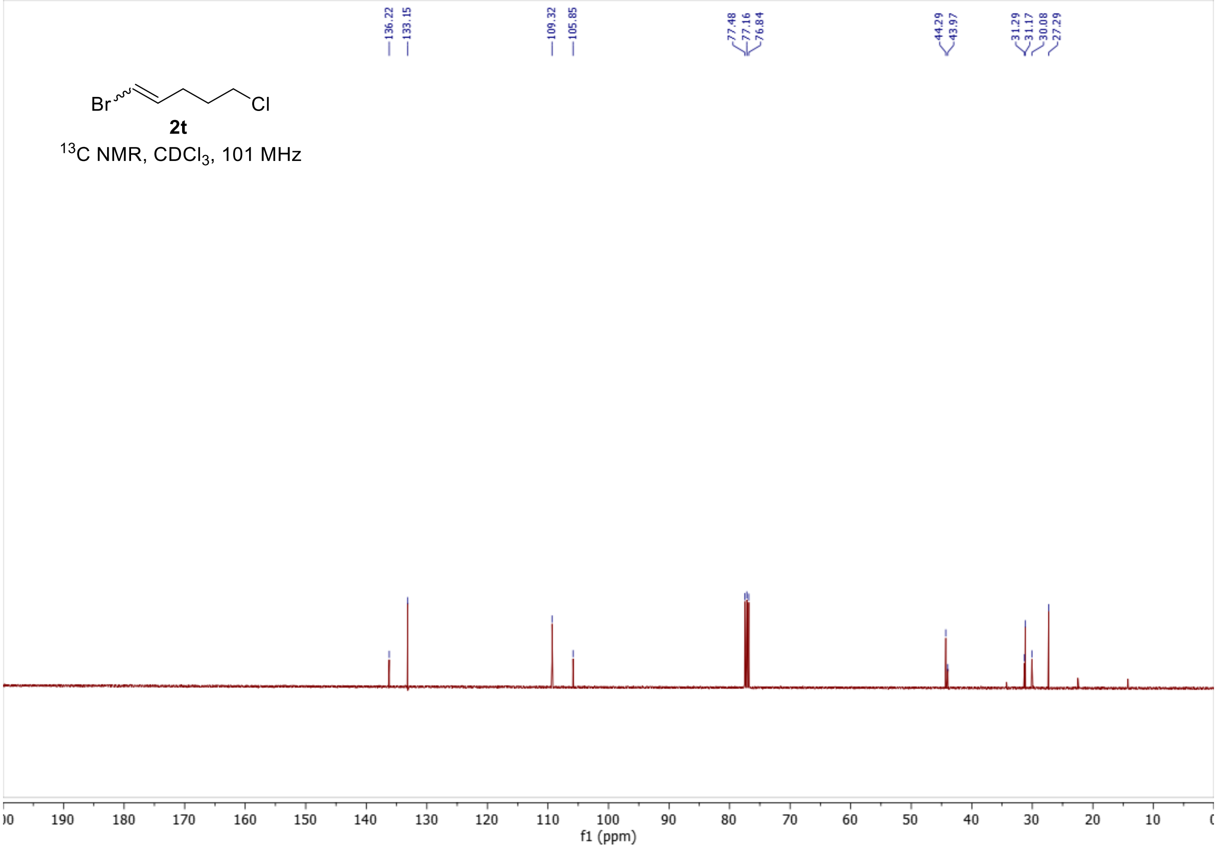
136.22
133.15
109.32
105.85
77.48
77.16
76.84
44.29
43.97
31.29
31.17
30.08
27.29
Br
Cl
2t
^{13}C NMR, $CDCl_3$, 101 MHz
f1 (ppm)

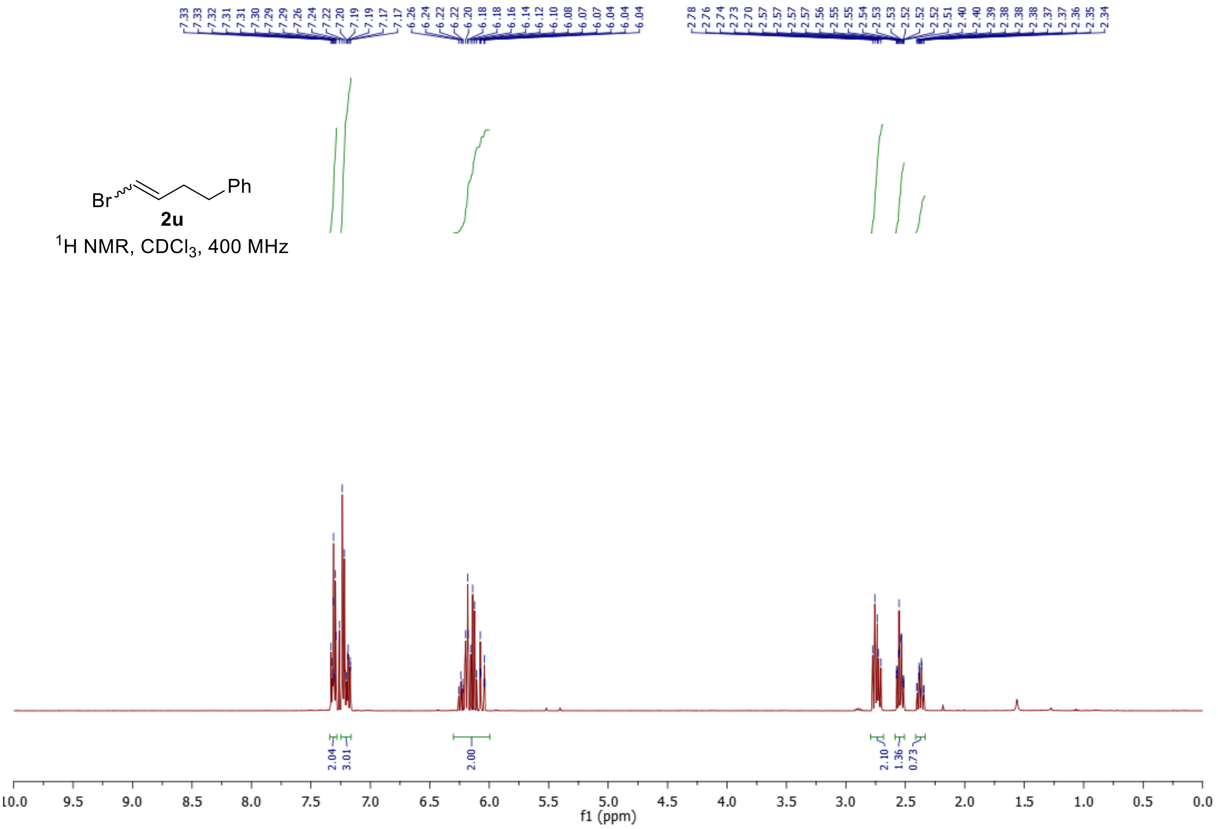
Br ~ Ph
2u
1H NMR, CDCl3, 400 MHz
f1 (ppm)

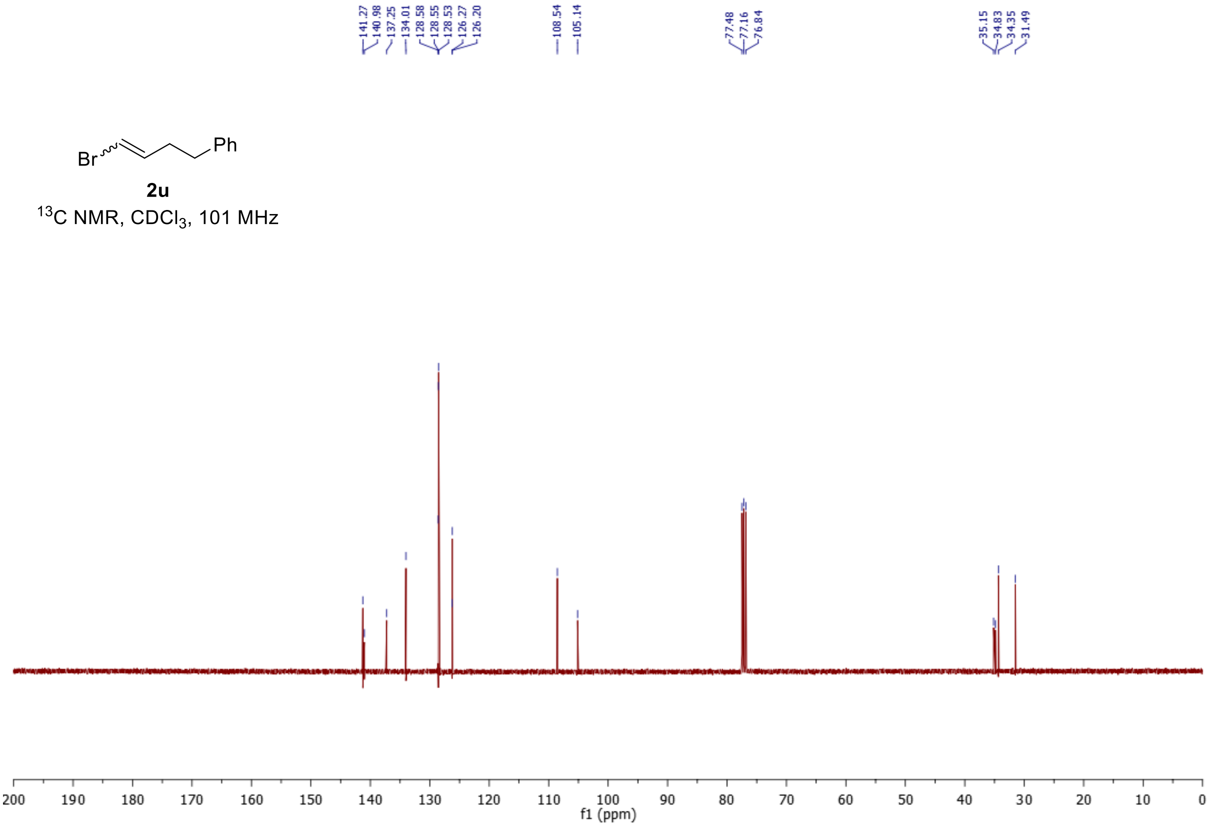
Br ~ Ph
2u
13C NMR, CDCl3, 101 MHz
f1 (ppm)

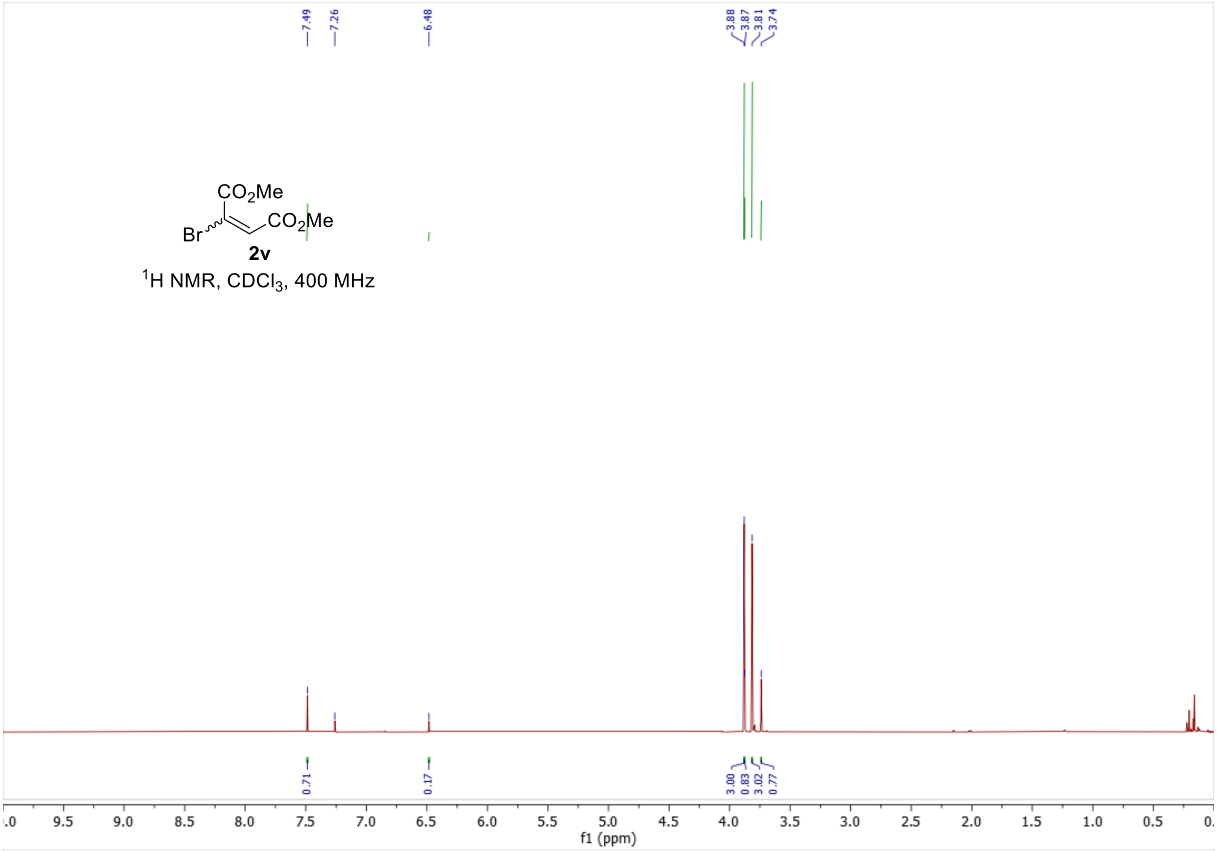
7.49
7.26
6.48
3.88
3.87
3.81
3.74
CO2Me
Br
CO2Me
2v
1H NMR, CDCl3, 400 MHz
0.71
0.17
3.00
0.83
3.02
0.77
f1 (ppm)

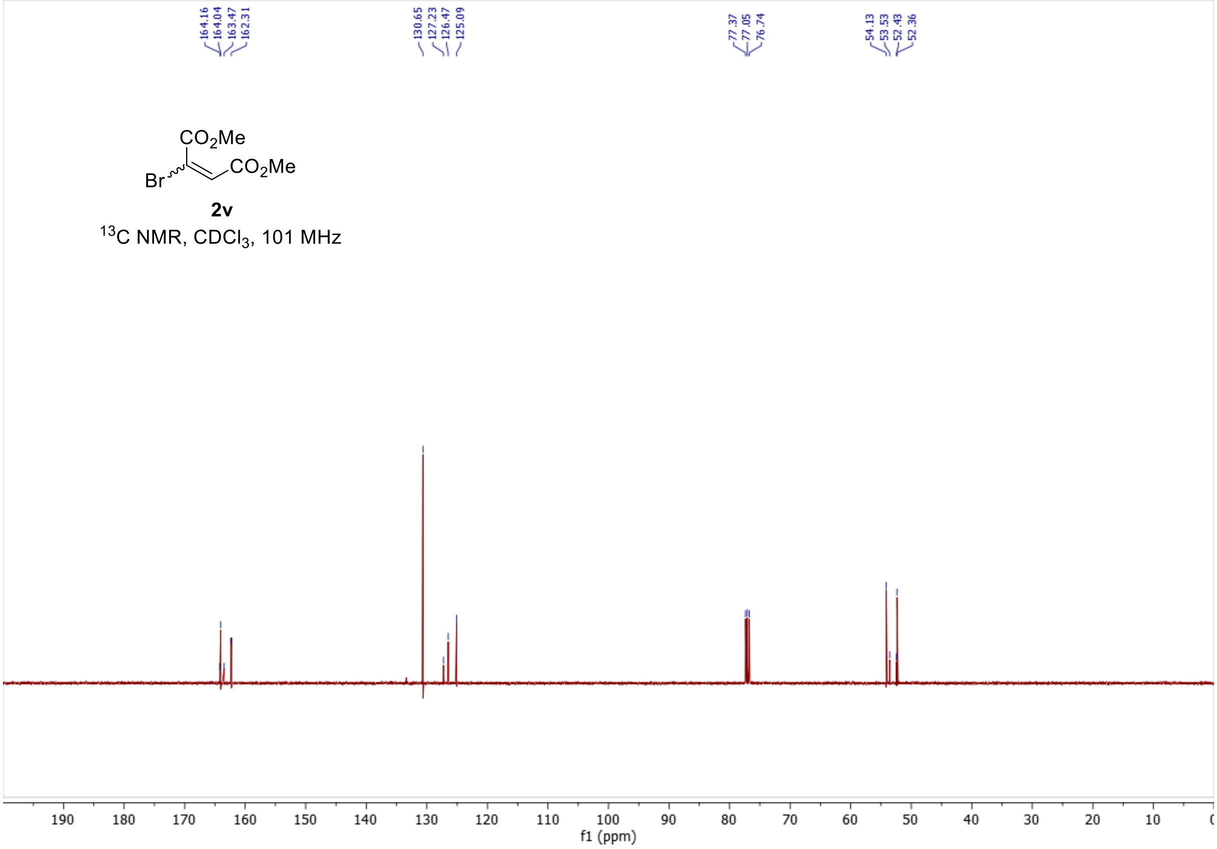
164.16
164.04
163.47
162.31
130.65
127.23
126.47
125.09
77.37
77.05
76.74
54.13
53.53
52.43
52.36
CO2Me
Br
CO2Me
2v
13C NMR, CDCl3, 101 MHz
f1 (ppm)

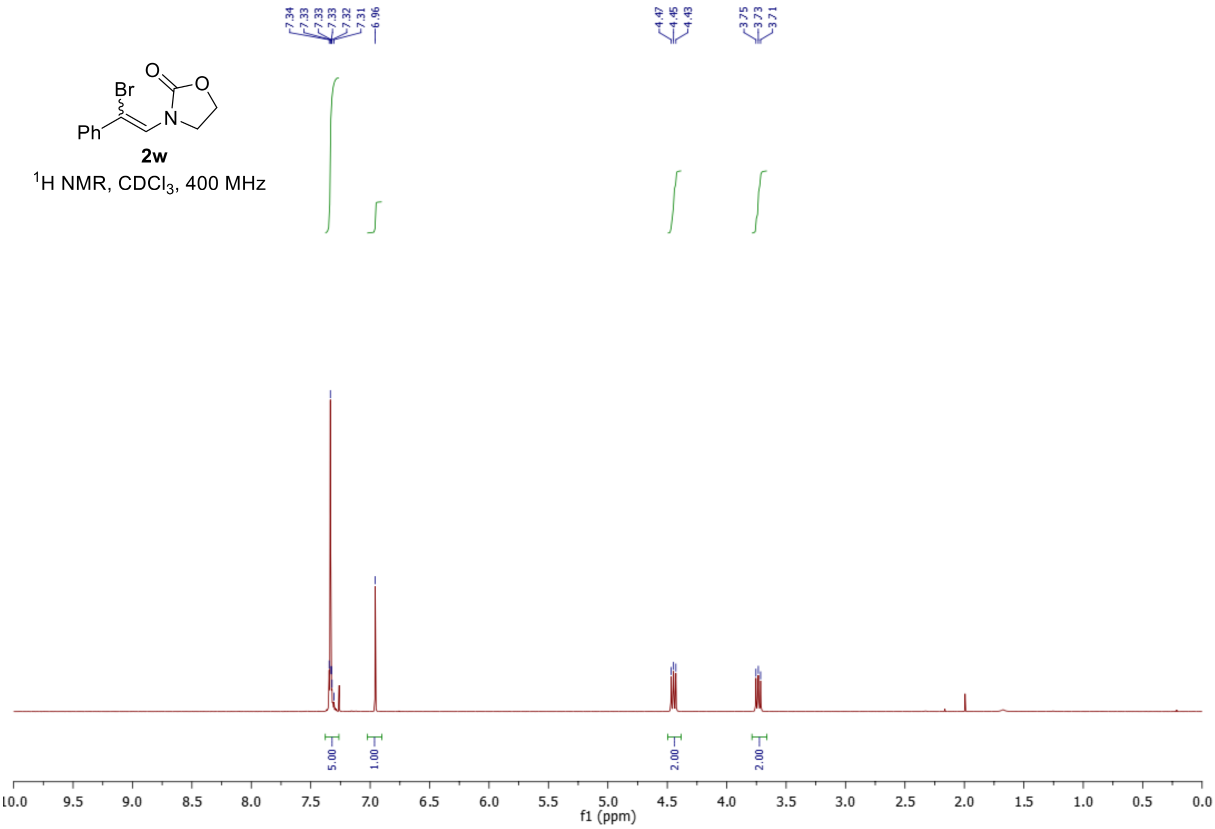

2w

^{1}H NMR, $CDCl_3$, 400 MHz

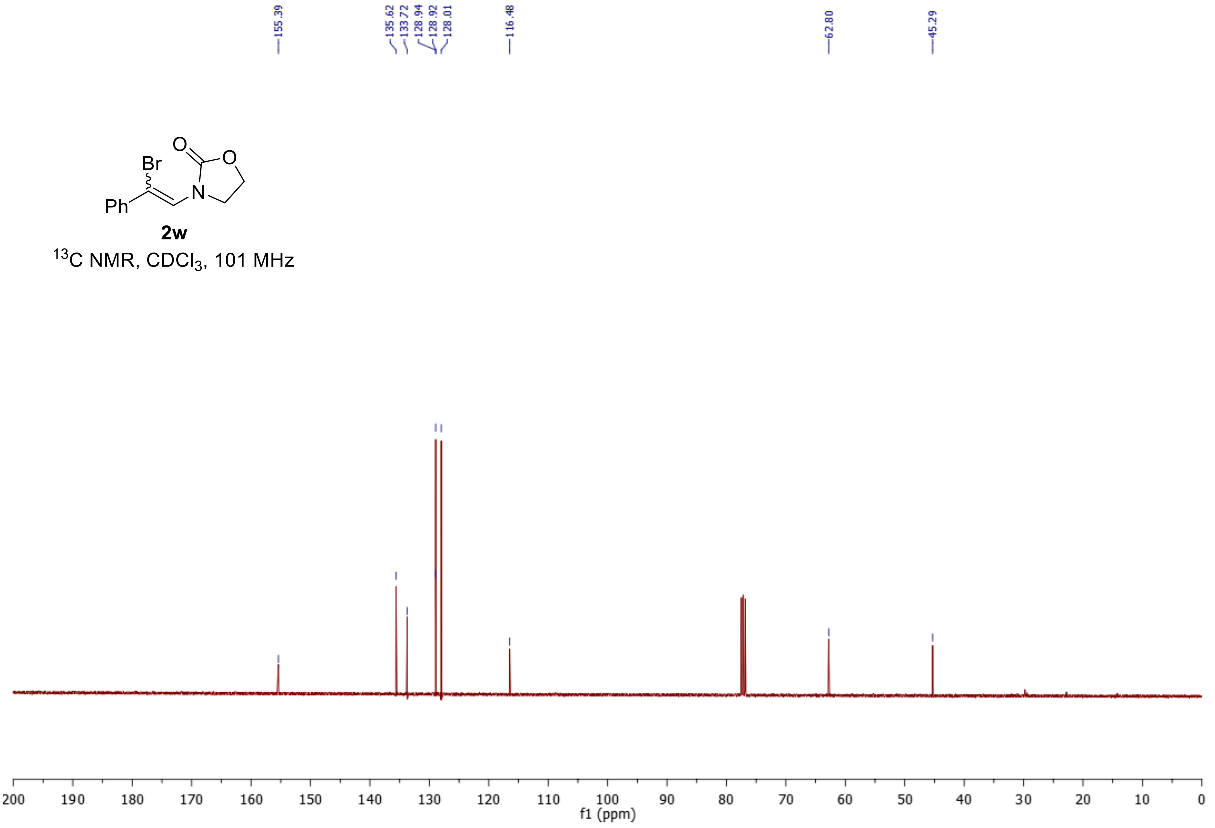

2w

^{13}C NMR, $CDCl_3$, 101 MHz

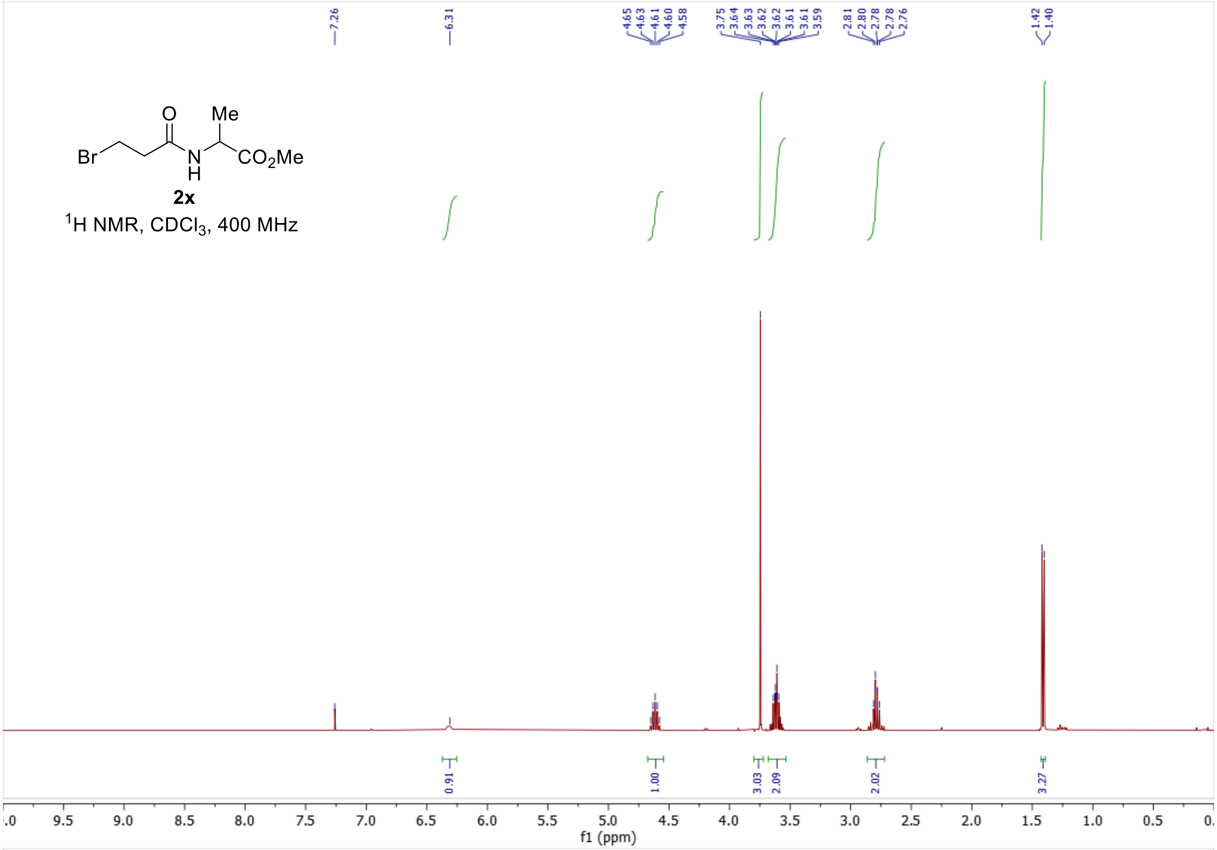
7.26
6.31
4.65 4.63 4.61 4.60 4.58
3.75 3.64 3.63 3.62 3.61 3.61 3.59
2.81 2.80 2.78 2.78 2.76
1.42 1.40
O
Me
Br
N
H
CO2Me
2x
1H NMR, CDCl3, 400 MHz
0.91
1.00
3.03
2.09
2.02
3.27
f1 (ppm)

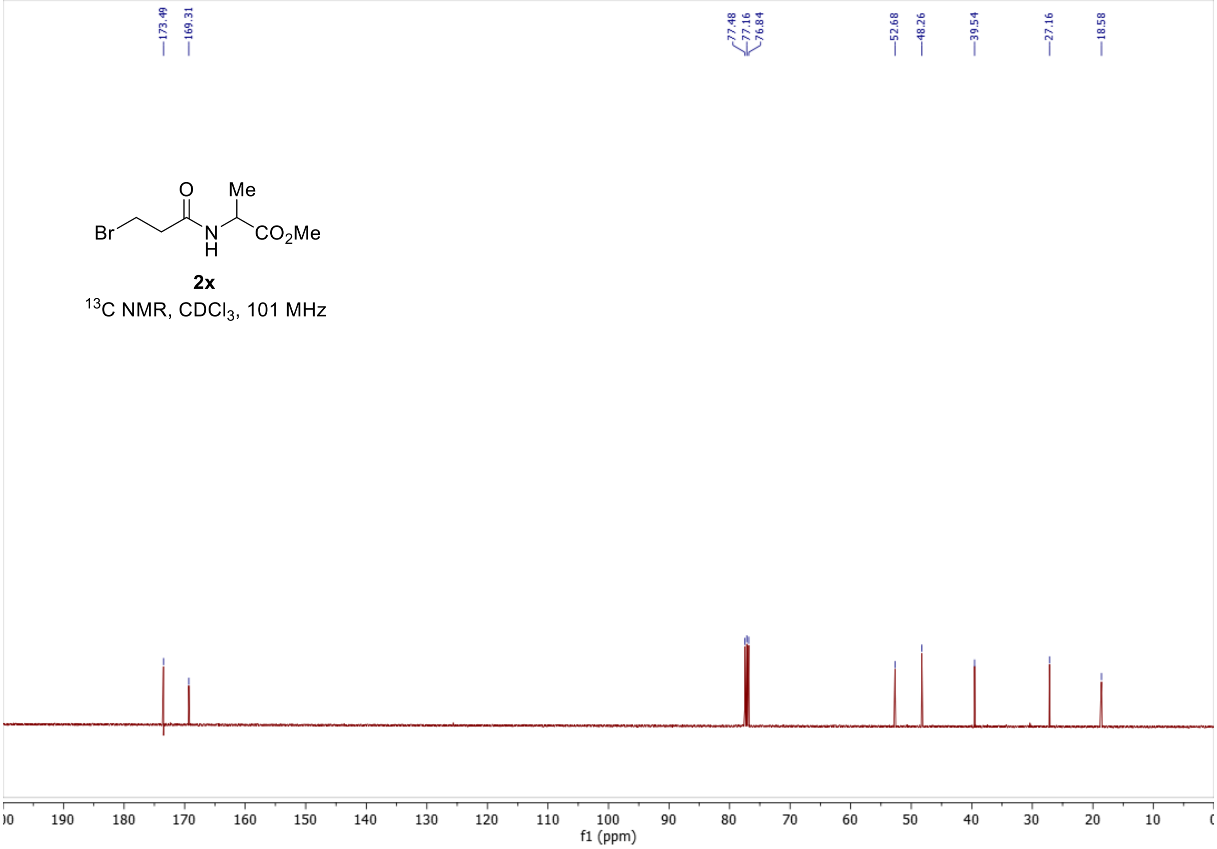
173.49
169.31
77.48 77.16 76.84
52.68
48.26
39.54
27.16
18.58
O
Me
Br
N
H
CO2Me
2x
13C NMR, CDCl3, 101 MHz
f1 (ppm)

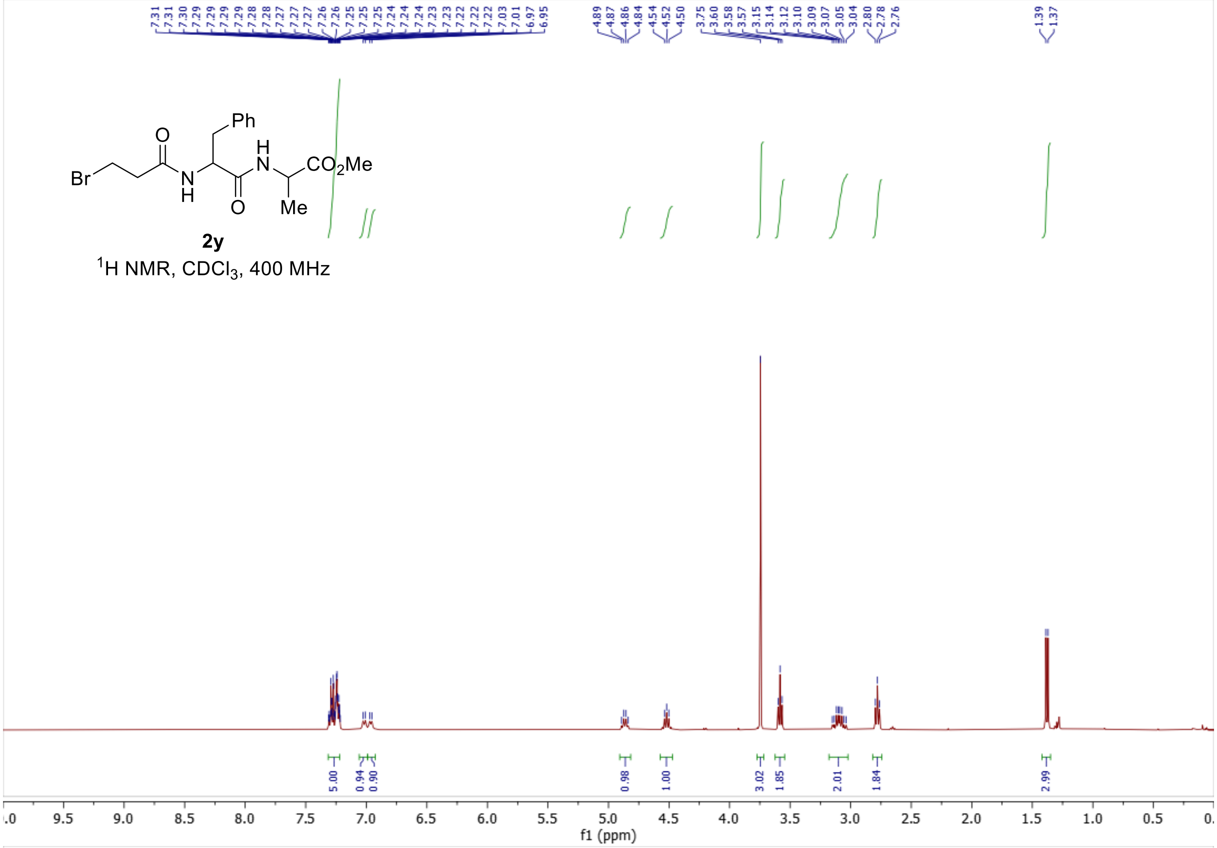
2y
1H NMR, CDCl3, 400 MHz
f1 (ppm)

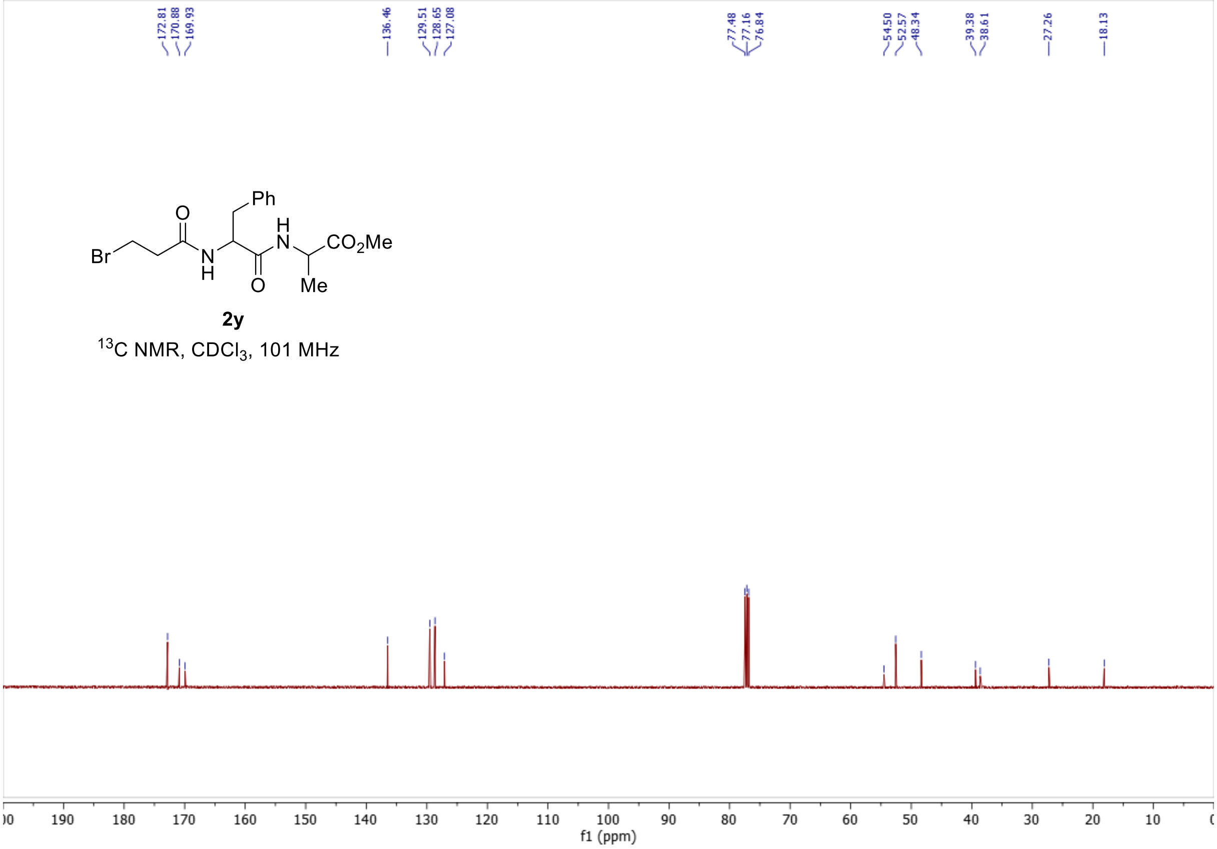
2y
13C NMR, CDCl3, 101 MHz
f1 (ppm)

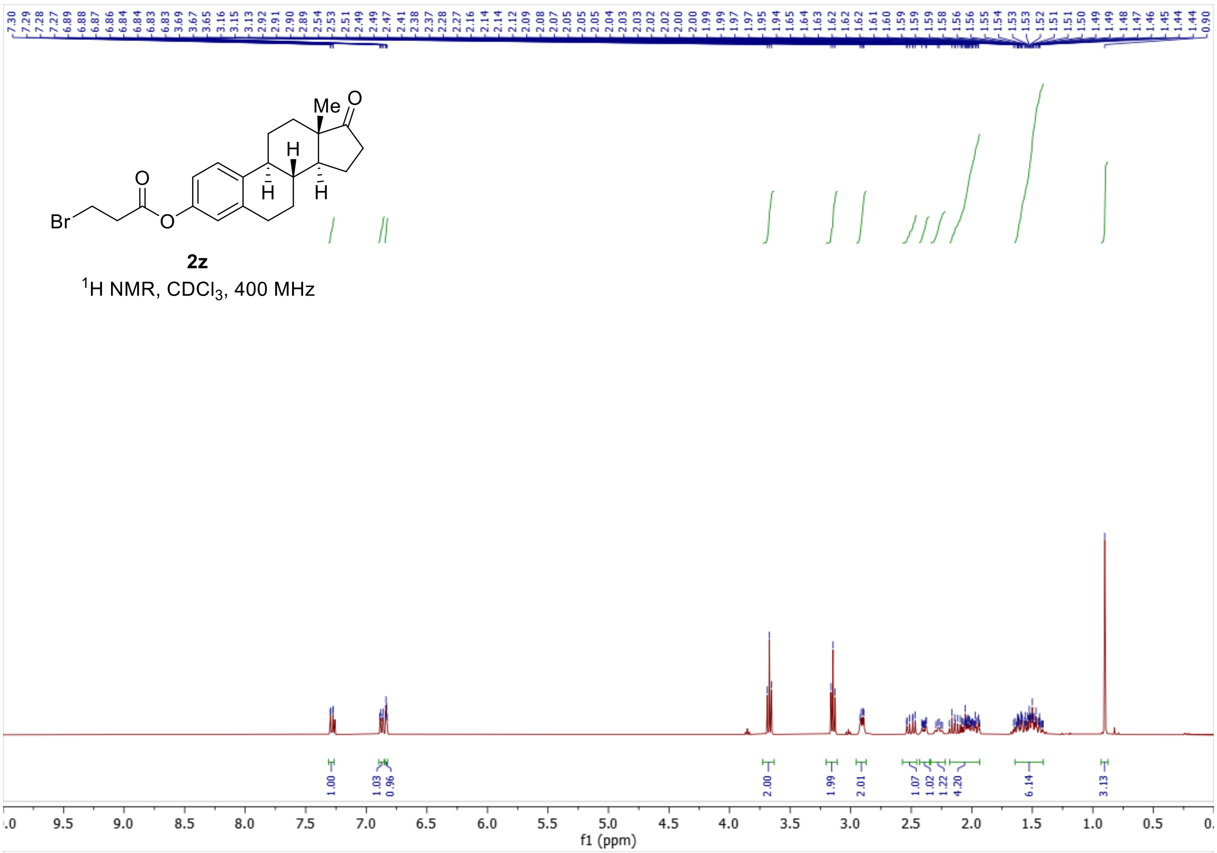
Me
O
H
H
H
O
Br
O
2z
^{1}H NMR, CDCl3, 400 MHz
f1 (ppm)

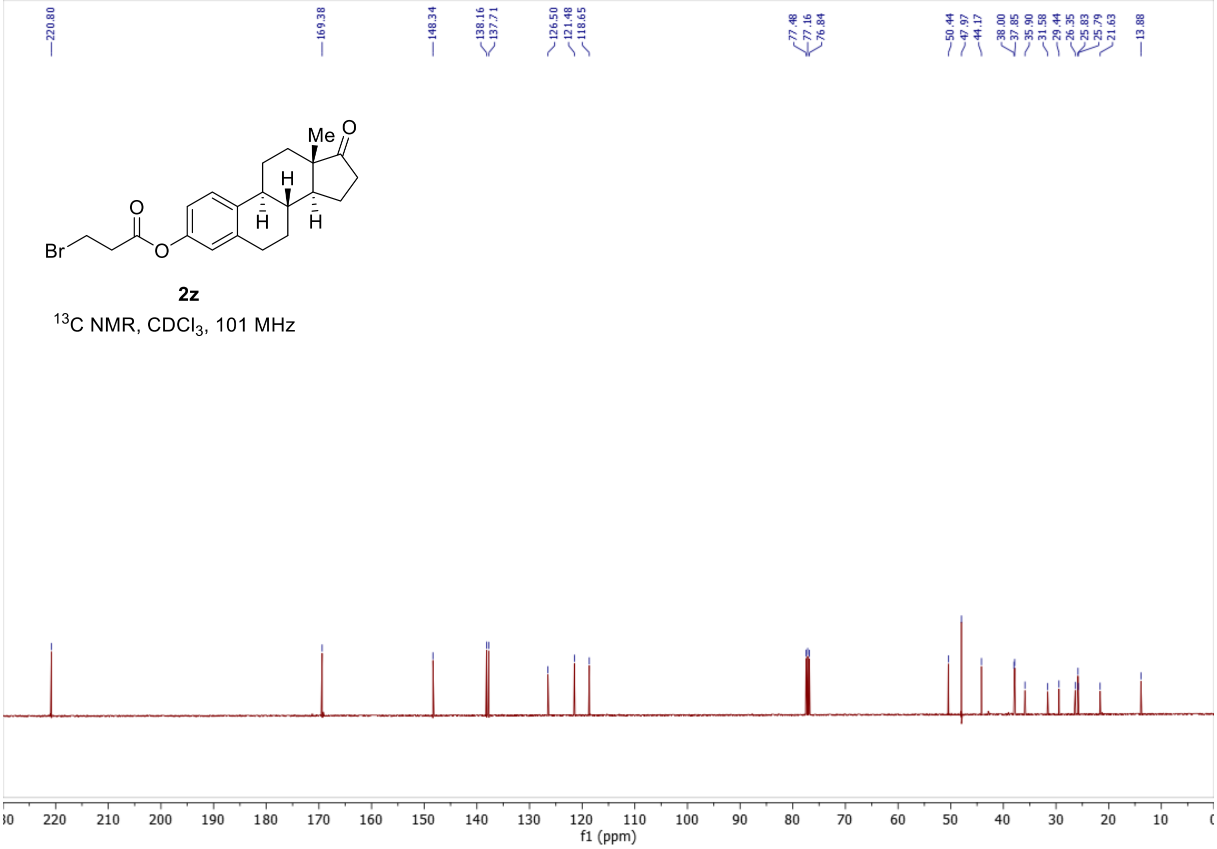
Me
O
H
H
H
O
Br
O
2z
^{13}C NMR, CDCl3, 101 MHz
f1 (ppm)

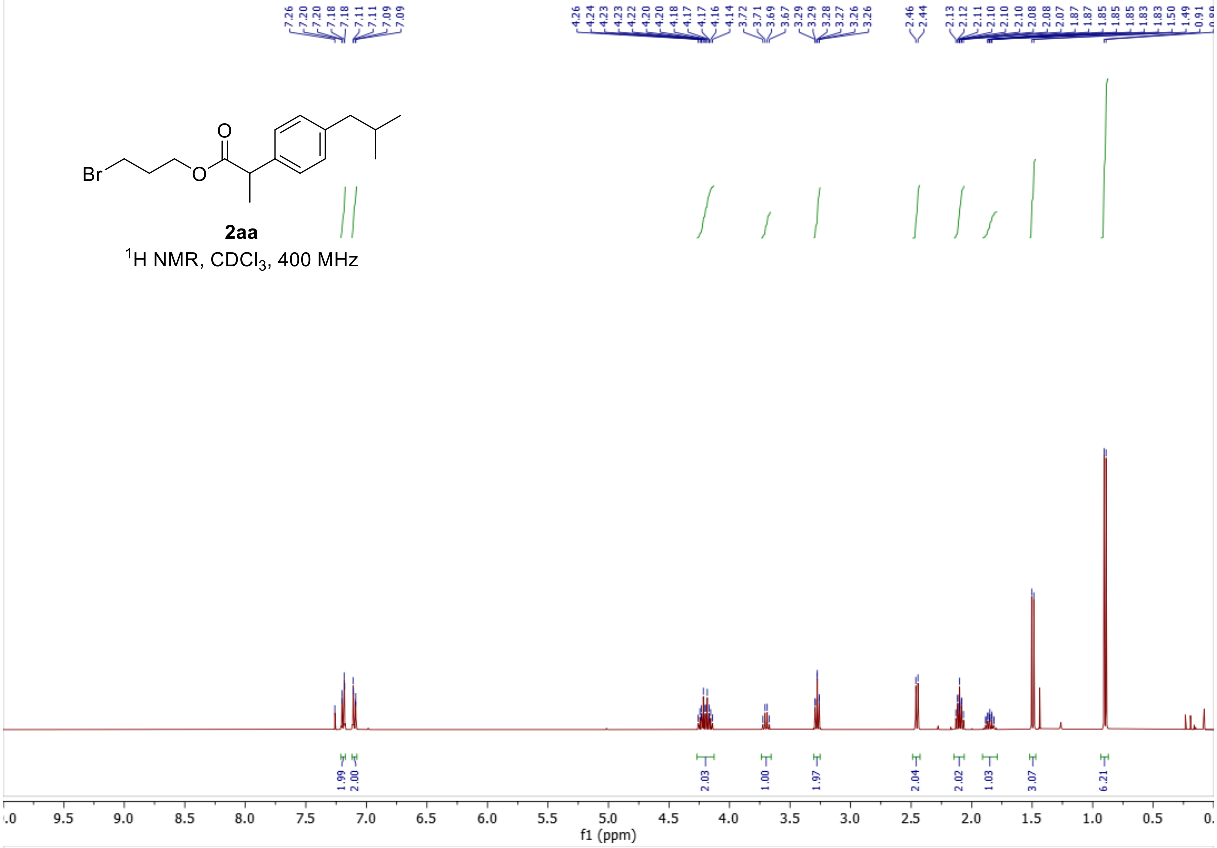
2aa
^{1}H NMR, CDCl$_3$, 400 MHz
f1 (ppm)

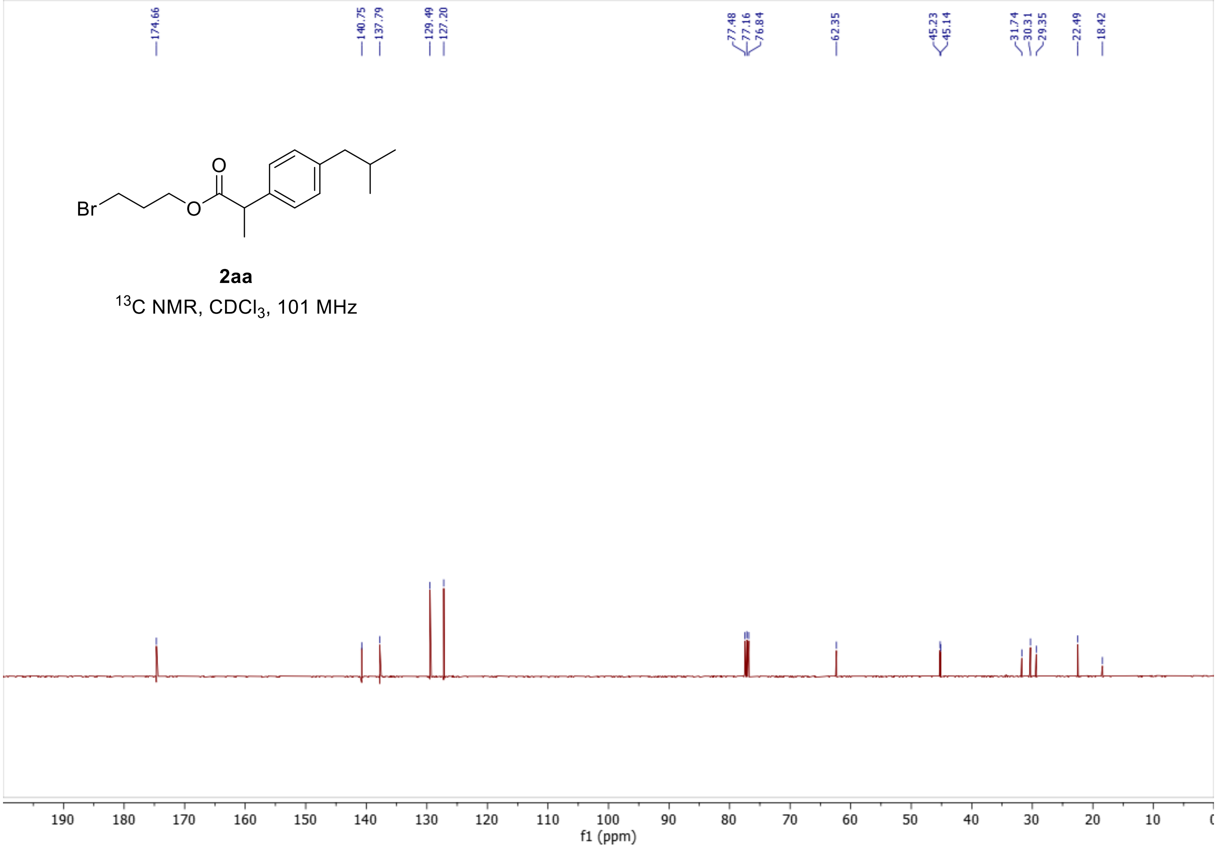
2aa
^{13}C NMR, CDCl$_3$, 101 MHz
f1 (ppm)

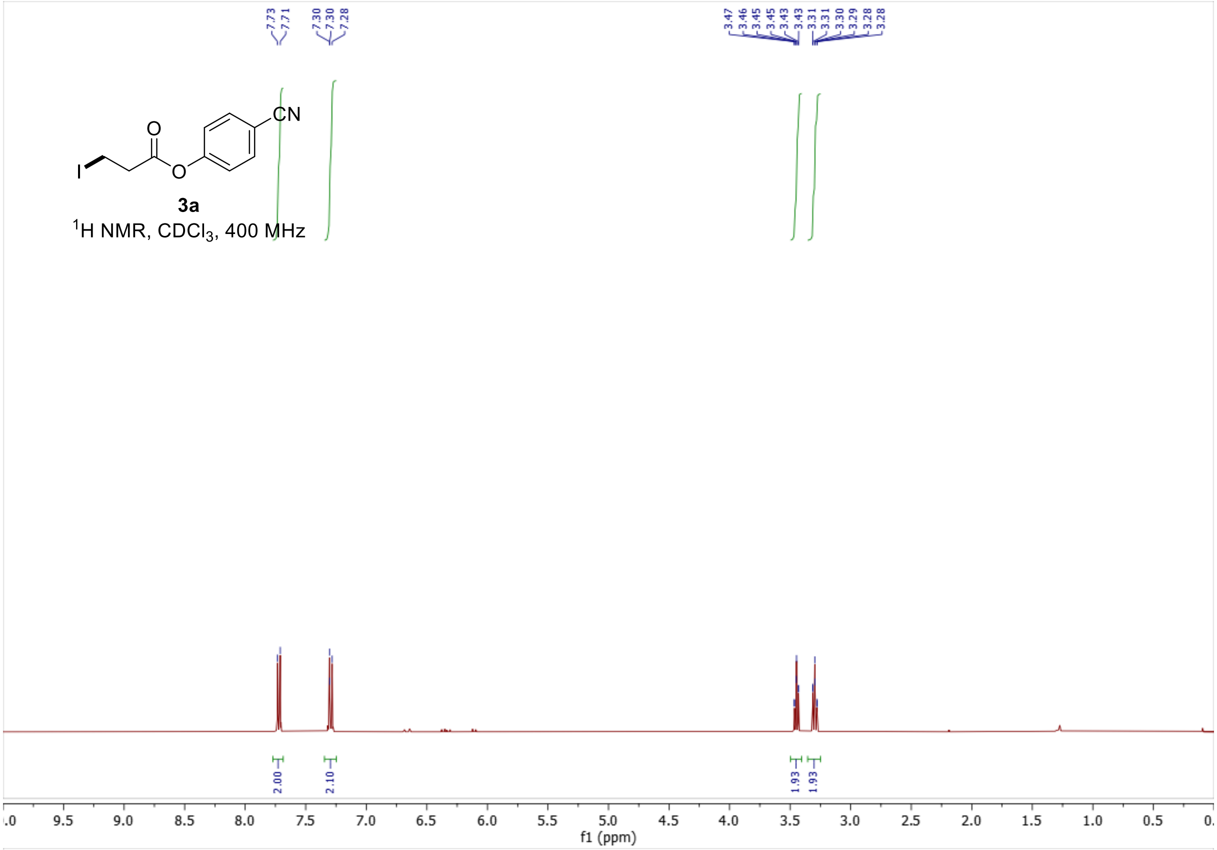
7.73
7.71
7.30
7.30
7.28
3.47
3.46
3.45
3.45
3.43
3.43
3.31
3.31
3.30
3.29
3.28
3.28
CN
O
I
O
3a
1H NMR, CDCl3, 400 MHz
2.00
2.10
1.93
1.93
f1 (ppm)

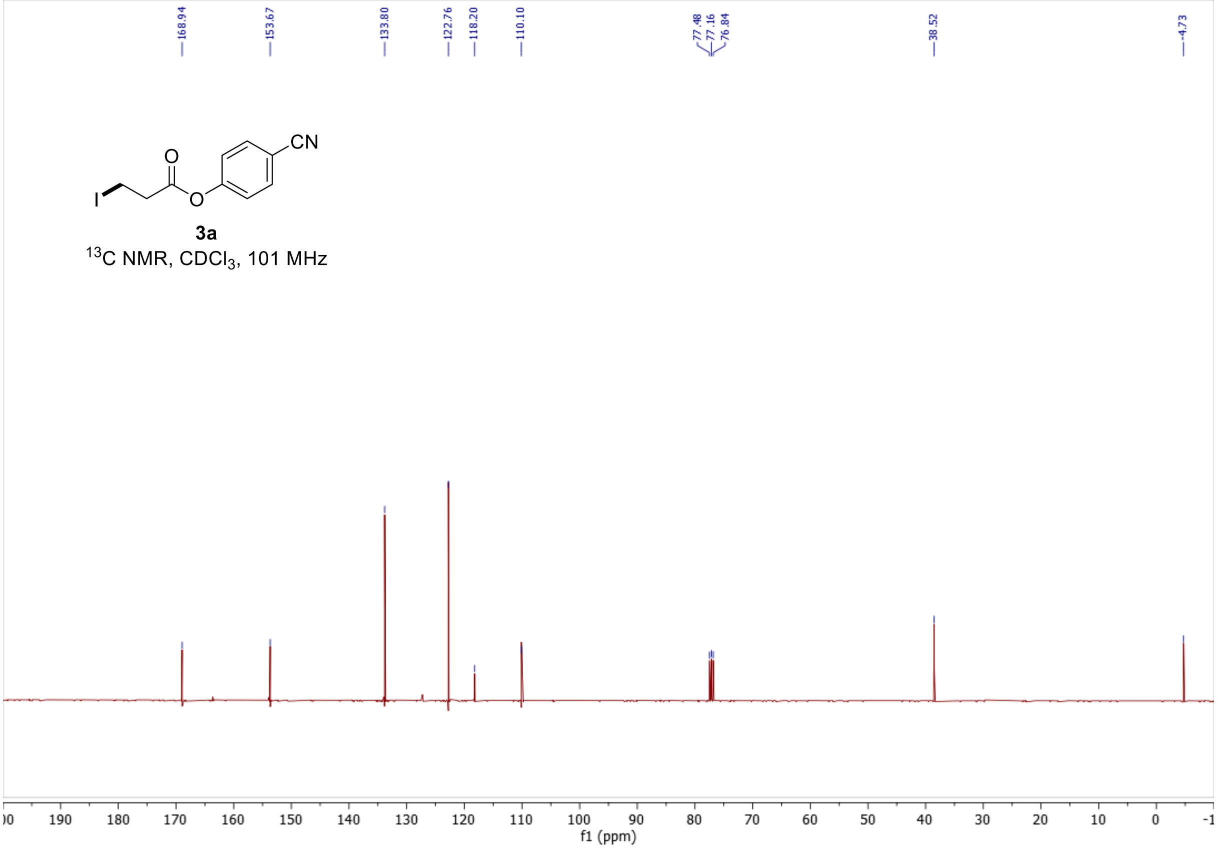
168.94
153.67
133.80
122.76
118.20
110.10
77.48
77.16
76.84
38.52
-4.73
CN
O
I
O
3a
13C NMR, CDCl3, 101 MHz
f1 (ppm)

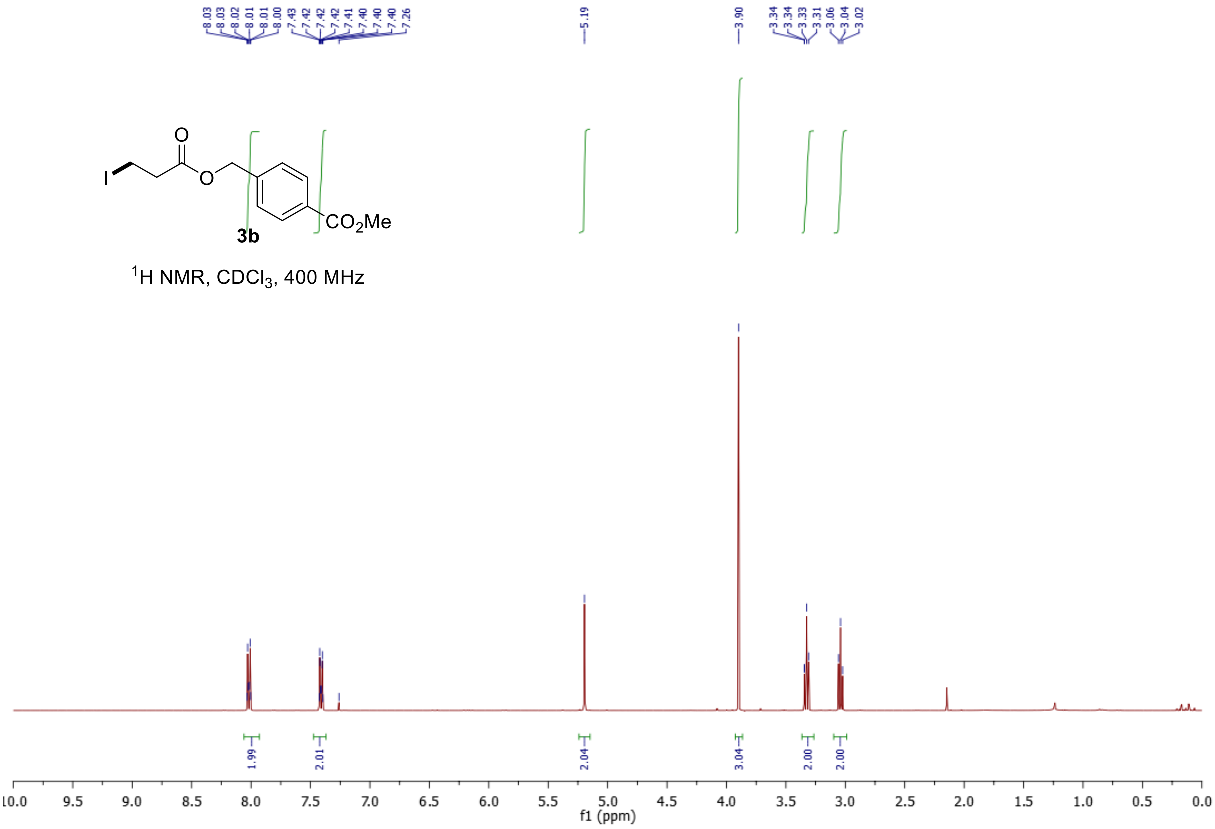
8.03
8.03
8.02
8.01
8.01
8.00
7.43
7.42
7.42
7.42
7.41
7.40
7.40
7.40
7.40
7.26
5.19
3.90
3.34
3.34
3.33
3.31
3.06
3.04
3.02
I
O
O
CO2Me
3b
1H NMR, CDCl3, 400 MHz
1.99
2.01
2.04
3.04
2.00
2.00
f1 (ppm)

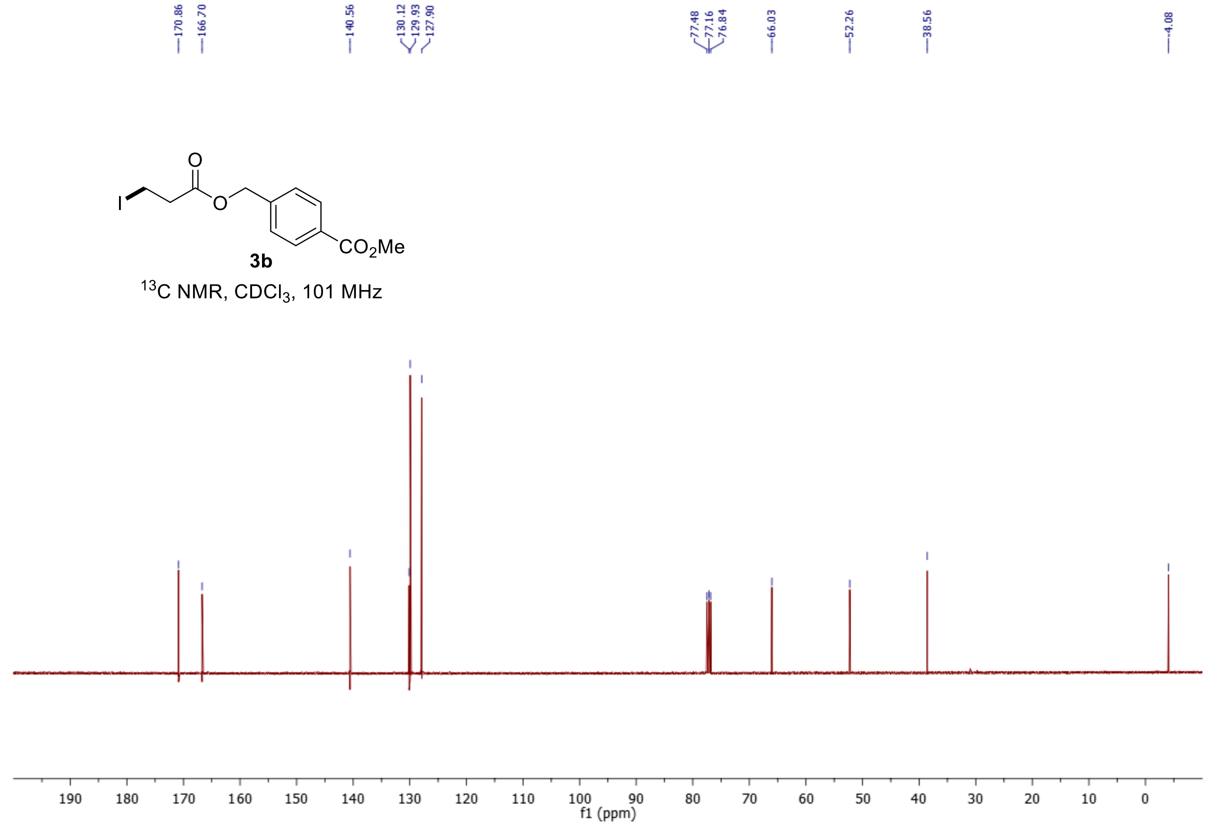
170.86
166.70
140.56
130.12
129.93
127.90
77.48
77.16
76.84
66.03
52.26
38.56
-4.08
I
O
O
CO2Me
3b
13C NMR, CDCl3, 101 MHz
f1 (ppm)

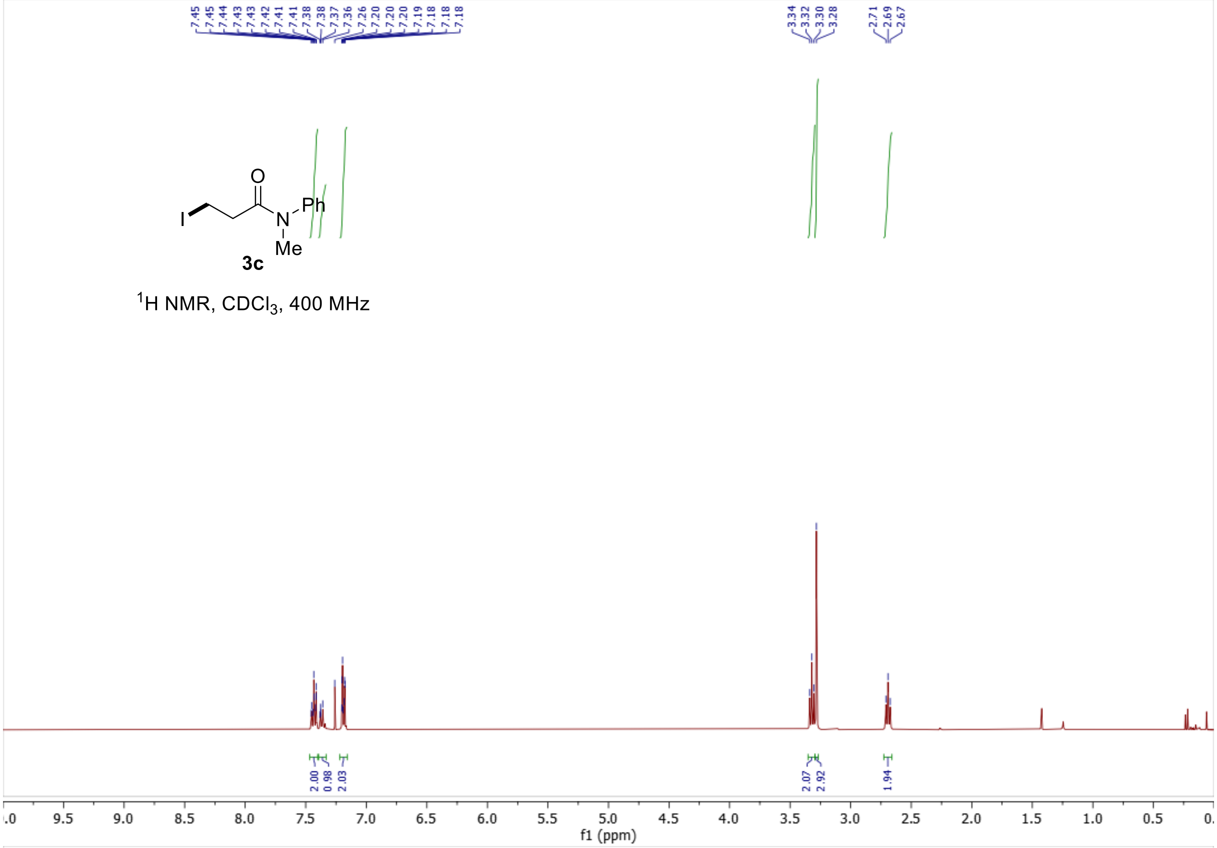
7.45
7.45
7.44
7.43
7.43
7.42
7.41
7.41
7.38
7.38
7.37
7.36
7.26
7.20
7.20
7.20
7.19
7.18
7.18
7.18
3.34
3.32
3.30
3.28
2.71
2.69
2.67
O
Ph
I
N
Me
3c
1H NMR, CDCl3, 400 MHz
2.00
0.98
2.03
2.07
2.92
1.94
f1 (ppm)

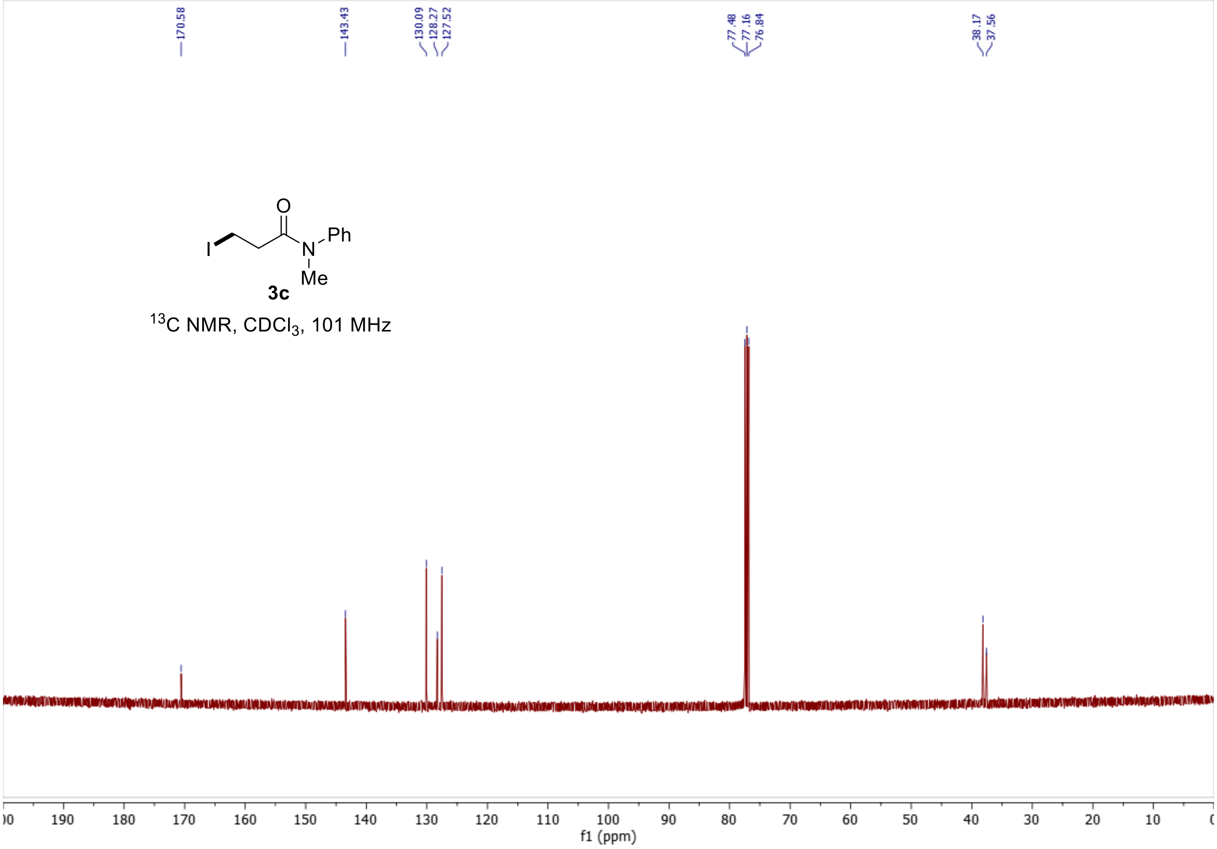
170.58
143.43
130.09
128.27
127.52
77.48
77.16
76.84
38.17
37.56
O
Ph
I
N
Me
3c
13C NMR, CDCl3, 101 MHz
f1 (ppm)

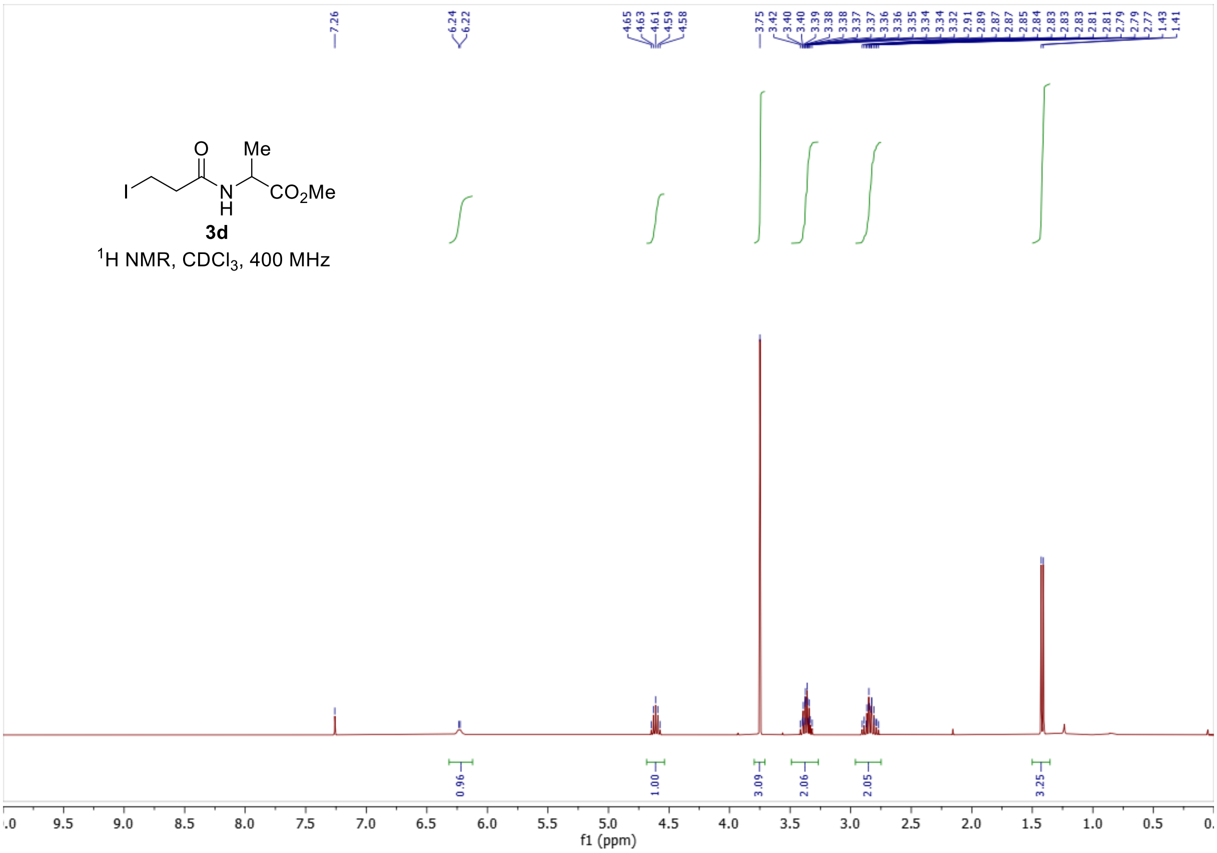
O
Me
I
N
H
CO2Me
3d
1H NMR, CDCl3, 400 MHz
f1 (ppm)

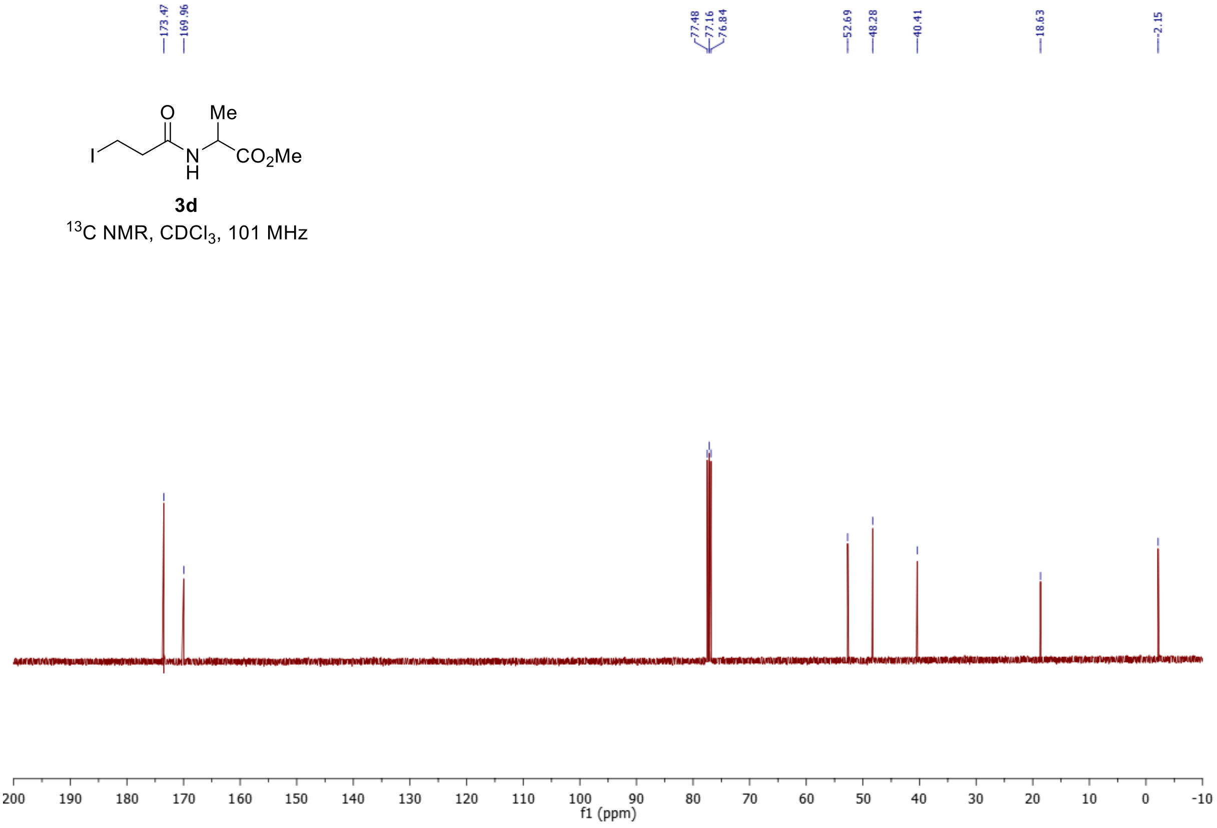
O
Me
I
N
H
CO2Me
3d
13C NMR, CDCl3, 101 MHz
f1 (ppm)

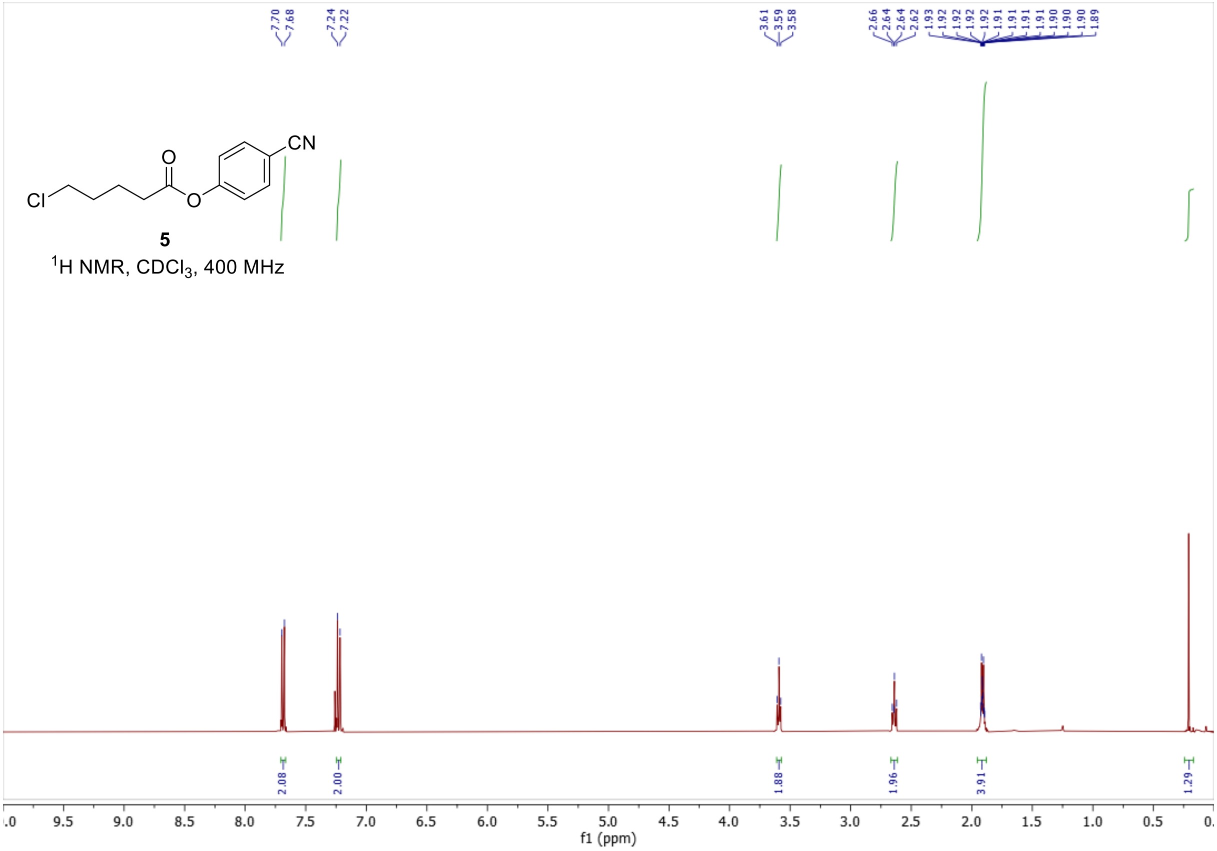
CN
O
Cl
O
5
^{1}H NMR, $CDCl_3$, 400 MHz
7.70 7.68 7.24 7.22 3.61 3.59 3.58 2.66 2.64 2.64 2.62 1.93 1.92 1.92 1.92 1.92 1.91 1.91 1.91 1.91 1.90 1.90 1.90 1.89
2.08 2.00 1.88 1.96 3.91 1.29
f1 (ppm)

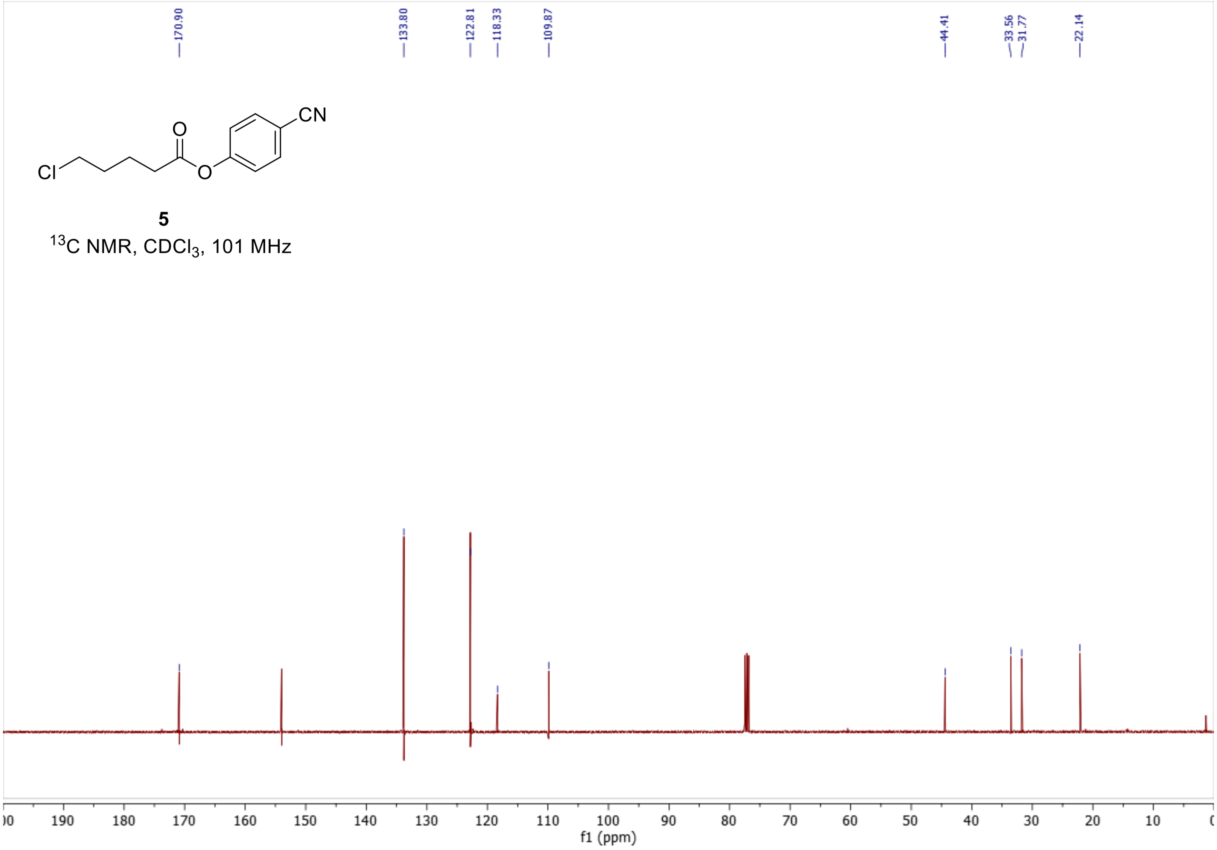
CN
O
Cl
O
5
^{13}C NMR, $CDCl_3$, 101 MHz
170.90 133.80 122.81 118.33 109.87 44.41 33.56 31.77 22.14
f1 (ppm)
